# Analysis of Human and Mouse Reprogramming of Somatic Cells to Induced Pluripotent Stem Cells. What Is in the Plate?

**DOI:** 10.1371/journal.pone.0012664

**Published:** 2010-09-17

**Authors:** Stéphanie Boué, Ida Paramonov, María José Barrero, Juan Carlos Izpisúa Belmonte

**Affiliations:** 1 Center for Regenerative Medicine in Barcelona (CMRB), Barcelona, Spain; 2 Gene Expression Laboratory, The Salk Institute for Biological Studies, La Jolla, California, United States of America; Centro de Investigación Príncipe Felipe, Spain

## Abstract

After the hope and controversy brought by embryonic stem cells two decades ago for regenerative medicine, a new turn has been taken in pluripotent cells research when, in 2006, Yamanaka's group reported the reprogramming of fibroblasts to pluripotent cells with the transfection of only four transcription factors. Since then many researchers have managed to reprogram somatic cells from diverse origins into pluripotent cells, though the cellular and genetic consequences of reprogramming remain largely unknown. Furthermore, it is still unclear whether induced pluripotent stem cells (iPSCs) are truly functionally equivalent to embryonic stem cells (ESCs) and if they demonstrate the same differentiation potential as ESCs. There are a large number of reprogramming experiments published so far encompassing genome-wide transcriptional profiling of the cells of origin, the iPSCs and ESCs, which are used as standards of pluripotent cells and allow us to provide here an in-depth analysis of transcriptional profiles of human and mouse cells before and after reprogramming. When compared to ESCs, iPSCs, as expected, share a common pluripotency/self-renewal network. Perhaps more importantly, they also show differences in the expression of some genes. We concentrated our efforts on the study of bivalent domain-containing genes (in ESCs) which are not expressed in ESCs, as they are supposedly important for differentiation and should possess a poised status in pluripotent cells, i.e. be ready to but not yet be expressed. We studied each iPSC line separately to estimate the quality of the reprogramming and saw a correlation of the lowest number of such genes expressed in each respective iPSC line with the stringency of the pluripotency test achieved by the line. We propose that the study of expression of bivalent domain-containing genes, which are normally silenced in ESCs, gives a valuable indication of the quality of the iPSC line, and could be used to select the best iPSC lines out of a large number of lines generated in each reprogramming experiment.

## Introduction

**Figure 1:**
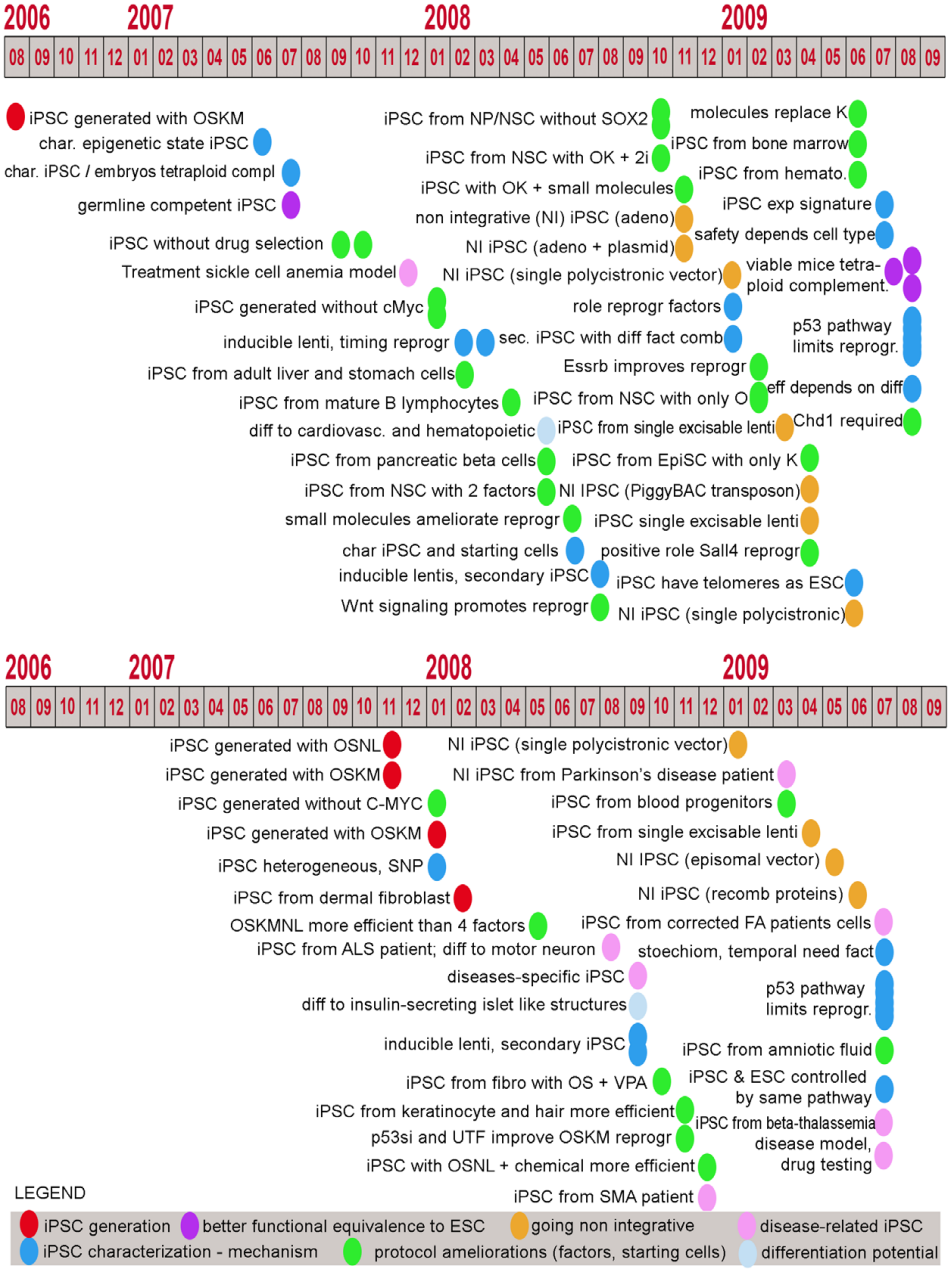
Timeline of publications in the reprogramming field. Timeline of publication of reprogramming papers in mouse and human, with a simplified classification of the main message/achievement of each paper. See supplementary File S1 for a more detailed and updated description of published reprogramming reports.

Since Yamanaka's group showed in 2006 that mouse somatic cells could be brought to a pluripotent state by transfection of only four transcription factors (Pou5f1 (Oct4), Sox2, Klf4 and c-Myc) [1] the iPSC field has generated a great deal of enthusiasm, leading to the achievement of significant advances in a relatively short period of time (see Figure 1 for a graphical overview of experiments published between 2006 and August 2009 and supplementary File S1 for a more detailed and updated list and references). Briefly, cornerstone publications in the reprogramming field have described the following attributes of iPSCs: they can be transmitted to the germ line [2], generated without the oncogenic factor c-Myc [3], [4], obtained from human cells using the same set of factors [5], [6] as well as other factors [7], obtained without permanent genomic manipulation [8], [9], [10], [11], [12], [13], produced from patient cells [14], [15], [16] even with the correction of a genetic disease [17], and more recently, a study demonstrated that iPSCs can give rise to viable mice by tetraploid complementation assays [18], [19], [20]. Similar to ESCs, iPSC lines have been shown to differentiate into derivatives of the three embryonic germ layers. More specifically, studies have demonstrated iPSC's ability to generate cells of the cardiovascular and hematopoietic lineages [21], [22], insulin-secreting islet like structures [23], functional cardiomyocytes [24], cells of the neural lineages [25], cells of the adipose lineage [26] and retinal cells [27]. Moreover, a number of papers have began to decipher the mechanisms involved in reprogramming [28], [29], [30], [31], [32], [33], [34], [35], [36], a phenomenon that will likely require significant effort in order to be fully understood.

Starting from a small biopsy of skin or even a single hair [37], cells are now routinely obtained that possess the same properties of self-renewal and pluripotency as ESCs, but overcome the ethical issues related to the use of embryos to derive ESCs. Thus, iPSCs could replace ESCs and represent an invaluable tool for regenerative medicine, as well as for the study of basic biological processes, improved understanding of diseases, and finally, as a tool for facilitating drug testing [38]. More importantly, patient-specific iPSCs could potentially be used for the same range of clinical applications as ESCs with the added advantage of overcoming the rejection risk after transplantation. However, the replacement of ESCs with iPSCs for all these applications presumes that they are as potent as ESCs in regards to their differentiation potential and most importantly, that they are at least equally safe for future clinical applications.

A significant part of the research dedicated to iPSCs has thus far focused on improving a largely inefficient and possibly unsafe reprogramming process. Several factors are taken into account to judge if a modification of the protocol brings about an improvement for reprogramming: (a) the efficiency and timing of colony appearance (b) the number and type (oncogene or not) of factors used, which might depend on the delivery method, the somatic cell type and the co-use of chemicals and (c) the absence of permanent genomic manipulation. The standard characterization of iPSC lines encompasses the verification of a rather large panel of morphological, molecular and functional attributes (see [39] for review), which is expensive and time consuming. While the necessity of full characterization for each generated iPSC line is still being debated, the number of cell lines being produced increases exponentially [40], [41]. Therefore, a simple screening method to select the best reprogrammed lines for full characterization would be extremely useful.

The reprogramming efficiency provided by different methods, defined as the number of bona fide iPSC colonies obtained per starting cell, is relatively easy to estimate, whereas assessing the quality of the generated cell lines remains approximate. While the stringency of the pluripotency tests available for mouse, reaching to the birth of mice from tetraploid complementation experiments [18], [19], [20] seems convincing, pluripotency of human cells is far less easy to prove. Indeed, the most solid pluripotency test available for human iPSCs is their ability to form teratomas. However, a recent study shows that human iPSCs lines that are not fully reprogrammed are also able to form teratomas, suggesting that this cannot be the ultimate test to judge the quality of human iPSCs [42]. Additionally, as discussed in [43], it is possible to define sub-states of pluripotency and ESCs seem to be a heterogeneous population of cells with slightly dissimilar differentiation potentials. ESCs would be able to move from one sub-state to another to form a pluripotent population overall. We set out to test whether iPSCs possess this same kind of plasticity and do not show any obvious bias towards some lineage fate due to the reprogramming process they went through or because of memory of the germ layer they originate from.

Different comparisons of genome-wide transcriptional profiles between ESC and iPSC lines have shown that they share a common pluripotency network [44], but also have a distinct expression signature [45]. These analyses however, were limited to a few reprogramming experiments. Very recently, these latest results have been challenged, showing that there is no distinct signature conserved across reprogramming experiments (neither at the gene-expression nor at the chromatin mark level for the marks H3K4me3 and H3K27me3) [46], but rather a lab-specific signature [47], or traces of cell memory [48], [49]. Using a greater number of published genome-wide transcriptional profiles of iPSCs with somatic starting cell populations and ESCs in Human and in Mouse. we highlighted similarities of iPSCs and ESCs compared to the starting somatic populations to build networks of genes consistently higher expressed in pluripotent stem cells and therefore potentially important for the reprogramming process. Although both ES and iPS cells are pluripotent, there still are some subtle differences in gene expression which may prove functionally relevant, as was shown for a locus in mouse cells [50]. Therefore we also checked the differences between iPSCs and ESCs to reveal potential functional disparities between these cells. In connection to this, we propose to study the expression levels in iPSCs of genes which are poised in ESCs: not or lowly expressed and marked by bivalent domains[51]. In ESCs, bivalent marks, characterized by the simultaneous presence of histone H3 trimethylation at lysine 4 (H3K4, a mark that usually correlates with transcriptional activation) and lysine 27 (H3K27, a mark that usually correlates with transcriptional repression), are thought to be associated with developmental genes which are usually silenced in undifferentiated cells but ready to be expressed upon differentiation and are therefore likely to play an important role in the early stages of differentiation [51] Their expression in pluripotent cells might hint at a bias towards a restricted fate during differentiation of the iPSC line, which could result in improper differentiation towards other lineages. We consider this to be a screening test for well reprogrammed iPSCs. In addition, in regards to the safety issues, we checked the expression of oncogenes and tumor suppressor genes differing in iPSCs from ESCs, and which could be the source of higher risks.

## Materials and Methods

### Gene expression analysis

The datasets used for the human analyses are: Takahashi *et al.* (GSE9561) [5]; Yu *et al.* (GSE9071) [7]; Park *et al.* (GSE9832) [6]; Zhao *et al.* (GSE12922) [52]; Masaki *et al.* (GSE9709) [33] Maherali *et al.* (GSE12390) [30]; Aasen *et al.* (GSE12583) [37]; Huangfu *et al.* (pers. comm.) [53]; Lowry *et al.* (GSE9865) [54]; Ebert *et al.* (GSE13828) [15]; Yu *et al.* (GSE15148) [55]; Soldner *et al.* (GSE14711) [11].

The datasets used for the mouse analyses are: Takahashi *et al.* (GSE5259) [1]; Okita *et al.* (GSE7841) [2]; Maherali *et al.* (GSE7815) [30]; Feng *et al.* (GSE13211) [56]; Sridharan *et al.* (GSE14012) [35]; Wernig *et al.* (E-MEXP1037) [4]; Chen *et al*. (GSE15267); Zhou *et al*. (GSE16062)[57]; Zhao *et al*. (GSE16925)[20]; Kang *et al*. (GSE17004)[19]; Heng *et al*. (GSE19023)[58]; Ichida *et al*. (GSE18286)[59]; Mikkelsen *et al*. (GSE8024) [60]; Hong *et al*. (GSE13312)[61].

Datasets coming from analyses performed on an Affymetrix platform have been renormalized using the GC-RMA algorithm[62] implemented in the R software (http://www.r-project.org/). Other datasets have been used as normalized by their respective authors.

For each dataset, the analysis was performed as follows (summarized in Figure S1): we have calculated a percentrank (pr) for each probe in each sample and each replicate. A percentrank is defined as the rank of a value in a dataset as a percentage of the dataset. This function evaluates the relative standing of a value within a data set. For microarray studies, it means that the probe with the highest intensity will get the rank 100%, whereas the probe with the lowest intensity will get the rank 0%. We estimate that the lowest 40% ranks reflect noise (as all genes of a genome are not expressed in a given cell, at a given time, and under a given condition, and based on the fact that, in ESC lines cultured in our institute, the number of presence calls by the mas5call function is around 60% when studying gene expression with the Affymetrix HGU-133 plus 2.0 platform). Next, we have introduced a weighting factor for each probe. The weight of each probe is defined as a log_2_ value of the intensity of this probe in a given sample divided by the sum of log_2_ of all probes in the sample. (As we used GC-RMA normalized data for the Affymetrix platform, which are already logarithms, we skipped this step for the Affymetrix data, and defined the weight as the GC-RMA value divided by the sum of GC-RMA values on the whole array). Then, we have calculated a weighted percent rank (wpr) for each probe in each sample and each replicate, defined as the percentrank of this probe multiplied by its weight (wpr =  pr *weight). Next, the average percentrank and the average weighted percentrank were identified for the replicates of each sample. In addition, for the dataset GSE7841 we have averaged the available iPSCs samples (day2, day16, day17 and day18). For the dataset E-MEXP-1037 we have averaged iPSCs samples (clones 8 and 18). For the dataset GSE13211 we have averaged MEF, iPSCs OSCE (clones 8 and 13) and iPSCs OSE (clones T8 and T9) samples. For the dataset GSE14012 we have averaged ESCs (v6.5 and E14), MEFs (male and female) and iPSCs (1D4 and 2D4) samples. For the dataset GSE15267 we have averaged ESCs (CGR8 and R1), iPSCs reprogrammed with four factors (S2C12 and S2C16) and iPSCs reprogrammed with 3 factors (S53C1 and S53C5). For the dataset GSE19023 we have averaged MEFs (Actin-GFP and Pou5f1-GFP) and N2SK (#3 and #11) samples. For the dataset GSE18286 we have averaged ESCs samples.

We have considered probes that have average pr below 0.4 for both ESCs and MEFs as not expressed in the experiment and have excluded them from the analysis. We have calculated the absolute difference in average wpr between ESCs and MEFs, and have ordered the probes in descending order according to that difference, so that the probes changing the most between ESCs and MEFs got the highest rank. The same procedure was also performed for the iPSCs and MEFs comparisons.

For our analysis we have decided to define a gene as a Unigene cluster. Since in many cases there are several probes corresponding to one Unigene ID, we have performed the following to keep one probe per one Unigene ID: If there was one probe corresponding to the Unigene ID, we have kept this probe for the analysis. If there were several probes corresponding to the Unigene ID, we have kept the probe with the highest rank in the ordered list for the analysis, and discarded all other probes.

For each of the comparisons (ESCs vs somatic cells, iPSCs vs somatic cells) we have selected the top 1,000 most highly ranked Unigene clusters (see supplementary File S2 for Human), and have separated them according to the gene expression change direction (up- or downregulated). To identify genes most up- or downregulated in both ESCs and iPSCs *vs*. somatic cells, we have kept only genes that are in the top 1,000 in at least 44% of available comparisons (and at least in 2 comparisons) in both ESCs and iPSCs *vs.* somatic cells.

In mouse, 346 genes are consistently upregulated in ESCs and iPSCs vs MEFs, and 462 genes are consistently downregulated in ESCs and iPSCs vs MEFs. In human, 338 and 340 Unigene clusters, respectively, were expressed higher and lower in both iPSCs and ESCs compared to fibroblasts or keratinocytes.

### Principal component analysis for each experiment

The principal component analysis to highlight the grouping of iPSCs and ESCs far from the starting cell type (with or without overlap of iPSCs and ESCs) has been conducted in R using the GC-RMA profiles of series matrix for non-Affy platforms using the pcromp function. Graphs were made using the first two components.

### Genes whose promoter is bound by diverse transcription factors in mouse ESCs

Data about the binding of nine transcription factors important for pluripotency/self renewal and reprogramming in mouse promoters of known genes (Nanog, Sox2, Dax1, Nac1, Pou5f1 (Oct4), Klf4, Zfp281, Rex1 and Myc) has been extracted from [63].

Data about the binding of polycomb-complex genes Suz12 and Eed in mouse ESCs has been extracted from [64]. For the mouse network of upregulated genes, the supplementary File S3 summarizes their chromatin marks on H3K4 and H3K27 in ESC and MEF, as well as transcription factors bound and the percentage of comparisons in which they have been in the top 1000 changes.

### Analysis of bivalent domain-containing genes in human ESCs

In order to evaluate the expression of genes containing bivalent domains, and thereby the quality of the reprogrammed iPSCs compared to ESCs, we have used an overlapping set of genes from 3 genome-wide characterizations of bivalent domain containing genes [65], [66], [67] which we consider a high confidence set, and have only used the 316 Ensembl genes for which we had expression values in each dataset tested (see Figure S2). We have considered percentrank values for the comparison between iPSCs and ESCs.

The correlation coefficient between different profiles (genome-wide and for those 316 genes) was calculated using the correl function in Excel (see Figure S3).

### Functional analysis of upregulated and downregulated genes

We have investigated the function of the up- or down-regulated genes in ESCs and iPSCs vs somatic cells using DAVID [68]. Genes were organized according to biological process, molecular function and cellular component based on the Gene Ontology (GO) [69] annotations. In addition, we used the tool searching the Kyoto Encyclopedia of Genes and Genomes (KEGG) [70] database of biochemical pathways to identify pathways that are upregulated in ESCs and iPSCs, or MEFs.

We have investigated possible functional associations among upregulated and downregulated genes using STRING [71]. A network of genes that were predicted with a high confidence (STRING score 0.7 at least) as interacting partners were visualized using MEDUSA [72].

## Results and Discussion

### iPSCs and ESCs exhibit a common pluripotency network

A large effort to characterize the transcriptome of pluripotent cells has shown that a pluripotency network built from a large number of stem cells (the “stem cell matrix”) is also mostly shared by iPSCs[44]. This analysis, mostly focused on embryonic and adult stem cells, took into account only a couple of iPSC lines. To gain a more thorough insight into the functional equivalence of iPSCs and ESCs, we set out to analyze available datasets of genome-wide gene expression profiles of starting cell types versus reprogrammed cells (iPSCs) and ESCs. An important challenge that we faced during our analysis was in regards to the variability in gene expression that exists between different ESC lines [73] which seemingly does not influence their pluripotency and self-renewal capacities. Similarly, we expected iPSCs to show a certain level of variability in gene expression between each other and even more markedly than ESCs because they originate from different cell types, have been obtained with different factor combinations and delivery methods and their self renewal and pluripotency qualities are not always fully proven. Reassuringly though, performing a principal component analysis for each reprogramming experiment available, we always saw that iPSCs are much closer to ESCs than to the starting somatic cells, based on their genome-wide transcriptional profile (see PCA analysis in Figure S4). Interestingly, among the cases where more than one ESC line was available, very rarely iPSC and ESC samples are mixed. In most cases, iPSC samples cluster together away from ESC samples. This suggests than in most reprogramming experiments, although close to ESCs, iPSCs contain a gene-signature that could differentiate them from ESCs in accordance to [45] or that iPSCs and ESCs are not strictly equivalent on a transcriptome level.

Before investigating the differences between iPSCs and ESCs, we concentrated on the most consistent similarities observed between them to determine the genes and pathways that appear important for pluripotency and self-renewal and that are activated or silenced during reprogramming. 346 genes in mouse consistently showed a higher expression in both iPSCs and ESCs compared to fibroblasts and 462 genes consistently showed a lower expression level in both iPSCs and ESCs as compared to fibroblasts. In human cells, we obtained 338 and 357 Unigene clusters that were expressed at higher or lower levels, respectively, in both iPSCs and ESCs compared to the starting cell populations (see supplementary File S2 for Human). These lists of genes were extensively analyzed using protein-protein interactions data, gene ontology (see Figure S5) and literature analysis to gain insight into their functionality (see supplementary Text S1 for the functional description of the genes up-and down- regulated in Mouse ESCs and iPSCs compared to MEF).

**Figure 2:**
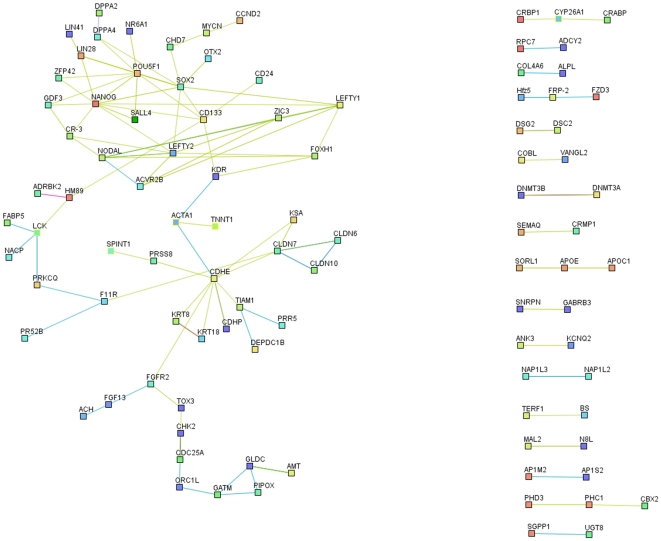
Human protein-protein interaction networks of genes with higher expression levels in ESCs and iPSCs compared to somatic cells. The human protein-protein interaction networks of genes most consistently highly expressed in ESCs and iPSCs, compared to the starting cell populations, have been created from the lists of the biggest changes in expression, using String[71] with high confidence interactions (min score 0.7) and have been edited in Medusa[72]. They show a central, highly interconnected network of genes in which the most famous pluripotency transcription factors are to be found and which is likely to represent the core pluripotency network. They also highlight a number of genes whose functions relate to cell-cell communication, cell cycle, DNA repair and other metabolisms.

**Figure 3:**
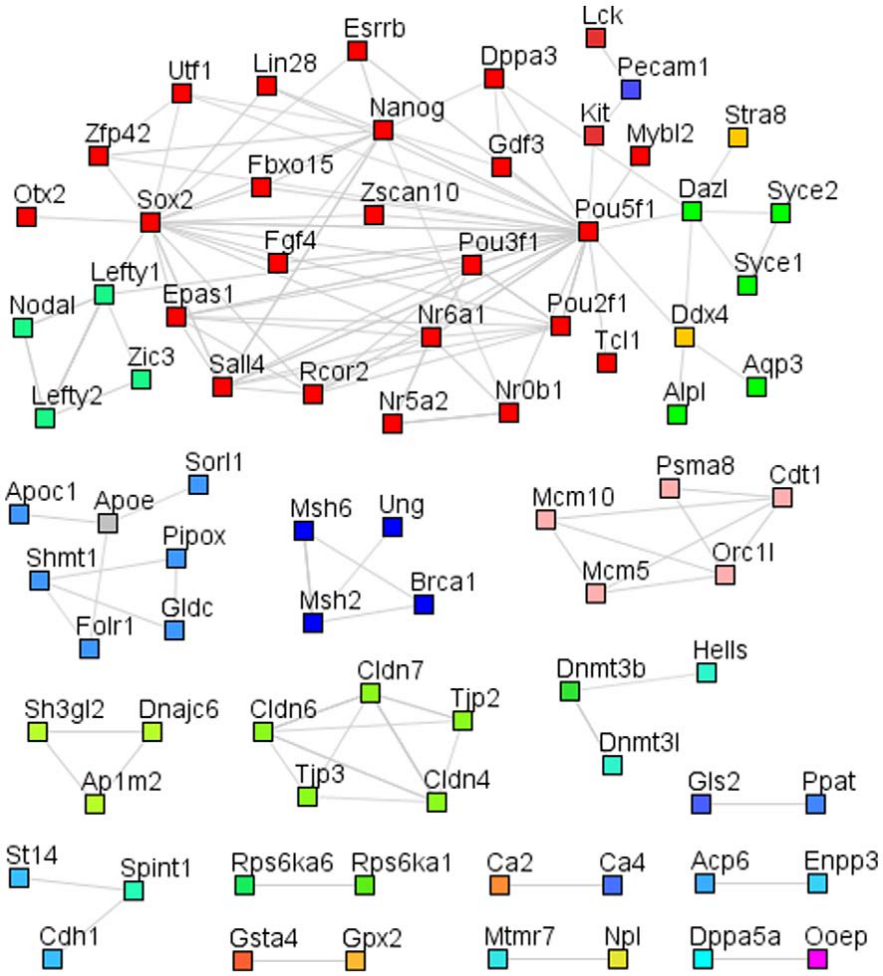
Mouse protein-protein interaction networks of genes with higher expression levels in ESCs and iPSCs compared to somatic cells. The mouse protein-protein interaction networks of genes most consistently highly expressed in ES and iPSCs, compared to the starting cell populations, have been created from the lists of biggest changes in expression, using String[71] with high confidence interactions (min score 0.7) and have been edited in Medusa[72]. They show a central, highly interconnected network of genes in which the most famous pluripotency transcription factors are to be found and which is likely to represent the core pluripotency network. They also highlight a number of genes whose functions relate to cell-cell communication, cell cycle, DNA repair and other metabolisms.

Using the genes that are significantly upregulated in ESCs and iPSCs, we have built, for both Human (Figure 2) and Mouse (Figure 3), an interaction network that represents the core pluripotency network and includes genes involved in developmental processes, stem cell maintenance and transcriptional regulation (see DAVID GO analysis in Figure S5). Our network shows a central and highly interconnected area, present both in the mouse and human analysis, where we can identify common pluripotency regulators, which are mainly transcription factors (Oct4, Sox2, Nanog, Lin28, Sall4, Otx2, Zfp42, Zic3 and Nr6a1) and TGFβ/activin/nodal signaling components (Lefty1, lefty2 and Nodal). Interestingly, most of these genes are bound in ESCs by two or more pluripotency and/or reprogramming factors and are mostly not bound by the polycomb group (see Figure S6 and supplementary File S3). Moreover, they possess high levels of H3K4 trimethylation at their regulatory regions in ESCs compared to fibroblasts (see Figure S6). While our network includes several factors that have been used successfully for reprogramming, it also includes other potential factors that might contribute to this process and may warrant further investigation.

It is also worth noting that genes involved in different functions and pathways are consistently more highly expressed in ESCs and iPSCs than in fibroblasts, such as those related to tight junction (Cldn4, Cldn7, Cdh1 and Jam2), amino acid (Gldc, Shmt1 and Gsta4) and lipid (Apoc1 and Apoe) metabolism, chromatin remodeling (Rcor2 and Hmgb2), DNA repair and stress (Brca1 and Gsta4), DNA methylation (Dnmt3b and Dnmt3l) and cell cycle regulation (Mybl2 and Utf1). However, these “secondary” functions seem to be less tightly regulated, as is reflected by the lower percentage of experiments in which these genes show higher expression in ESCs and iPSCs compared to fibroblasts, among the top 1,000 changes (see Figure S7).

Among the genes that show lower expression in ESCs and iPSCs compared to fibroblasts, we see a number of fibroblast-specific genes involved in extracellular matrix metabolism, cell adhesion, cytoskeleton organization, signaling pathways and differentiation related transcription factors both in mouse and human (see supplementary information for a more specific description). Overall, it seems consistent that the reprogramming process involves the erasing of a somatic cell specific expression program, and notably the silencing or at least repression of differentiation genes.

We should mention at this point that although genome wide transcriptional profiles give interesting clues about the events taking place in reprogramming, which are necessary for the acquisition and maintenance of pluripotency and self-renewal, other regulatory biological processes such as epigenetics, alternative splicing, regulation by microRNAs, or post translational modifications[74] will also have to be taken into account.

### Disparities between ESCs and iPSCs are revealed from their genome-wide transcriptional profiles

The first observation when comparing genome-wide transcriptional profiles of iPSCs and ESCs is, as expected, the high similarity between these cells, with genome-wide correlation coefficients usually above 0.9 (1.0 meaning identical, see Figure S8). However, differences do exist between iPSCs and ESCs transcriptional profiles and it is important to investigate whether they are relevant to iPSC biology. At a first glance, we noted that even within the core pluripotency network, not all genes were expressed at the same levels in ESCs and iPSCs, or at least not consistently in all experiments, as is highlighted in Figure S7 for the mouse network. Interestingly, when factors among the core pluripotency network show differences, they are usually expressed at a lower level in iPSCs than in ESCs, suggesting that iPSCs did not reach the full pluripotency-associated transcriptome. This has been shown to some extent by Gosh et al. [48] and more recently confirmed by Polo et al. [75].These differences, though sometimes subtle, might have functional consequences, as shown for an imprinted locus in mouse cells by Stadtfeld et al. [50]. For example, within the pluripotency network the levels of factors such as Oct4 or Sox2 have to be tightly regulated in order to maintain the balance between self-renewal and differentiation. This complex and tight regulation is also reflected by the large number of transcription factors known to be involved in pluripotency and/or self-renewal, occupying the promoters of the genes in this central network in ESCs (see Figure S6).

In 2009, Chin *et al*. also concentrated on the differences apparent between ESC and iPSC expression profiles and proposed, after comparing four different reprogramming experiments, that iPSCs are distinguishable from ESCs as they have a discrete (and conserved among experiments) gene expression signature usually reflecting insufficient induction of "ESC genes" and suppression of "fibroblasts genes" [45]. However, no functional pattern can be predicted from this signature nor can the consequences of those differences. We reproduced this analysis and extended it to more datasets, considering genes that show a minimum fold change of 1.5 and pvalue of 0.05 as differentially expressed between ESCs and iPSCs. In agreement with Chin *et al*, we identified a number of genes oftentimes differentially expressed between ESCs and iPSCs (see Figure S9). A majority of the genes that are up-regulated in ESCs compared to fibroblasts (“ESC genes”) are expressed lower in iPSCs than in ESCs. Accordingly, a majority of the genes that are down-regulated in ESCs compared to fibroblasts (“fibroblasts genes”) are higher expressed in iPSCs than in ESCs. Indeed this expression pattern might suggest that iPSCs are not fully reprogrammed and are keeping a memory of the cell type of origin. This might disturb their self-renewal and/or pluripotency competency if the level of these genes matters in pluripotent cells. However, the number of common genes that are differentially expressed in ESCs and iPSCs is reduced as more datasets are overlapped (see Figure S10), and these genes are not always consistently either lower or higher expressed among all compared iPSCs and ESCs. Hence, the number of genes showing a significant difference in gene expression between ESCs and iPSCs in most tested comparisons is low. Similar conclusions have been drawned very recently suggesting that the differences observed between ESC and iPSC are not conserved, and rather a reflection of a laboratory-bias (which may represent cell of origin, cell culture, reprogramming method…) [46], [47], although Chin et al. could confirm their results and further suggest that the type of reprogramming influences the extent of differences between ES and iPS cells [76]. The differences between ES and iPS cells we observe to not seem to be biased towards the cell type of origin, but rather seem to represent a tolerance for the expression of some somatic genes in pluripotent cells. Unfortunately, the multiplicity of methods and hands used for reprogramming certainly contributes to blurring any systematic bias and no definitive conclusion can be drawn concerning cell memory in this setup. A more controlled experiment should give an indication to whether a specific memory exists for the cell of origin. Such experiments have started to be performed, which in fact do hint at the existence of some cell memory [75].

### Monitoring the expression level of bivalent domain-containing genes could be used to screen for the best reprogrammed iPSC lines

**Figure 4:**
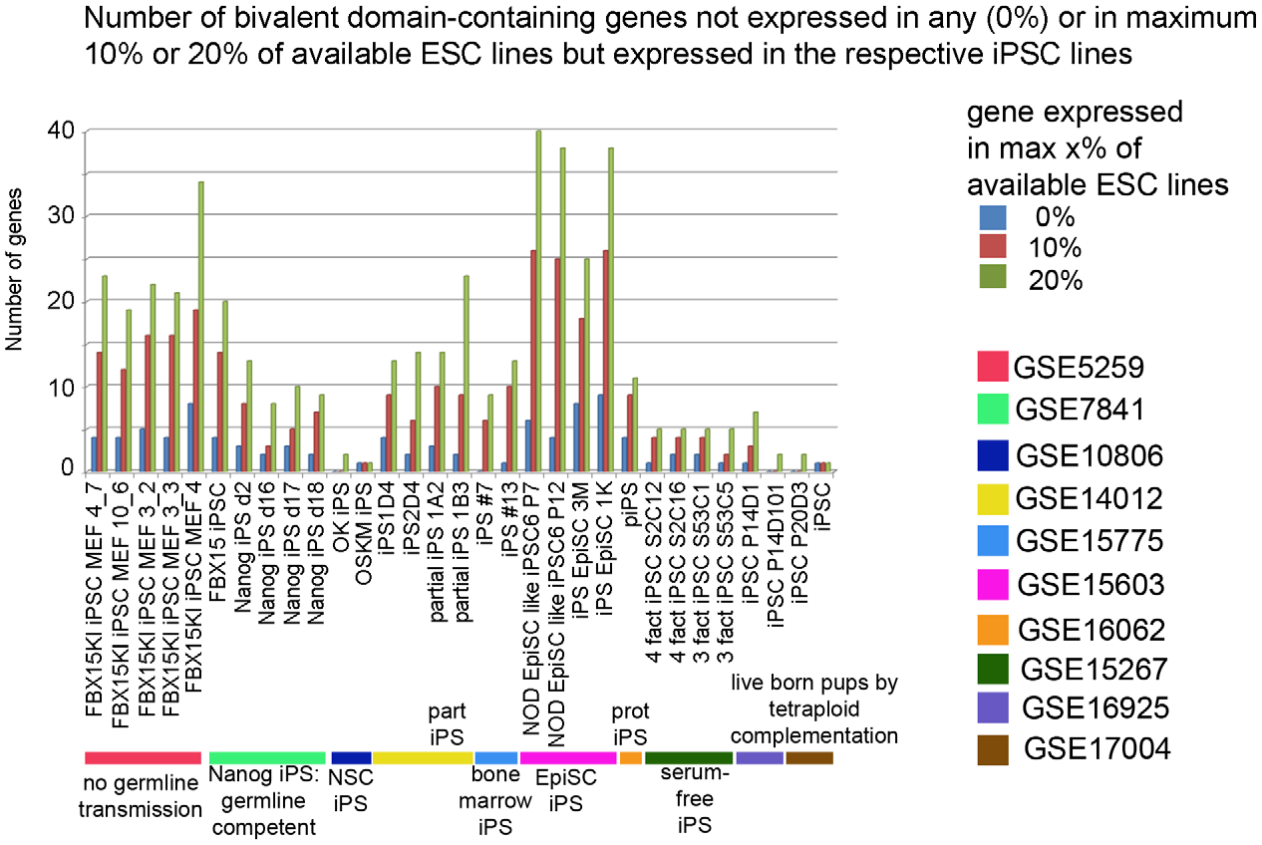
Number of genes which may be problematic for further differentiation of mouse iPSC lines generated by different laboratories. Number of bivalent domain-containing genes for each iPS cell line which show some expression in the iPS cell whereas they are silent in 100% or at least 80% of available ESC lines analyzed, and therefore could influence the differentiation potential of the iPS cell lines.

Although the differences in gene expression observed between ESCs and iPSCs do not seem to be directly affecting self-renewal or pluripotency, we hypothesized that they could affect the differentiation potential of iPSCs, a property that has not been exhaustively tested yet and will be a major concern for future applications. In order to evaluate this hypothesis, we compared the expression levels of bivalent-domain containing genes between human ESCs and iPSCs. We would like to point out that we think that the concept of cell memory and conserved differences in gene expression between ES and iPS cells are important, and have been addressed in a number of studies[45], [46], [47], [75], [76]. We believe however that reprogramming may take different paths (which we are not able to infer as we merely have snapshots of the starting and end point) to achieve the pluripotent state, and hence leave different "scars", which albeit not consistent may have functional consequences. Therefore, we decided to focus on each individual iPSC line for the analysis of bivalent genes. Hence, for each human and mouse dataset we studied the expression of genes carrying bivalent domains in ESCs that were obtained from the overlap of three ChIP-on-chip studies in human [65], [66], [67] or in mouse [60], [65], [77] (Figure S2). We examined the correlation coefficients of the genome-wide transcriptional profiles of ESCs and iPSCs and the profiles of genes marked with bivalent domains (see Table 1 and Figure S3). Since the characterization of the published iPSC lines are usually more thorough in mouse, we decided to test our hypothesis on the mouse iPSC lines. Although the correlation coefficients are generally high for genome-wide as well as for the set of bivalent domain-contaning genes, we see some striking differences among reprogramming experiments. It is noteworthy that the bivalent genes profiles of the iPSC lines described to contribute to viable mice through tetraploid complementation assay (the most stringent proof of pluripotency available so far, GSE16925 and GSE17004) have the highest correlation coefficients when compared with the ESC lines. As expected, the correlation between bivalent genes profiles of fibroblasts and ESCs is very low and especially much lower than the one obtained from the comparison of genome-wide profiles. Moreover, the correlation between partially reprogrammed cells [52], [54], which can self-renew but have not reached pluripotency, and ESCs is much lower when comparing the expression of genes marked with bivalent domains, supporting the idea that this correlation is a good indicator of the quality of the reprogrammed cells. As mentioned earlier, bivalent domain marked genes are usually silenced or expressed at low levels in ESCs. However, in our analysis we found a number of the genes described to have both H3K4 and H3K27 methylation marks in ESCs that are significantly expressed in most ESC lines analyzed. This could reflect the presence of a heterogeneous population of ESCs [43], the presence of a number of differentiating or differentiated cells in the ESC culture or that the coocurrence of other undescribed chromatin marks at the regulatory regions of these genes renders them transcriptionally active. Thus, in agreement with the model of bivalent domain containing genes we rely on, we concentrated on the bivalent domain containing genes that are silent in at least 80% of the mESC lines for which we had available microarray data, and among them we identified those expressed in each individual iPSC line as "potentially problematic genes" since their silencing in a pluripotent cell population is supposedly required. We believe that these genes are the ones whose expression in iPSCs could restrict or at least bias the differentiation potential. Encouragingly, the iPSC lines that were shown to generate viable mice by tetraploid complementation assays (GSE16925 and GSE17004) express none to very few of such genes, whereas the first iPSCs generated that did not contribute to the germline (GSE5259), as well as the partially reprogrammed iPSC lines (GSE14012), express a number of these potentially troublesome genes (Figure 4). For example, the partially reprogrammed iPSC lines 1A2 and 1B3 (GSE14012), as well as the Fbx15KO iPSC line, which showed a limited potency (GSE5259), express Hoxc8, which is a homeodomain gene important for early embryogenesis, especially for neural development, and whose expression level is normally tightly regulated [78] and quasi-inexistent in the ESCs used in our study. The expression of this developmental gene might explain or at least reflect the limited potency of these lines. We extracted a list of potentially problematic genes for human based on similar criteria, and being a little bit more conservative, i.e. only genes which are expressed in a maximum of 1 ESC line present in our study (see supplementary File S4). The number of "potentially problematic genes" for some of the human iPSC lines is depicted in Figure S11. Interestingly, the two studies using OCT4, SOX2, LIN28 and NANOG as the reprogramming cocktail (from normal and SMA patient fibroblasts) show a reduced number of "potentially problematic genes." A little bit more concerning is the rather large number of "potentially problematic genes" expressed in some of our KiPS lines, which passed all standard criteria for pluripotency tests available in human. This again raises the question of the possible lack of stringency of pluripotency tests in human, and also highlights the differences observed from 2 lines obtained in similar conditions, and the fact that each line, to a certain extent, probably follows its own path of reprogramming.

**Table 1:** Comparison of human reprogramming experiments with a focus on ES/iPS comparison.

	Maherali, N. *et al.*, *Cell Stem Cell* 3 (3), 340–345 (2008).	Huangfu, D. *et al Nat Biotechnol* 26 (11), 1269–1275 (2008).	Lowry, W.E. et al., PNAS 105 (8), 2883–2888 (2008).	Aasen, T. *et al.*, *Nat Biotechnol* 26 (11), 1276–1284 (2008).	Zhao, Y. *et al.*, *Cell Stem Cell* 3 (5), 475–479 (2008).	Ebert, A.D. *et al.*, *Nature* 457 (7227), 277–280 (2008).	Yu, J. et al., Science 324 (5928), 797–801 (2009).	Soldner, F. et al., Cell 136 (5), 964–977 (2009).
Starting cell type	Neonatal foreskin fibroblasts and fibroblasts differentiated from iPS	Primary fibroblasts: BJ and NHDF	Neonatal foreskin fibroblasts	Foreskin keratinocytes	Adult foreskin fibroblasts, fetus skin fibroblasts	Fibroblasts from a type 1 SMA patient and his unaffected mother	Foreskin fibroblasts	Parkinson disease patients dermal fibroblasts
Factors used	- Oct4, Sox2, c-Myc, Klf4- Oct4, Sox2, c-Myc, Klf4, Nanog	Oct4, Sox2, Klf4, Valproic acid (VPA)	Oct4, Sox2, c-Myc, Klf4, Nanog	Oct4, Sox2, c-Myc, Klf4	Oct4, Sox2,Klf4, c-Myc, Utf1, P53si	Oct4, Sox2, Lin28, Nanog	Oct4, Sox2, Nanog, Lin28, c-Myc, Klf4, SV40LT	Oct4, Sox2, c-Myc, Klf4
Vector used	DOX-inducible lentivirus	Retrovirus	Retrovirus	Retrovirus	Lentivirus	Lentivirus	oriP/EBNA1-based episomal vector with IRES2	Excisable DOX-indusible lentivirus
Platform	Affy HG-U133 plus 2.0 (GPL570)	Illumina Human Ref-8	Affy HG-U133 plus 2.0 (GPL570)	Affy HG-U133 plus 2.0 (GPL570)	Phalanx Human one aray (GPL6254)	Affy HG-U133 plus 2.0 (GPL570)	Affy HG-U133 plus 2.0 (GPL570)	Affy HG-U133 plus 2.0 (GPL570)
GEO accession number	GSE12390	Pers. Comm	GSE9865	GSE12583	GSE12922	GSE13828	GSE15148	GSE14711
Corr coeff whole array iPS/ES: average (min-max)	Primary iPS: 0.988 (0.984–0.989) Secondary iPS: 0.991 (0.990–0.991)	0.970 (0.950–0.980)	Part-iPS: 0.946 (0.928–0.962)iPS: 0.964 (0.953–0.976)	0.956 (0.950–0.964)	Pre-iPS: 0.728 (0.730–0.812)iPS: 0.887 (0.832–0.923)	0.973 (0.967–0.979)	0.970 (0.960–0.978)	Integrated: 0.969 (0.965–0.972)Excised: 0.972 (0.969–0.977)
Corr coeff 316 biv domain genes	Primary iPS: 0.961 Secondary iPS: 0.970	0.974	Part-iPS: 0.814 iPS: 0.885	0.949	Pre-iPS: 0.497 iPS: 0.881	0.985	0.979	Integrated: 0.973 Excised: 0.970
Some of the most statistically significant different genes between ES and iPS cells	Primary iPS: GAS1, FGFR4, MSX2 Secondary iPS: RAD51, LEFTY2, MSX2	GSTM2, DNAJC15, CTNNB1	iPS: IAH1, RELL2, GNG3	NPM1, RPL29, NDUFB2	iPS: ROCK1, EPS15, CITED2	SLITRK4, ZFP208, CR1	GLIPR1, SOX11, ELAVL1	Integrated: PLCL2, LEFTY2Excised: MEG3, ZNF273

Summary of the datasets and the reprogramming experiment used for the comparison of ESCs and iPSCs. For each dataset, the correlation coefficient on the percentranks for the genome-wide profile (average and, in parenthesis, minimum and maximum correlation between ES and iPS samples hybridized) is given, as well as for the profile of 316 bivalent-domain containing genes, which reflects more stringently the functional equivalence in terms of the differentiation potential between ESCs and iPSCs. Some of the most significantly differentially expressed genes between ESCs and iPSCs for each dataset are also shown.

Although it has become increasingly clear that ESCs and iPSCs exhibit differences, systematic biases are hard to highlight (and thereby biological significance of the differences hard to assess). This could also be explained by the fact that not only might ESCs and iPSCs be at different pluripotent states, but that there is also more than one iPSC state, and reprogramming can possibly take different routes to achieve pluripotency and self-renewal [43]. This unfortunately also means that each iPSC line which will be utilized in further experiments, and especially for those for therapeutical purposes, should be extensively characterized. We believe that checking the silencing of "potentially problematic bivalent domain genes," (i.e. the genes that have bivalent domains in ESCs and are normally silenced in ESCs) in the generated iPSC lines can give a good indication of the quality of each line, thus helping to select the most promising iPSCs for further characterization.

### Reprogramming barriers and safety of iPSCs

Tumorigenesis is currently one of the major concerns in the pluripotent stem cells and regenerative medicine fields. The first clinical trial using hESC-derived oligodendrocyte progenitor cells for spinal cord injured patients has been stalled due to the development of cysts in more than 50% of the patients [69]. Moreover, a recent report highlights that several mouse iPSC lines displayed even higher rates of tumor formation when implanted into recipient mice compared to ESCs [79]. It is likely that understanding the molecular pathways controlling the transition to pluripotency will uncover the potential risks which will affect the clinical use of iPSCs and inspire strategies to overcome them.

**Figure 5:**
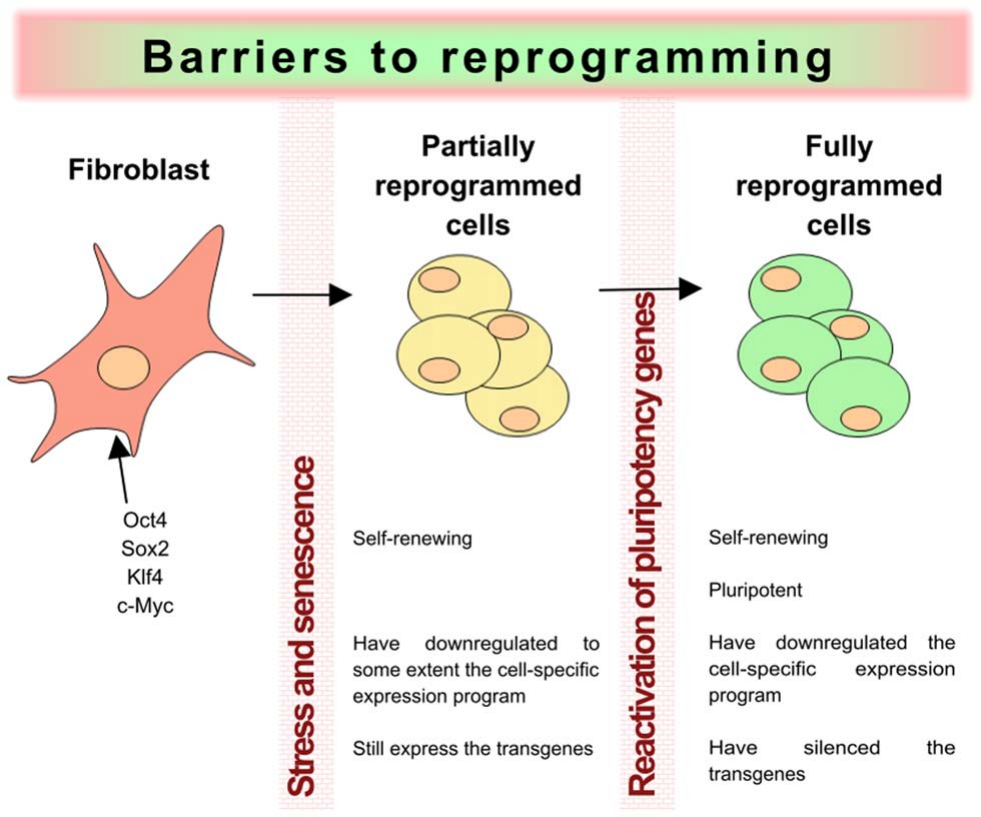
Barriers to reprogramming. The process of somatic cell reprogramming entails overcoming the cellular barriers that preserve cell identity. The first barrier consists of the stress generated by the overexpression of factors that stimulates apoptosis and reduces cell viability. The p53 pathway is an important factor for this barrier. Many cells that overcome this barrier end up trapped in a partially reprogrammed state in which they are able to self-renew but are not yet pluripotent, as reflected by their ability to form tumors when injected into immunosuppressed mice. These cells are dependent on the presence of the transfactors and cannot activate the expression of the endogenous pluripotency factors due to the presence of a non-permissive chromatin environment on their regulatory regions, constituting a second barrier to reprogramming. Only after overcoming this barrier are cells fully pluripotent and able to produce teratomas after injection into immunodepressed mice.

Reprogramming must indeed circumvent the mechanisms that normal adult somatic cells have developed to preserve cell identity, ensure their functionality and protect them against viral infections, cell damage and transformation. The first barrier that the cells face during reprogramming consists of overcoming the initial stress generated by the over-expression of transcription factors, which is likely to activate mechanisms such as apoptosis, senescence and decreased cell viability (see Figure 5). Interestingly, recent studies identified the p53 pathway as a barrier against reprogramming [52], [61], [80], [81], [82], [83]. These observations raise the question of whether rare cells that are deficient in this very important pathway could be positively selected during the reprogramming process, increasing the probability of accumulating mutations and genetic aberrations, which would clearly increase their potential tumorigenesis risk.

Once the first barrier is passed, most of the cells end up trapped in a partially reprogrammed state in which they have acquired self-renewal capabilities and have, to some extent, down-regulated the differentiation-specific transcription patterns, but yet have failed to overcome the epigenetic barrier towards the activation of the endogenous pluripotency genes and are thus non-pluripotent [35]. Interestingly, these partially reprogrammed cells are very similar to transformed cells in regards to their ability to grow indefinitely in a relative undifferentiated state. Overcoming the second reprogramming barrier leads to the reactivation of endogenous pluripotency genes, which was hindered by the presence of a repressive chromatin environment around their regulatory regions. Activation of the endogenous pluripotency network is likely to mediate the silencing of developmental genes through the establishment of bivalent marks at the regulatory regions of these genes. We suggest with our analysis of bivalent domain-containing genes that their expression in iPSCs, implying the aberrance or lack of establishment of epigenetic marks during reprogramming, might give rise to cells with defective silencing of some differentiation genes. In accordance with this proposal, a recent report correlates the quality of human iPSC with the acquirement of proper bivalent marks at differentiation genes [70]. Moreover, the genome wide analysis of DNA methylation at CpG sites in ESC and iPSC indicates that at certain loci iPSCs remain incompletely or aberrantly reprogrammed, and those are especially enriched at genes involved in developmental processes [71]. Thus, whenever the process of reprogramming-mediated silencing fails, aberrant expression of developmental genes may occur, affecting the differentiation potential of the cells. It could increase the probability that cells cannot answer to the differentiation cues supplied to them and that partially undifferentiated cells could remain after transplantation, which might be at the roots of tumorigenesis that may impede potential future clinical applications [72].

In summary, it is possible that the process of reprogramming promotes the positive selection of cells in which the mechanisms of cell identity preservation are not fully functional either because mutations or the establishment of aberrant epigenetic marks during reprogramming confers on them a growth advantage compared to the rest of the population. Either event could render iPSCs more prone to tumorigenesis and/or show an aberrant differentiation potential.

Hence, the origin and genetic and/or epigenetic history of the cells used for reprogramming surely play a determining role in the safety of iPSCs. The origin of the cells has already been shown by Miura et al. to influence the risk of tumor formation [72]. This risk seems to be dependent on the ability of the cells to differentiate, and hence on the presence of a few remaining undifferentiated cells in the transplant. Moreover, the in vitro manipulation and expansion of cells is likely to cause stress and this should probably also be taken into account. Following these guidelines, cord blood stem cells, young and easily obtainable, may represent an ideal candidate [78], [79] when looking for the most suitable cell type to start with.

In order to gauge more thoroughly the risks involved in the possible usage of iPSC cells for therapy, we also compared the expression levels of oncogenes and tumor suppressor genes between iPSCs and ESCs and highlight in Figure S12 changes that may render iPSCs at higher risk of tumorigenesis than ESCs.

### Conclusions and outlook

After analyzing, in detail, genome-wide transcriptional profiles of starting cell populations, partially reprogrammed cells and iPSCs and comparing these with ESCs, we conclude that iPSCs and ESCs share a well-defined core pluripotency network. However, some core genes often seem expressed at lower levels in iPSCs. In addition, this network comprises not only the usual pluripotency transcription factors, but also genes not yet described as, but likely to be, involved in pluripotency and/or self-renewal and genes responsible for many other biological processes, such as cell-cell communication and metabolism.

When analyzing the differentially expressed genes between ESCs and iPSCs for each available experiment with human cells, we see that differences are not systematic and most likely do not reflect the memory of the cell type used for reprogramming. Moreover, differences are found when comparing the expression of critical developmental regulators (marked with bivalent domains in ESCs), suggesting that the differentiation potential of iPSCs could be different than that of ESCs.

Although we cannot answer the question of whether iPSCs are truly functionally equivalent to ESCs, it seems increasingly obvious that there exists more than one state of pluripotency. This would explain why we can distinguish between ESCs and iPSCs, but also between iPSCs generated with different protocols. As we believe it is important to select the best iPSCs in terms of their differentiation potential, we propose that checking the newly generated iPSC lines for the silencing of a number of genes marked with bivalent domains would assist in preselecting the most promising iPSCs for further studies. Importantly, even though the field of somatic cell reprogramming moves incredible fast and brings us closer every day to getting the "perfect" protocol for iPSC generation in terms of efficiency, a crucial question remains: will we be able to get cells which are safe to use for therapeutical applications? To answer this question, not only will different cell types, ages and origins have to be tested, but also the protocol used for the generation of the iPSCs. Moreover, understanding the path through which somatic cells arrive to a pluripotent state should allow us to evaluate, more accurately, the potential risks inherent in the use of iPSCs in therapy. The propensity of iPSCs to differentiate and not to go wayward after transplantation, judged by the integrity of their genome and epigenome, will need to be evaluated in great detail.

## Supporting Information

Text S1 Detailed description of up and down mouse networks. Functional description and relevance of genes present in the networks of genes higher or lower expressed in pluripotent cells than in MEFs.
(0.31 MB PDF)

Figure S1 Schematic representation of the strategy used to reconstruct human and mouse networks of genes that are either up-regulated or down-regulated in reprogramming.
(0.23 MB PDF)

Figure S2 In order to evaluate the expression of genes containing bivalent domains, and thereby the quality of the reprogrammed iPSCs compared to ESCs, we have used the overlapping set of genes from 3 genome-wide characterizations of bivalent domain containing genes (see figures below for the human and mouse datasets) which we consider being a high confidence set.
(0.10 MB PDF)

Figure S3 Correlation coefficients of different samples and experiments on the profiles of 316 bivalent-domain-containing genes in Human. It is important to note that the profile hasn't been always done on the same platform, which explains why the correlation inter-experiments is sometimes not good.
(0.14 MB PDF)

Figure S4 Principal component analyses of Human and Mouse reprogramming datasets show that ESCs and iPSCs usually cluster together, far from the starting somatic cell population. Principal component analysis of genome-wide intensity values (or log of intensity gcrma-normalized) (A) Human datasets (B) Mouse datasets.
(0.72 MB PDF)

Figure S5 Functional analysis of genes up- and downregulated in Human and Mouse ESCs and iPSCs in comparison to somatic cells using DAVID. Gene Ontology annotations of biological process, molecular function and cellular component as well as genes enrichment in KEGG pathways for the following comparisons: (A) genes upregulated in Human ESCs and iPSCs; (B) genes downregulated in Human ESCs and iPSCs; (c) genes upregulated in mouse ESCs and iPSCs; (d) genes downregulated in ESCs and iPSCs.
(0.21 MB PDF)

Figure S6 Mouse network of genes upregulated in both ESCs and iPSCs compared to MEF - Overlap with transcription factors binding and chromatin marks in ESCs.
(1.01 MB PDF)

Figure S7 Level of reproducibility of the observation of a gene expression change between MEF and iPSCs or ESCs within the most significant changes in each studied dataset. Highlight of the level of reproducibility of the presence of genes in the mouse network of most significantly upregulated genes in ESCs and iPSCs compared to MEF in all available comparisons between (A) ESCs to MEF (B) iPSCs to MEF.
(0.17 MB PDF)

Figure S8 Analysis of the differences between ESCs and iPSCs transcriptional profiles with a focus on bivalent-domain containing genes in mouse and human datasets. Correlation coefficients for genome-wide profiles and profile of genes containing bivalent domains in ESCs for (A) Human (B) Mouse.
(0.16 MB PDF)

Figure S9 Overlap of genes showing significantly different levels between iPSCs and ESCs in each dataset for Human and Mouse with the direction of the change. The expression change direction between fibroblasts and ES cells for genes which show differences between ES and iPS cells confirms that a majority of genes differently expressed between ES and iPS cells are "ES genes" which are lower expressed in iPS than in ES cells. Blue columns represent genes whose expression level is lower in ES cells than in fibroblasts, while red columns represent genes whose expression level is higher in ES cells than in fibroblasts.
(0.18 MB PDF)

Figure S10 Gene expression signature of iPSCs: reanalysis of human and mouse datasets with the Chin et al. method. The genome-wide gene expression profiles of iPSC and ESC lines were compared for human (in total 8 pairwise comparisons) and mouse (in total 15 pairwise comparisons). Genes showing a minimum fold change of 1.5 and pvalue lower than 0.05 were identified as significantly differently expressed between ESCs and iPSCs. The number of comparisons of human (or mouse) iPSCs and ESCs is represented on the X axes. The number of genes that are differently expressed between human (or mouse) iPSCs and ESCs in at least one comparison, as well as the overlap of two (or more) comparisons, is represented on the Y axis.
(0.09 MB PDF)

Figure S11 Number of potentially problematic bivalent domain-containing genes expressed in different human iPSC lines. The iPSCs (different lines, different clones or different passages of the same line) from the available human datasets are represented on the X axis. For each iPSC, the number of bivalent domain-containing genes expressed in the given iPSC whereas silent in 100% (blue) or in at least 90% (red) of the human ESC lines analyzed, is represented on the Y axes.
(0.20 MB PDF)

Figure S12 List of tumor suppressor genes down-regulated and oncogenes upregulated in human iPSCs compared to ESCs. List of tumor suppressor genes and oncogenes whose expression levels renders iPSCs suspicious.
(0.04 MB PDF)

File S1 Paper list. List of papers reporting reprogramming experiments from human and mouse cells with their citation and summarized message.
(0.10 MB XLS)

File S2 Top 1000 changes human. Summary of genes among the top 1000 changes in any human pairwise comparison with details of presence among the top 1000 and rank in all comparisons and highlighting similarities and differences between ES and iPS cells.
(2.74 MB XLS)

File S3 Summary genes in up network mouse. Summary of the genes present in the network of genes higher expressed in pluripotent cells than in MEFs, with annotation for chromatin marks (H3K4me3 and H3K27me3) in MEFs and ESCs, as well as binding by an array of transcription factors, among which the usual reprogramming factors.
(0.08 MB XLS)

File S4 Potentially problematic genes. Summary of genes marked with bivalent domains in ESCs, which are normally silenced in ESCs and expressed in at least one iPSC line for human and mouse, annotated with their phenotype in mouse, and for which the expression level (as percentrank) is shown when higher than 40% (considered actively expressed).
(0.34 MB XLS)
