# Supplementary material for: Analysis of Human and Mouse Reprogramming of Somatic Cells to Induced Pluripotent Stem Cells. What Is in the Plate?
Source: PLoS One. 2010 Sep 17;5(9):e12664. doi: 10.1371/journal.pone.0012664 (PMC2941458; doi:10.1371/journal.pone.0012664)
Supplement: Text S1 — Detailed description of up and down mouse networks. Functional description and relevance of genes present in the networks of genes higher or lower expressed in pluripotent cells than in MEFs. (0.31 MB PDF) [file pone.0012664.s001.pdf]

Functional description of the genes higher expressed in ES and iPS cells compared to fibroblasts in mouse

The prevention of differentiation and the promotion of proliferation are necessary to maintain the pluripotency of ES cells during their self-renewal [1]. Genes associated with the specific features of ES cells, included or not in our network of upregulated genes in ES and iPS cells (Figure 3 in the main text, Figures S3 and S4) are described below.

Transcription factors involved in the regulation of pluripotency

Transcription factors associated with the regulation of pluripotency of ES cells by either the suppression of differentiation paths or the promotion of pluripotency are components of our network.

Oct4, Sox2, and Nanog are three major hubs of our network and key regulators of the transcriptional network that controls self-renewal and pluripotency of ES cells [1]. Oct4 and Sox2 are present in most reprogramming cocktails to reprogram the somatic cells into iPS cells. Nanog encodes a homeodomain-containing transcription factor (TF). The deletion of Nanog induces differentiation of ES cells into primitive endoderm, while its overexpression can maintain mouse ES cells in a pluripotent state in the absence of leukemia inhibitory factor (LIF) [2]. Oct4 is a POU-domain TF. The knockdown of Oct4 promotes the expression of endodermal and trophoblast differentiation markers [3] whereas Oct4 overexpression results in a mixed population of differentiated cells expressing markers of endoderm and mesoderm [4]. A tight regulation of the level of Oct4 expression is hence very important for the maintenance of pluripotency. Sox2 is a member of HMG-domain family of transcription factors. Sox2 knockdown in mouse ES cells induces differentiation into multiple lineages, including trophectoderm [1]. Sox2 cooperates with Oct4 to activate Oct4 target genes [1], and it also has been shown to regulate Oct4 expression to maintain its optimal levels required for the pluripotency of ES cells [5]. Oct4, Sox2 and Nanog regulate each other, many other genes in a cooperative fashion and also themselves [1].

Zic3, a member of the Gli family of zinc finger transcription factors, is a downstream target of Oct4, Nanog and Sox2. It has been suggested that Zic3 supports pluripotency by preventing endodermal lineage differentiation, possibly through Nanog-regulated pathways [6].

Nr0b1 and Nr5a2 are orphan nuclear receptors working to maintain the pluripotency of ES cells. Nr5a2 (Lrh1) activates Oct4 expression by binding to the SF-1 response elements in the proximal promoter and proximal enhancer of Oct4. Knockout of Lrh1 results in loss of Oct4 expression at the epiblast stage and early embryonic death [7]. The expression of Nr0b1 (Dax1) is regulated by Oct4 and Stat3 [8]. It has been shown that the knockdown of Dax1 by RNA interference induced differentiation of ES cells towards endoderm [9].

By contrast, Nr6a1 (Gcnf) is a nuclear receptor that represses ES cells phenotype during differentiation [10]. Nr6a1 controls pluripotency by directly repressing the expression of Oct4 and Nanog. In addition Sox2 and Fgf4 are indirectly regulated through Oct4. Although this might appear contradictory, the balance between positive and negative regulators of Oct4 expression is necessary since its level has to be tightly regulated to maintain pluripotency [4].

Sall4, a zinc-finger TF, activates expression of Oct4 [11], and its knockdown influences the expression levels of most commonly used reprogramming genes (Oct4, Sox2, c-Myc and Klf-4) [12]. In addition, Sall4 was reported to bind many genes that are partly regulated by chromatin-based epigenetic events and therefore has been suggested to regulate pluripotency through integration of transcriptional and epigenetic controls [12].

Esrrb belongs to the family of orphan nuclear receptors. Esrrb is known to positively regulate Oct4 and Nanog expression, and its overexpression alone is able to maintain self-renewal and pluripotency of mouse ES cells [13]. Esrrb knockout embryos die around E10.5 due to the impaired placental formation and abnormal chorion development [14].

Zscan10 (Zfp206) – Zinc finger and SCAN domain containing gene – has been reported as a pluripotency-related TF [15], [16]. Zscan10 is directly regulated by Oct4 and Sox2 [15], and the knockdown of this gene increases the proportion of differentiated cells in vitro [16].

Zfp42 encodes a C2H2 zinc-finger protein and is highly expressed in undifferentiated mouse and human ES cells. It has been reported that a RNAi knockdown of Zfp42 in mouse ES cells induced ES cells differentiation to endoderm and mesoderm lineages [17]. However, recent results show that in the absence of Zfp42 mouse ES cells remain pluripotent and contribute whole embryos after blastocyst injection [18].

Genes involved in cell cycle and proliferation

ES cells have an unusual cell cycle structure, characterized by a high proportion of cells in S-phase and a short G1 phase, which can explain the rapid cell division of ES cells comparing to differentiated cells that spend most of their time in G1 [19]. In mouse ES cells the G1 phase control pathways are reduced or absent, a specificity which is also associated with the deregulated proliferation of tumor cells [20].

Mybl2 is a TF involved in the control of cell cycle progression and is essential to all proliferating cells. In the absence of this gene, embryos die just after the implantation due to the inner cell mass defect, generally attributed to a proliferation defect in the cell cycle phase of G1. Mybl2 has been also shown to directly regulate Oct4 expression in mouse ES cells [21].

Lin28a is a cytoplasmic protein with a unique pairing of RNA-binding motifs. Its specific downregulation results in decreased cell proliferation. It has been suggested that Lin28a might regulate transcription of genes important for cell growth (Cdk4, cyclins A and B), whose expression was also reduced upon downregulation of Lin28a [22].

Utf1 is a TF activated by Oct4 and has been shown to contribute to the proliferation of ES cells, possibly by promoting cell cycle progression. Downregulation of Utf1 results in decreased proliferation of ES cells, as well as decreased teratoma formation in vivo [23].

Genes involved in signaling pathways

Nodal, Lefty1 and Lefty2 are members of the TGF-β superfamily, and function to maintain TGF-β/activin/Nodal signaling. Acvr2b encodes a receptor that modulates signals for ligands belonging to TGF-β superfamily. This pathway has been shown to be important for the proliferative capacity of ES cells as the inhibition of TGF-β/activin/Nodal signaling by Smad7 or the specific inhibitor SB-431542 resulted in decreased proliferation of mouse ES cells without decreasing their pluripotency [24].

Gdf3 is also a member of the TGF-β superfamily (as Lefty1, Lefty2 and Nodal) and acts as a BMP signaling inhibitor. It has been reported to have a supportive role in

maintaining pluripotency in mouse ES cells and their ability to differentiate into different cell types. Interestingly, in human ES cells, higher levels of Gdf3 comparing to mouse ES cells are needed to maintain the expression of pluripotency markers. It has been claimed that this is due to the difference of BMP signaling between mouse and human [25].

As reviewed in [1], Eras and Tcl1 are two modulators of phosphoinositol-3-kinase (PIP3K)/Akt signaling pathway that plays an important role in proliferation and maintenance of pluripotency in ES cells. Tcl1 increases the Akt activation by forming a heterodimeric complex with Akt, while Eras activates PIP3K. Eras is not a part of the network but it is included in the list of 346 genes upregulated in ES and iPS cells.

The signaling by the cytokine tyrosine kinase receptor Kit and its ligand – stem cell factor –regulates hematopoietic stem cell (HSC) self-renewal, proliferation and differentiation in mouse and in human [26],[27]. It has been shown that Kit null mouse ES cells are able to self-renew, although Kit signaling is essential for their survival during differentiation upon LIF withdrawal, in contrast to normal differentiation of Kit null inner cell mass or epiblast cells in vivo [28].

Fibroblast growth factor Fgf4 is a major activator of Erk1/2 signaling cascade in mouse ES cells. It has been shown that differentiation of ES cells to neural or mesodermal lineages is arrested in the absence of Fgf-Erk, while the expression of pluripotency markers Oct4, Nanog and Rex1 is not alternated [29].

Self-renewal and pluripotency are very peculiar features of ES cells, but are not enough to define the biology of those cells, and other biological functions are found to be consistently upregulated in ES and iPS cells compared to fibroblasts. Those are described below.

Genes involved in DNA metabolism

The establishment of specific DNA methylation patterns is essential for the development of the early embryo. The importance of DNA methylatransferases Dnmt3a and Dnmt3b for *de novo* methylation and for mouse development has been described [30]. Both Dnmt3a and Dnmt3b are involved in CpG dinucleotide methylation, whereas their function is non-overlapping. Dnmt3b is required for methylation of centromeric minor satellite repeats. Inactivation of both genes blocks *de novo* methylation in mouse ES cells and results in early embryonic lethality. Another DNA methyltransferase, Dnmt3l has been shown to stimulate the activity of Dnmt3a and Dnmt3b [31]. Hells (Lsh), a member of the SNF2 chromatin remodeling family, has been suggested to be involved in the process of gene silencing during embryogenesis mediated by Polycomb repressive complex (PRC) [32]. The authors proposed that Lsh might perform a scaffolding-like function to promote interaction between Dnmt3 and PRC.

Rcor2 (coREST) is a chromatin regulatory protein involved in a transcriptional repression by Gfi proteins [33]. Inhibition of Rcor2, along with the histone demethylase LSD1, perturbs differentiation to hematopoietic lineage. In addition, Rcor2 is a part of the REST repression complex, which mediates suppression of neuronal genes expression [34].

Since mutation in ES cells can potentially affect multiple cell lineages, ES cells have to develop a sensitive mechanism to maintain DNA integrity. This is done either by DNA damage repair, or increased cell cycle regulation (described earlier) and removal of

damaged cells by apoptosis [35]. Although the majority of these genes have not been studied in ES cells, their function in somatic cells has been described. Brca1 belongs to the tumor suppressor gene family and is involved in DNA repair by homologous recombination. Brca1 deficiency in mouse ES cells has been shown to result in decreased homologous recombination frequencies [36]. In addition, as reviewed in [37], Brca1 is involved in a maintenance of genome integrity by regulation of centrosome duplication and G2-M checkpoint control. Homozygous Brca1 mutant embryos die, showing severe abnormalities including growth retardation, mesoderm and neural tube defects [37]. Msh2 and Msh6 are involved in the DNA mismatch repair that is essential for the stability of genome. Mice lacking either of these genes in combination with Msh3 gene had shown an increase in the mutation frequencies compared to the wild-type mice [38]. Ung functions as a major uracil glycosylase during base excision repair to eliminate uracil that was created during somatic hypermutation (SHM) or class switch recombination (CSR). Inactivation of Ung in mice results in defective CSR and in almost complete elimination of transversion mutations from C and G [39]. Cdt1 belongs to a family of replication proteins. It has been claimed to be essential for the efficient loading of minichromosome maintenance (MCM) protein complex onto template DNA [40]. The MCM complex is a DNA replication licensing system that is suggested to act as a replicative helicase in eukaryotes [41]. Mcm5 and Mcm10 are components of this complex. Orc1l is a component of the Origin recognition complex (ORC) that is involved in the initiation of DNA replication in eukaryotes along with MCM complex. Multiple interactions among ORC subunits and MCM proteins have been revealed [42].

Meiosis and germ line development-related genes in the network

Syce1 is a member of the synaptonemal complex, essential for normal progression of meiotic recombination and formation of crossover [43]. Syce1 deficient mice lack mature gametes and therefore are infertile. Syce2, another member of the synaptonemal complex, has been shown to be essential for the complex assembly [44].

Dazl encodes a RNA-binding protein and is expressed in all stages of mouse preimplantation embryo [45]. The functional role of Dazl has been evaluated in light of its contribution to spermatogenesis. In mouse testis Dazl was shown to specifically bind a subset of mRNAs necessary for germ cell development and cell cycle progression, including proteins involved in translation activation and transcription regulation [46]. In human, the presence of Dazl has been detected not only in the stem cells but also in the trophectoderm that cannot give rise to the germ cells, therefore it has been suggested that Dazl might have some role beyond the germ cells development [47]. Authors have proposed that Dazl might be a positive marker for the quality of blastocysts and might even have an influence on the implantation and survival chances of the embryo.

Ddx4 (Vasa) is specifically expressed in germ cell lineage and has a function in germ cell development. It has been found that pre-meiotic translation of Vasa is mediated by Dazl [48].

It is not clear why these meiosis-specific genes are upregulated in ES cells. A possibility is that they have another less well described function relevant to the physiology of pluripotent and self-renewing cells.

Genes involved in other metabolisms

As mentioned earlier, ES cells have a specific cell cycle that allows them to proliferate quickly. Cell cycle is a process that requires high energy associated with high metabolic activity, as metabolic perturbations delay cell cycle entry [49]. Genes that are associated with metabolism in our network are described below. We have noticed that promoters of many of these genes are bound by c-Myc, a TF that, in combination with other TFs, has been widely used to reprogram somatic cells into iPS cells. It has been shown that c-Myc regulates genes involved in metabolism and cell cycle progression, in particular, genes that function in glycolysis, oxidative phosphorylation, and mitochondrial biogenesis [49].

Promoters of the following genes are bound by c-Myc: Gldc, Shmt1, Gsta4, Apoc1, and Gls2.

Gldc (glycine decarboxylase) is a component of a glycine cleavage system (GCS) - a mitochondrial enzyme complex that degrades glycine. An inherited deficiency of Gldc causes an inborn error of metabolism, glycine encephalopathy (GE), characterized by neonatal coma and convulsions. It has shown that Gldc deficiency leads to reduced levels of GCS, which correlates with increased ischemic injury in mice [50]. Shmt1 encodes cytoplasmic form of serine hydroxymethyltransferase – enzyme which catalyzes the reversible and THF-dependent conversion of serine to glycine and 5,10-methylene-THF. Mice lacking Shmt1 are viable and fertile, although they exhibit abnormalities in hepatic partitioning of methylenetetrahydrofolate [51]. Gsta4 encodes a glutathione S-transferase, an enzyme that is involved in cellular defense against toxic and carcinogenic compounds. Mice lacking this gene have increased sensitivity to oxidative stress, lower litter size, higher fat content in bones, and greater susceptibility to bacterial infection [52]. Gls2 (GA) is a phosphate-activated glutaminase involved in the glutamine metabolism. The activity of Gls2 is thought to be associated with malignancy and tumor. It has been shown that inhibition of Gls2 expression decreases growth and tumorigenicity of tumor cells [53]. It has been suggested recently that cholesterol plays an essential role in mammalian embryonic development [54]. Developmental disorder might be associated with defects in cholesterol biosynthetic pathways essential for fundamental cellular processes, or with cholesterol transport and uptake impairments cause by defects in apolipoproteins, enzymes, or cell-surface receptors that bind lipoproteins. Several genes in our list of upregulated genes are associated with cholesterol transport or metabolism; Apoc1 and Apoe are also part of the network. Apoc1 is a small circulating lipoprotein associated mostly with HDL-mediated cholesterol transport. Apoc1 deficiency leads to decreased plasma lipid concentration, hepatic lipid accumulation, and increased biliary excretion of cholesterol [55]. Apoe is a ligand for the receptor-mediated catabolism of lipoprotein particles. Knockout of Apoe leads to hypercholesterolemia and, consequently, to an increased susceptibility to the development of atherosclerosis [56]. Lsr (included in the list of 355 upregulated genes) encodes the lipolysis-stimulated lipoprotein receptor – a protein that recognizes apoB/E-containing lipoproteins in the presence of fatty acids. Complete inactivation of Lsr gene is embryonic lethal in mice [57]. The assumption is that Lsr is involved in the clearance of lipoproteins by liver, and is essential for liver and embryonic development.

Akp2 (Alpl, TNAP) encodes a membrane bound enzyme. Akp2 is possibly involved in the process of bone matrix mineralization, by controlling the extracellular levels of PPi concentration required for normal bone mineralization [58]. Mutations in the TNAP gene result in the inborn error of metabolism known as hypophosphatasia [59].

Aquaporin-3 (Aqp3) is a water/glycerol transporting protein. As described in [60], Aqp3-deficient mice were resistant to skin tumors and showed impaired cell proliferation in epidermis, suggesting that Aqp3 might play an important role in skin tumorogenesis. In addition, Aqp3 is expressed in mouse oocytes where it might play an important role in controlling oocyte quality by controlling water permeability [61].

Gpx2 is one of the two isoforms of glutathion peroxidase (GPX), a major enzyme that have a hydrogen peroxide-reducing activity in the epithelium of the gastrointestinal tract. It has been shown mice deficient in Gpx2 and Gpx1 is more sensitive to bacteria-associated intestinal inflammation and cancer [62].

Sorl1 (Lr11) is a member of LDL receptor family involved in the medial-to-intimal migration of vascular smooth muscle cells, the process that is essential to atherosclerotic plaque formation and remodeling of injured arteries [63].

Spint1 (Hai-1) is a Kunitz-type transmembrane serine protease inhibitor that forms inhibitor complexes with several trypsin-like serine proteases and is required for mouse placental development and embryo survival [64]. Disruption of Spint1 causes embryonic lethality around E10.5, growth retardation and placental failure [64], [65].

St14 (Mt-sp1) is a tumor-associated type II transmembrane serine protease highly expressed in epithelial tissues. St14 deletion results in an impaired development of epidermis, hair follicles and immune system [66]. St14-deficient mice die after the birth due to dehydration resulting from the abnormal epidermal barrier function.

Cdh1 encodes a member of a cadherin family of proteins. This calcium dependent cell-cell adhesion glycoprotein is responsible for the cell adhesion of epithelial cells and maintainance of epithelial cell polarity [67]. It has been shown that homozygous mutant embryos fail to compact properly above the morula stage and to form normal blastocysts [67].

Ooep (Floped) and 2410004A20rik (included in the list of 346 upregulated genes) are components of a subcortical maternal complex (SCMC) that is located in subcortex of eggs and is required for the preimplantation mouse development [68]. Although mouse embryo development lacking SCMC is arrested at 2-stage, authors suggest that the defects arise earlier and could result from abnormalities in cytokinesis, cell cycle progression, or mitotic spindle formation.

Genes involved in tight junctions

Tight junction (TJ) is one form of epithelial and endothelial cell-to-cell connection that is required for the normal blastocyst formation and implantation of the embryo [69]. TJs are complexes composed of integral membrane proteins (occludin, claudins, tricellulin, and JAM family members) and peripheral membrane proteins. Integral membrane proteins are represented in our network by claudins (Cldn4, Cldn6 and Cldn7). Claudins play important roles in the architecture and barrier functions of TJs. As described in [69], the inhibition of Cldn4 and Cldn6 affects the normal formation of blastocysts and causes changes in morphology of developing embryos. Tjp2 and Tjp3 are scaffolding proteins that directly link transmembrane TJ proteins to the actin cytoskeleton. It has been shown that the disruption of Tjp2 leads to the death of the embryo due to an inability to complete gastrulation, while Tjp3 knockout does not cause such effect [70].

Other genes in the network

The expression of F-box-containing protein Fbxo15 has been shown to be regulated by Oct4 and Sox2, and has a very similar expression profile to Oct4 [71]. Fbxo15 is possibly involved in an ubiquitin-proteasome pathway to regulate expression levels of proteins required for the pluripotency. However, as has been shown recently [71], a deletion of Fbxo15 does not affect the morphology, proliferation or pluripotency of mouse ES cells.

Dppa3 (Stella) encodes a protein with a SAP-like domain and a splicing factor motif-like structure [72]. It functions as an important maternal factor and is required for the cleavage stages during early mouse embryogenesis; Dppa3 deficient oocytes fail to reach the 8-cell stage or fail to compact [73].

Hmgb2 encodes a member of the non-histone chromosomal high mobility group protein family. It has been reported that high mobility group B (HMGB) chromosomal proteins could be a stimulus for cell differentiation and tumor progression, since HMGB expression correlates with progression of squamous cell carcinoma in skin [74].

Otx2 is a paired-type homeodomain transcription factor, and is involved in the regulation of development of retinal bipolar cells [75]. In addition, Otx2 is required in the neuroectoderm for the development of a forebrain region [76]. Conditional knockout embryos were unable to form a forebrain region correctly [76].

Spint1 (Hai-1) is a Kunitz-type transmembrane serine protease inhibitor that forms inhibitor complexes with several trypsin-like serine proteases and is required for mouse placental development and embryo survival [64]. Disruption of Spint1 causes embryonic lethality around E10.5, growth retardation and placental failure [64], [65].

Genes that are not in the Medusa network but might be important

In addition to the genes described above, there are genes that are included in our list of 355 upregulated genes but are not in the network, as they do not have any high confidence interaction partner defined in String. However some of those genes have been shown to play important roles in self-renewal and pluripotency of mouse ES cells, as well as in mouse embryogenesis.

Phc1 is a member of the Polycomb group of genes, and is a core component of Polycomb repression complex 1 (PRC1). In ES cells the Polycomb complex directly represses genes that have key roles in a variety of developmental processes, including Hox genes [77]. It has been found that genes occupied by PRCs are enriched with trimethylated Lys27 on histone H3, which is known to be a marker of repressive chromatin.

Klf2 and Klf5 belong to the Krüppel-like family of transcription factors. It has been shown that a triple knockdown of Klf2 and Klf5 along with another member of Klf family – Klf4, results in differentiation of mouse ES cells, suggesting an important function of these genes for the maintainance of self-renewal [78]. Recently, Klf5 has been claimed to be essential for normal self-renewal of mouse ES cells and blastocyst development [79]. ES cells lacking Klf5 show increased expression of several differentiation markers and frequent, spontaneous differentiation, while overexpression of Klf5 suppresses the expression of differentiation-related genes and supports LIF-independent self-renewal. The authors suggested that Klf5 contribute to ES cells proliferation by controlling cell-cycle progression and the colony-forming ability.

Mycn (N-myc) is a member of the Myc family of genes, encoding basic helix-loop-helix leucine zipper transcription factors that are potent oncogenes. As reviewed in [80],

Myc family members are implicated to have a function in regulation of cell proliferation, differentiation and apoptosis. The loss of Myc proteins results in inhibited proliferation and cell growth and accelerates differentiation. Deregulated expression of Myc might also induce apoptosis through the induction of DNA damage. It has been shown also that N-myc and c-Myc are interchangeable, and N-myc expression from c-Myc locus rescues embryonic lethality associated with c-Myc deficiency [81].

Rif1 encodes a mouse ortholog of the yeast Rif1 family of telomere-associated proteins. Rif1 has been found to associate with telomeric DNA in mouse ES cells and physically interact with other mouse telomere-associated proteins, suggesting its role in maintenance of telomere length, which needs to be protected from shortening in cells with high proliferation rates [82].

Trim71 (Mlin41) is a mammalian ortholog of *C.elegans* lin-41 heterochronic gene that is known to control the timing of organ formation during development [83]. Based on its homology and expression pattern, the authors suggested that Mlin41 may be involved in regulating specific genes involved in developmental events, including limb formation. It has been shown recently that a disruption of Mlin41 by gene trapping results in neural tube closure defect during development, and embryonic lethality [84].

Tdgf1 (Cr1) is a member of EGF-CFC family of proteins that function as coreceptors for the TGFβ-related proteins, including Nodal. Tdgf1 has been identified to be a target gene in a canonical Wnt/ β-catenin signaling pathway, and its transient activation along with Wnts proteins is necessary for the promotion of self-renewal and pluripotency of ES cells [85].

Functional description of the genes lower expressed in ES and iPS cells compared to fibroblasts in mouse

We analyzed the genes, and hence functions and pathways which are downregulated in ES and iPS cells compared to fibroblasts, which possibly are also genes differently expressed upon differentiation of pluripotent cells at least into some lineages. The network of downregulated genes is shown in supplementary figure S12.

Genes associated with mesenchymal differentiation

During differentiation cells undergo changes in their size, shape, metabolic activity, responsiveness to signals, and chromatin properties. A majority of genes present in our network have functions associated with mesenchymal differentiation which could be explained by the embryologic origin of fibroblasts. They are indeed derived from primitive mesenchyme - a part of embryonic mesoderm capable of developing into connective tissue, bone, cartilage, vascular system (heart and blood vessels), lymphatic tissue, urogenital system (including kidney), and bone marrow. Genes associated with mesenchymal differentiation are described below in more detail.

Col11a1, Col3a1, Col2a1, Col4a1, Col4a2, Col5a1, Col5a2, Col6a1, Col6a2, Col6a3, Col1a1, Col1a2, and Col5a3 belong to the collagen superfamily of proteins. Fibril-

forming collagens (type I, II, III, V and XI) provide structural integrity to all tissues and organs. As reviewed in [86], type I collagen is a major structural component of most connective tissues and is found throughout all the body, except for the cartilage where type II collagen is prevalent. Type III collagen is usually found in the tissues rich with type I collagen, but especially in highly extensible connective tissues, like skin, lung and blood vessels. Minor fibrillar collagens, type V and IX, are associated with tissues rich with collagens I and II respectively. Collagens type IV and VI are non-fibril forming. Type IV collagen is a major component of almost all basement membranes, and defines structural stability and tissue-specific properties [87]. Type VI collagen expression is associated with skeletal muscle development [88].

Lama2 and Lama4 encode laminin - a major non-collagenous component of basement membranes that mediate cell adhesion, growth migration and differentiation. Lama2 knockout mice show muscular dystrophic symptoms, as well as growth retardation [89]. Lama4 deletion results in impaired microvessel growth [90].

Thbs1 and Thbs2 belong to thrombospondin family of proteins. They have been shown to regulate cell-matrix interactions, collagen fibrillogenesis, and angiogenesis [91]. Mice lacking Thbs2 show connective tissue impairment resulted from the disordered collagen fibril formation, and increased vascular density in skin and subcutaneous tissues [92]. Thbs1 null mice have impaired lung homeostasis, associated with multiple-lineage epithelial hyperplasia and the deposition of collagen and elastin [93]. Thbs1 and Thbs2 signaling is performed through their receptor, integrin-associated protein CD47 [94].

Itga11 belongs to the integrins family of multifunctional cell adhesion receptors. Itga11 is a major receptor for fibrillar collagens, and its disruption in mice causes impaired cell migration and collagen reorganization in periodontal ligament fibroblasts, resulting in severely defective incisors and dwarfism with increased mortality [95].

Dcn belongs to the family of small leucine-rich proteoglycans that play roles in generating and maintaining multiple cell-cell and cell-matrix interactions. Dcn has been shown to govern a collagen fibril growth and its disruption leads to abnormal collagen fibril morphology and skin fragility in mice [96].

Fbn1 belongs to fibrillin family of proteins that serve as structural components of microfibrils Fbn1 has been shown to have an essential role in development and maturation of blood vessels [97].

Fbln5 is an extracellular matrix protein that has been shown to be essential for elastic fiber development [98], [99].

Plat encodes plasminogen activator (tPA), one of the major components in the matrix proteolytic network. Plat deficiency in mice results in extensive fibrin deposition that has effects on growth, fertility and survival [100].

Pappa encodes protein from the superfamily of metalloproteinases that cleaves insulin-like growth factor binding proteins (IGFBPs). It has been shown to be an essential growth regulator during mouse embryogenesis and to promote skeletal muscle formation [101], [102].

Postn, periostin, has been shown to function as a molecular switch that can promote the differentiation of mesenchymal cells into a fibroblastic lineage while repressing their transformation into other mesodermal cell lineages [103]. It is involved in the development of noncardiomyocyte lineages of the heart [103], [104].

Lox and Loxl1 encode members of lysyl-oxidase family. These proteins are known to catalyse lysine-derived cross-links in fibrillar collagens and elastin, and are involved in developmental regulation, tumor suppression, and cell motility [105]. Lox has a critical role in cardiovascular and diaphragmatic development in mice [106]. In addition, it has

been shown to regulate TGF-β1 activity in bone and therefore possibly be involved in bone maintenance and development [106]. Loxl1 is required for elastic fiber homeostasis [107]. As has been shown, Loxl1 knockout leads to impairment in connective tissues resulting in urogenital tract disorders in mice [108].

Ltbp1 and Ltbp2 are members of the LTBP/fibrillin family of extracellular proteins. Ltbp1 regulates TGFβ activity and is essential for the heart development in mice [109]. Ltbp2 is involved in the elastic fiber assembly [110].

Mmp2, Mmp3 and Mmp14 belong to the family of matrix metallopeptidases (MMP). MMPs are enzymes involved in extracellular matrix remodeling associated with embryonic development, cancer formation and progression, and other physiological and pathological processes [111]. MMPs can be divided into two subfamilies, membrane (Mmp14) and nonmembrane (Mmp2, Mmp3). An essential role of Mmp2 and Mmp14 in vascular and skeletal muscle development during embryogenesis has been reported [111]. Mmp3 has a role in controlling the synaptic structure at the neuromuscular junctions [112].

Tissue inhibitors of metalloproteinases Timp2 and Timp3 are protein inhibitors of MMPs. The balance between active MMPs and TIMPs is believed to be required to maintain tissue architecture [113]. Timp3 deletion results in a lung dysfunction and a shorter life span of Timp3-null mice [113]. In addition, mice lacking Timp3 develop vessel and retinal abnormalities [114].

Ncam1 encodes multifunctional cell-surface protein associated with epithelial-mesenchymal transition (EMT). Ablation of Ncam1 expression during EMT inhibits focal adhesion assembly, cell spreading and EMT [115].

Cdh2 encodes the adhesion protein N-cadherin. Cdh2-null mice die at E10 with most dramatic cell adhesion defects in heart, showing an essential role of Cdh2 in early heart development [116].

Cav1 is a main structural component of caveolae - invaginations of plasma membrane in cells like fibroblasts and endothelia. Caveolae plays a role in a variety of physiological processes including transport, signaling and tumor suppression. Cav1-deficient mice exhibit impaired function of cardiovascular system and thickening of lung alveolar septa [117]. Ptrf is another component of caveolae that has been shown to be essential for the caveolae formation [118].

Mgp is a mineral-binding extracellular matrix (ECM) protein produced by vascular smooth-muscle cells and chondrocytes. Mgp-deficient mice exhibit inappropriate calcification of various cartilages, and arterial calcification resulting in the blood-vessel rupture and leading to the death within two months after the birth due [119].

Pik3cg belongs to the pi3/pi4-kinase family of proteins that regulate cellular proliferation, apoptosis, cell motility, and adhesion. It has been shown that mice homozygous for null Pik3cg exhibit defective thymocyte development, T cell activation, and neutrophil migration [120].

Cxcl1, Cxcl12 and Pf4 (Cxcl4) are cytokines that belong to the family of chemokines. Cxcl12 is responsible for B-cell lymphopoiesis and bone-marrow myelopoiesis, and mice lacking this gene display late embryonic lethality [121]. Cxcl1 has been described as a potent neutrophil chemoattractant, synthesized in high levels in keratinocytes, monocytes, and macrophages in response to a variety of endogenous stimuli [122]. Pf4 has been shown to have a role in immune resonse and T-cell trafficking [123], as well as thrombosis [124].

Fst, follistatin, is a single chain autocrine glycoprotein that is known as an antagonist of activin and possibly some other members of TGF-β family members. Fst knockout

resulted in multiple defects, including retarded growth, decreased mass of the diaphragm and intercostal muscles, and skeletal abnormalities [125].

Fstl1 is a distantly related homolog of follistatin, mostly expressed in the mesenchymal component of tissues in mice [126]. It has been shown to promote endothelial cell function and stimulate repair of ischemic tissue [127].

Flnc encodes a muscle-specific member of actin binding proteins that is essential for the normal muscular development. Flnc-deficient mice exhibit defects in primary myogenesis and die shortly after birth due to respiratory failure [128].

Acta1 is actin alpha expressed in the skeletal muscle. Acta1 plays an important role in normal muscle formation. It has been shown that mice lacking Acta1 exhibit reduced body size/weight, muscle weakness and die shortly after birth [129].

Acta2 encodes smooth-muscle alpha-actin. This gene is not essential for the formation of cardiovascular system, but plays an important role in regulating vascular contractility and blood pressure homeostasis [130].

Grem2 is a secreted protein, which acts as an antagonist of bone morphogenetic proteins (BMPs). Grem2 acts as a negative regulator of bone formation, and its suppression promotes osteogenesis in vitro [131].

Vcam1 is a cytokine-inducible cell surface protein expressed on vascular endothelium. Mice embryos lacking Vcam1 die during gestation with severe defects in heart or placenta development [132], [133]. It has been shown also that Vcam1-deficient mice develop mild leukocytosis due to the impaired migration of lymphocytes to the bone marrow [134].

Adam12 encodes metalloprotease-disintegrin that has been suggested to be involved in regulating adipogenesis and myogenesis [135].

Crlf1 is a haemopoietin receptor that has a potential role in haemopoiesis, and the recognition or processing of pheromonal signals or for the mechanics of suckling [136].

Csf1 is a secreted cytokine that regulates macrophage differentiation, survival and function [137]. Csf1 has been shown also to play a role in osteoblast-mediated osteoclastogenesis within the bone microenvironment [138].

Cnn1 is a calmodulin and actin binding protein expressed in smooth muscle. It has been shown that mice lacking this gene exhibit increased bone formation and ossification, suggesting that Cnn1 may play a negative role in osteogenesis [139].

Thy1 is a cell-surface signaling molecule that belongs to the immunoglobulin superfamily. Thy1 is involved in multiple signaling cascades to mediate T cell activation, neurite outgrowth, apoptosis, tumor suppression, wound healing, and fibrosis [140].

Lsp1 is an F-actine binding protein expressed in lymphocytes, macrophages and neutrophils. It has been shown that Lsp1 is involved in controlling leukocyte populations in rested and inflamed peritoneum [141].

Growth factors and receptors

Growth factors and their receptors are widely represented in our network. Growth factors play roles in different biological processes, including cellular growth, proliferation and differentiation.

Tgfb3 belongs to the transforming growth factors-beta (TGF-β) family of proteins that have important roles in many developmental processes including regulation of cell proliferation, differentiation, cell adhesion, skeletal development, hematopoiesis,

inflammatory responses, and wound healing [142]. Tgfb3-deficient mice exhibit defective palatogenesis and lung development [143].

Inhba is also a member of TGF-β superfamily. It is mostly produced in the tissues like adrenal gland and gonads, and has been shown to act as an inhibitor of activin and bone morphogenetic protein (BMP) in mouse adrenocortical cell line [144]. In addition, Inhba has a function as endocrine regulator of pituitary follicle-stimulating hormone (FSH) [145].

Bmp1 is another member of TGF-β superfamily. In mouse Bmp1 has procollagen C-proteinase activity, and its deletion results in defective collagen formation, abnormal ventral body wall development, and reduced ossification of the skull [146].

Igf1 is a polypeptide protein hormone that is required for the correct embryonic development in mice, as well as normal growth, glucose metabolism, organ homeostasis, immune and neurologic systems [147]. Insulin-like growth factor 2 (Igf2) has an important role in embryonic growth. It has been demonstrated that Igf2-deficient mice had a significantly reduced body size, but otherwise appeared to be normal and fertile [148]. Igfbp5 is a binding protein known to regulate activity of IGFs. Igfbp5 has a potential role in regulation of craniofacial osteogenesis [149], myogenesis [150], mammary gland morphogenesis [151], and anterior pituitary development [152].

Ptn, a heparin-binding growth-associated molecule (HB-GAM), is a growth factor that has high affinity to heparin. It has been shown to be involved in diverse developmental processes, including formation and plasticity of neuronal connections [153], and osteogenesis [154].

Fgf7 encodes a member of the fibroblast growth factor (FGF) family. Fgf7 is synthesized by cells of mesenchymal origin; it mediates mesenchymal-epithelial interactions [155], and has been shown to be required for the hair development [156].

Dlk1 is a member of epidermal growth factor (EGF)-like gene family. Dlk1 has been shown to be essential for the normal B-cell development and its deficiency results in changes in cell-cell interactions in bone marrow microenvironment [157]. In addition, Dlk1 is important for normal development and homeostasis of adipose tissue [158].

Pdgfra and Pdgfrb encode cell surface tyrosine kinase receptors for members of the platelet-derived growth factor family. PDGF signaling has been shown to regulate proliferation, migration, and differentiation of mesenchymal cell types [159]. Pdgfrb disruption has been shown to result in abnormal kidney development and hematological disorders [160]. Pdgfra null mice show skeletal defects, increased apoptosis, incomplete cephalic closure, impaired myotome and testis formation [161].

Tgfbr2 is a serine/threonin kinase-containing receptor through which TGF-beta signaling is mediated. Tgfbr2 deletion results in embryonic lethality due to an impaired yolk sac hematopoiesis and vasculogenesis [162].

Nrp1 is a multifunctional transmembrane receptor that binds ligands like secreted semaphorins (Sema) and vascular endothelial growth factors (VEGF). The studies have shown that Nrp1 mediates the activity of its ligands during development of the heart, vasculature, and nervous system [71].

Ghr encodes a transmembrane receptor for growth hormone. Ghr is a critical regulator of postnatal growth and metabolism [163]. It has been reported to be essential for the induction and progression of osteogenesis, and regulation of proportion of hematopoietic and mesenchymal cell progenitors in bone marrow [164].

Irs1 encodes a major substrate for insulin receptor and IGF-1 receptor tyrosine kinases. Deficiency in Irs1 results in impaired skeletal growth and resistance to the glucose-lowering effects of insulin [165].

Egfr is a prototype tyrosine kinase receptor activated upon binding of members of epidermal growth factor (EGF) family. It has been shown that Egfr deficiency results in impaired epithelial development in skin, kidney, lung, liver and gastrointestinal tract [166],[167].

Genes associated with the cytoskeleton

Dynamic rearrangements of cytoskeleton promote morphological changes and cellular migration. Recently, it has been shown that cytoskeletal changes also serve as intracellular signal that can control cellular differentiation [168]. Small GTP-binding proteins of Rho family (Rho, Rac, Cdc42) are involved in multiple signal transduction pathways [169] and regulate a variety of cellular functions, including actin-dependent cytoskeleton morphogenesis, microtubular dynamics, and cell motility [170]. Cdc42ep3 belongs to the family of Cdc42 downstream effector proteins that have been shown to induce actin filament assembly leading to cell shape changes in fibroblasts and epithelial cells [171]. Rhoj (TC10L) and Rhoq (TC10A) are isoforms of the protein TC10, belonging to the subfamily of Rho GTP-ases. TC10 is involved in the insulin-stimulated glucose transport in adipocytes [172]. A crucial role of TC10L in early adipocyte differentiation has been suggested recently [173]. Arhgdib is a regulator of activation cycle of Rho proteins in lymphoid organs [174]. Arhgap24 belongs to the RhoGAP family of proteins that act as negative regulators of Rho GTPases [175].

Transcription factors

One expects transcription factors responsible for differentiation to be down-regulated in ES and iPS compared to fibroblasts. Indeed, several such TFs are shown in our network, and even more are included in the list of 462 downregulated genes. Using STRING, we have examined if these TFs are possible interaction partners. The resulting network (STRING confidence 0.15) is shown in Figure S12 (B), and its members are described below in more detail.

Homeodomain-containing genes encode conserved transcription factors that regulate downstream effectors to direct the morphogenetic events leading to the early body development. Hoxb2 is involved in the development of skeleton, nervous system, muscles, and respiratory system. Hoxb2 deficient mice usually die at birth and have a phenotype that is characterized by atrophy and paralysis of the facial muscles of expression, defects in the formation of the sternum and transformations of cervical vertebrae [176]. Hoxa5 plays a role in the establishment of skeleton and specification of axial identity, as well as the respiratory tract development [177]. Hoxc6 has been shown to have a role in the development of mammary glands [178]. Hoxa9 has a specific function in lumbosacral axial skeleton patterning and in limb morphogenesis [179]. Hoxd13 regulates the early formation of limb skeletal elements [180]. Shox2 has been shown to be essential for the palatogenesis in mice [181]. Meox2 is involved in somite morphogenesis and it´s absence results in defective differentiation of limb muscules [182]. Lhx9 is essential for the mouse gonade development and it´s knockout results in a failed proliferation of the somatic cells of the genital ridge [183]. Prrx1 has been shown to regulate epithelial-mesenchymal interactions required for skeletal

organogenesis [184]. Mice lacking Prrx1 exhibit skeletal defects affecting limb, vertebrae or mandible, and die shortly after the birth.

Twist1 and Twist2 are helix-loop-helix transcription factors. Twist1 has a conserved role in mesodermal differentiation, the development of the limb and branchial arches, and neural crest cell migration [185]. Twist1 and Twist2 have been shown to be a part of the regulatory network controlling osteoblast differentiation [186]. Mice lacking Twist1 are characterized by late embryonic lethality [185], while Twist2 deficient mice exhibit postnatal death [187].

Runx1 belongs to the Runt-related TF family of genes, and is essential regulator of hematopoietic system development. Runx1-deficient mice lack hematopoiesis and usually die as an embryo [188]. Runx1t1 and Runx1 create a TF complex which plays an important role in a normal hematopoiesis, and is a frequent target of chromosomal translocations in leukemia [189]. Runx2 also is a memberof the runt-domain gene family and plays an important role in the skeletogenesis. In particular, it is necessary for complete osteoblastic maturation and endochondral bone formation [190]. In addition, it has been shown that Runx2 is expressed in the hematopoietic stem cells compartment, where it modulates Cbfb-smooth muscle myosine heavy chain (SMMHC)-mediated leukemia development [191].

Ets1 and Elk3 are members of Ets transcription factors family. Ets1 is a proto-oncogene that has been shown to have an important role for the survival and activation of T-cells [192]. Elk3 is involved in the vasculogenesis during mouse development. Mice lacking this gene exhibit vascular defect associated with embryonic lymphangiectasis ([193]).

Hmga2 belongs to the HMGA sub-family of high mobility group (HMG) proteins. Hmga2 plays an important role in mammalian growth and development. As has been demonstrated in [194], Hmga2-deficient mice exhibit reduction in growth, dwarfism and infertility. In addition, this gene has been shown to be essential for the normal cardiac development [195].

Sim2 is a member of the basic helix-loop-helix PAS TF gene family. Sim2 knockout in mice results in postnatal lethality due to craniofacial malformations, lung atelectasis and breathing failure [196],[197].

Egr2 is a member of a family of zinc-finger TF. It plays an essential role during hindbrain development in mice. Mice lacking this gene die shortly after birth with the absence of rhombomeres 3 and 5 of the hindbrain affecting axonal migration [198].

Tbx15 belongs to the T-box family of transcriptional regulators. It has been shown recently that Tbx15 has an essential role during mesenchymal stages of skeletal development by maintaining the appropriate rate of cell proliferation [199].

Fosl2 encodes Fos-related protein Fra-2, a TF that has an important role in cartilliage development in mice [200].

Zic1 encodes a zinc-finger TF involved in the cerebellar development [201]. Zic1-deficient mice show cerebellar hypoplasia with a missing lobule of the anterior lobe.

Foxc1 and Foxc2 are two closely-related forkhead/winged helix TFs. They are required for cardiovascular development [202] and have been shown to regulate arterial cell specification and lymphatic sprouting during mouse embryonic development [203]. In addition, Foxc1 is involved in the gonad development, germ cell migration and folliculogenesis [204], and Foxc2 plays a role in skeletogenesis in cells derived from the neural crest and the mesoderm [204,205].

Nr2f2 belongs to the family of nuclear orphan receptors. This gene is involved in mesenchymal-epithelial interactions required for the normal development of vascular system ([206]).

Hic1 encodes a zinc-finger TF which is epigenetically inactivated in cancer. Hic1 functionally cooperates with p53 to suppress cancer in mice, and loss Hic1 function leads to increased tumorigenesis [207].

Evi1 is a zinc-finger-containing TF that plays important role in cell proliferation, vascularization, and development. Evi1 homozygous mutants die at 10.5 days post coitum, and exhibit widespread hypocellularity, hemorrhaging, and impaired development of the heart, somites and cranial ganglia [208].

Maf is a member of basic leucine zipper (bZip) superfamily. It is required for the differentiation of vertebrate lens, and mice lacking Maf exhibit increased embryonic mortality, defective differentiation of lens fiber, and impaired lens development [209]. In addition, Maf has been shown to be necessary for normal differentiation of chondrocytes during endochondral bone development [210].

Zfpm2 (Fog-2) is multi-zinc-finger TF that plays an essential role in cardiac development. Homozygous Zfpm2 mutant embryos die at midgestation with multiple cardiac defects, including absence of coronary vasculature [211],[212].

Nfib, Nfix and Nfia belong to the nuclear factor I (NFI) family of TFs that have a role in regulation of developmental processes. It has been shown that mice lacking Nfib die at birth from respiratory failure due to the severe lung defects, and also exhibit impaired neuronal development [213]. Nfix knockout in mice leads to the postnatal lethality, gastrointestinal defects, hydrocephalus, impaired bone formation, and defects in corpus callosum development [214]. The disruption of Nfia gene results in perinatal lethality due to the developmental defects including hydrocephalus, agenesis of the corpus callosum and hippocampal commissure [215].

Lbh encodes a conserved transcriptional regulatory protein that is expressed during early limb and heart development [216]. Lbh has been suggested to have in important role during normal cardiogenesis since cardiac defects have been observed in mice with deregulated Lbh expression [217].

Ankrd1 (Carp) belongs to the muscle ankyrin repeat protein (MARP) family. MARP family members have been suggested to play in important structural and gene regulatory role in skeletal muscule [218]. Ankrd1 also serves as a genetic marker of cardiac hypertrophy [218].

Snai1 and Snai2 encode zinc-finger proteins that act as transcriptional repressors. Both genes act as mediators of epithelial-mesenchymal transition (EMT) - a conserved developmental mechanism occurring during organogenesis, including mesoderm and neural tube formation [219], [220], [221]. Snai1 knockout leads to the formation of morphologically abnormal mesoderm, and Snai1-deficient embryos show defects in gastrulation [219], [220].

Fhl2 is a member of LIM domain gene family. It has been shown to regulate cell proliferation by activating cyclin D1 expression [222]. Fhl2 is an early marker of cardiogenic cells; however it is not essential for the normal cardiac development in mice [223].

Genes associated with chromatin remodeling

The expression of developmental genes is usually silenced in ES cells and is activated upon differentiation. Changes in chromatin structure are associated with the activation or silencing of genes during development. In our list of 462 downregulated genes we have noticed a gene that functions as an ATP-dependent chromatin remodeler. Chd3 (Mi-2α) is associated with the NuRD co-repressor complex and could act as a transcriptional repressor. However, it has been shown recently that Chd3 acts also as a

transcriptional activator by interacting with early hematopoietic transcription factor c-Myb [224].

Other genes

Ptprz1 belongs to the family of Protein tyrosine phosphatases (PTPs) that are important for the development and functioning of central nervous system [225]. It has been shown recently that Ptprz1 deletion does not interfere normal development of neurons in mice and its loss can be substituted by other PTP proteins [226].

Lrp1 is a receptor protein involved in the endocytosis. It binds various ligands and promotes intracellular signaling that mediates cellular proliferation and migration of different types of cells [227]. It has been shown that Lrp1 disruption in mice results in lethality of embryos around implantation stage [228].

App is an integral membrane protein concentrated mostly in the synapses of neurons, and associated with the pathogenesis of Alzheimer's disease. It has been shown that App-deficient mice exhibit reduced body weight, decreased locomotor activity and forelimb grip strength suggesting impaired neuronal and muscular function [229].

Tnfrsf1a encodes a receptor that mediated the activity of proinflammatory cytokine tumor necrosis factor (TNF) alpha. Tnfrsf1a has an important role in nonspecific immunity, and mice lacking this gene exhibit a high susceptibility to infections [230], [231], [232].

Tnc is an extracellular matrix glycoprotein. This gene has been implicated to play important functional roles in neural development. As has been demonstrated, Tnc deletion did not have a major effect on the central nervous system in mice, but certain forms of synaptic activity were affected [233].

Nbl1 is a secreted bone morphogenic protein (BMP) antagonist belonging to the TGFbeta protein superfamily. Nbl1 has been suggested to play a role as a neuromodulator in inflammatory pain [234].

References

1. Niwa H (2007) How is pluripotency determined and maintained? Development 134: 635-646.

2. Chambers I, Colby D, Robertson M, Nichols J, Lee S, et al. (2003) Functional expression cloning of Nanog, a pluripotency sustaining factor in embryonic stem cells. Cell 113: 643-655.

3. Hay DC, Sutherland L, Clark J, Burdon T (2004) Oct-4 knockdown induces similar patterns of endoderm and trophoblast differentiation markers in human and mouse embryonic stem cells. Stem Cells 22: 225-235.

4. Niwa H, Miyazaki J, Smith AG (2000) Quantitative expression of Oct-3/4 defines differentiation, dedifferentiation or self-renewal of ES cells. Nat Genet 24: 372-376.

5. Masui S, Nakatake Y, Toyooka Y, Shimosato D, Yagi R, et al. (2007) Pluripotency governed by Sox2 via regulation of Oct3/4 expression in mouse embryonic stem cells. Nat Cell Biol 9: 625-635.

6. Lim LS, Loh YH, Zhang W, Li Y, Chen X, et al. (2007) Zic3 is required for maintenance of pluripotency in embryonic stem cells. Mol Biol Cell 18: 1348-1358.

7. Gu P, Goodwin B, Chung AC, Xu X, Wheeler DA, et al. (2005) Orphan nuclear receptor LRH-1 is required to maintain Oct4 expression at the epiblast stage of embryonic development. Mol Cell Biol 25: 3492-3505.

8. Sun C, Nakatake Y, Ura H, Akagi T, Niwa H, et al. (2008) Stem cell-specific expression of Dax1 is conferred by STAT3 and Oct3/4 in embryonic stem cells. Biochem Biophys Res Commun 372: 91-96.

9. Niakan KK, Davis EC, Clipsham RC, Jiang M, Dehart DB, et al. (2006) Novel role for the orphan nuclear receptor Dax1 in embryogenesis, different from steroidogenesis. Mol Genet Metab 88: 261-271.

10. Gu P, LeMenuet D, Chung AC, Mancini M, Wheeler DA, et al. (2005) Orphan nuclear receptor GCNF is required for the repression of pluripotency genes during retinoic acid-induced embryonic stem cell differentiation. Mol Cell Biol 25: 8507-8519.

11. Zhang J, Tam WL, Tong GQ, Wu Q, Chan HY, et al. (2006) Sall4 modulates embryonic stem cell pluripotency and early embryonic development by the transcriptional regulation of Pou5f1. Nat Cell Biol 8: 1114-1123.

12. Yang J, Chai L, Fowles TC, Alipio Z, Xu D, et al. (2008) Genome-wide analysis reveals Sall4 to be a major regulator of pluripotency in murine-embryonic stem cells. Proc Natl Acad Sci U S A 105: 19756-19761.

13. Zhang X, Zhang J, Wang T, Esteban MA, Pei D (2008) Esrrb activates Oct4 transcription and sustains self-renewal and pluripotency in embryonic stem cells. J Biol Chem 283: 35825-35833.

14. Luo J, Sladek R, Bader JA, Matthyssen A, Rossant J, et al. (1997) Placental abnormalities in mouse embryos lacking the orphan nuclear receptor ERR-beta. Nature 388: 778-782.

15. Wang ZX, Teh CH, Kueh JL, Lufkin T, Robson P, et al. (2007) Oct4 and Sox2 directly regulate expression of another pluripotency transcription factor, Zfp206, in embryonic stem cells. J Biol Chem 282: 12822-12830.

16. Zhang W, Walker E, Tamplin OJ, Rossant J, Stanford WL, et al. (2006) Zfp206 regulates ES cell gene expression and differentiation. Nucleic Acids Res 34: 4780-4790.

17. Zhang JZ, Gao W, Yang HB, Zhang B, Zhu ZY, et al. (2006) Screening for genes essential for mouse embryonic stem cell self-renewal using a subtractive RNA interference library. Stem Cells 24: 2661-2668.

18. Masui S, Ohtsuka S, Yagi R, Takahashi K, Ko MS, et al. (2008) Rex1/Zfp42 is dispensable for pluripotency in mouse ES cells. BMC Dev Biol 8: 45.

19. White J, Dalton S (2005) Cell cycle control of embryonic stem cells. Stem Cell Rev 1: 131-138.

20. Burdon T, Smith A, Savatier P (2002) Signalling, cell cycle and pluripotency in embryonic stem cells. Trends Cell Biol 12: 432-438.

21. Tarasov KV, Tarasova YS, Tam WL, Riordon DR, Elliott ST, et al. (2008) B-MYB is essential for normal cell cycle progression and chromosomal stability of embryonic stem cells. PLoS ONE 3: e2478.

22. Xu B, Zhang K, Huang Y (2009) Lin28 modulates cell growth and associates with a subset of cell cycle regulator mRNAs in mouse embryonic stem cells. Rna 15: 357-361.

23. Nishimoto M, Miyagi S, Yamagishi T, Sakaguchi T, Niwa H, et al. (2005) Oct-3/4 maintains the proliferative embryonic stem cell state via specific binding to a variant octamer sequence in the regulatory region of the UTF1 locus. Mol Cell Biol 25: 5084-5094.

24. Ogawa K, Saito A, Matsui H, Suzuki H, Ohtsuka S, et al. (2007) Activin-Nodal signaling is involved in propagation of mouse embryonic stem cells. J Cell Sci 120: 55-65.

25. Levine AJ, Brivanlou AH (2006) GDF3, a BMP inhibitor, regulates cell fate in stem cells and early embryos. Development 133: 209-216.

26. Sharma S, Gurudutta GU, Satija NK, Pati S, Afrin F, et al. (2006) Stem cell c-KIT and HOXB4 genes: critical roles and mechanisms in self-renewal, proliferation, and differentiation. Stem Cells Dev 15: 755-778.

27. Kent D, Copley M, Benz C, Dykstra B, Bowie M, et al. (2008) Regulation of hematopoietic stem cells by the steel factor/KIT signaling pathway. Clin Cancer Res 14: 1926-1930.

28. Bashamboo A, Taylor AH, Samuel K, Panthier JJ, Whetton AD, et al. (2006) The survival of differentiating embryonic stem cells is dependent on the SCF-KIT pathway. J Cell Sci 119: 3039-3046.

29. Kunath T, Saba-El-Leil MK, Almousailleakh M, Wray J, Meloche S, et al. (2007) FGF stimulation of the Erk1/2 signalling cascade triggers transition of pluripotent embryonic stem cells from self-renewal to lineage commitment. Development 134: 2895-2902.

30. Okano M, Bell DW, Haber DA, Li E (1999) DNA methyltransferases Dnmt3a and Dnmt3b are essential for de novo methylation and mammalian development. Cell 99: 247-257.

31. Suetake I, Shinozaki F, Miyagawa J, Takeshima H, Tajima S (2004) DNMT3L stimulates the DNA methylation activity of Dnmt3a and Dnmt3b through a direct interaction. J Biol Chem 279: 27816-27823.

32. Xi S, Zhu H, Xu H, Schmidtmann A, Geiman TM, et al. (2007) Lsh controls Hox gene silencing during development. Proc Natl Acad Sci U S A 104: 14366-14371.

33. Saleque S, Kim J, Rooke HM, Orkin SH (2007) Epigenetic regulation of hematopoietic differentiation by Gfi-1 and Gfi-1b is mediated by the cofactors CoREST and LSD1. Mol Cell 27: 562-572.

34. Ballas N, Grunseich C, Lu DD, Speh JC, Mandel G (2005) REST and its corepressors mediate plasticity of neuronal gene chromatin throughout neurogenesis. Cell 121: 645-657.

35. Hong Y, Cervantes RB, Stambrook PJ (2006) DNA damage response and mutagenesis in mouse embryonic stem cells. Methods Mol Biol 329: 313-326.

36. Snouwaert JN, Gowen LC, Latour AM, Mohn AR, Xiao A, et al. (1999) BRCA1 deficient embryonic stem cells display a decreased homologous recombination frequency and an increased frequency of non-homologous recombination that is corrected by expression of a brca1 transgene. Oncogene 18: 7900-7907.

37. Deng CX, Scott F (2000) Role of the tumor suppressor gene Brca1 in genetic stability and mammary gland tumor formation. Oncogene 19: 1059-1064.

38. Hegan DC, Narayanan L, Jirik FR, Edelmann W, Liskay RM, et al. (2006) Differing patterns of genetic instability in mice deficient in the mismatch repair genes Pms2, Mlh1, Msh2, Msh3 and Msh6. Carcinogenesis 27: 2402-2408.

39. Shen HM, Tanaka A, Bozek G, Nicolae D, Storb U (2006) Somatic hypermutation and class switch recombination in Msh6(-/-)Ung(-/-) double-knockout mice. J Immunol 177: 5386-5392.

40. You Z, Masai H (2008) Cdt1 forms a complex with the minichromosome maintenance protein (MCM) and activates its helicase activity. J Biol Chem 283: 24469-24477.

41. Nishitani H, Lygerou Z (2004) DNA replication licensing. Front Biosci 9: 2115-2132.

42. Kneissl M, Putter V, Szalay AA, Grummt F (2003) Interaction and assembly of murine pre-replicative complex proteins in yeast and mouse cells. J Mol Biol 327: 111-128.

43. Bolcun-Filas E, Speed R, Taggart M, Grey C, de Massy B, et al. (2009) Mutation of the mouse Syce1 gene disrupts synapsis and suggests a link between synaptonemal complex structural components and DNA repair. PLoS Genet 5: e1000393.

44. Bolcun-Filas E, Costa Y, Speed R, Taggart M, Benavente R, et al. (2007) SYCE2 is required for synaptonemal complex assembly, double strand break repair, and homologous recombination. J Cell Biol 176: 741-747.

45. Pan HA, Liao RW, Chung CL, Teng YN, Lin YM, et al. (2008) DAZL protein expression in mouse preimplantation embryo. Fertil Steril 89: 1324-1327.

46. Jiao X, Trifillis P, Kiledjian M (2002) Identification of target messenger RNA substrates for the murine deleted in azoospermia-like RNA-binding protein. Biol Reprod 66: 475-485.

47. Cauffman G, Van de Velde H, Liebaers I, Van Steirteghem A (2005) DAZL expression in human oocytes, preimplantation embryos and embryonic stem cells. Mol Hum Reprod 11: 405-411.

48. Reynolds N, Collier B, Maratou K, Bingham V, Speed RM, et al. (2005) Dazl binds in vivo to specific transcripts and can regulate the pre-meiotic translation of Mvh in germ cells. Hum Mol Genet 14: 3899-3909.

49. Morrish F, Neretti N, Sedivy JM, Hockenbery DM (2008) The oncogene c-Myc coordinates regulation of metabolic networks to enable rapid cell cycle entry. Cell Cycle 7: 1054-1066.

50. Oda M, Kure S, Sugawara T, Yamaguchi S, Kojima K, et al. (2007) Direct correlation between ischemic injury and extracellular glycine concentration in mice with genetically altered activities of the glycine cleavage multienzyme system. Stroke 38: 2157-2164.

51. MacFarlane AJ, Liu X, Perry CA, Flodby P, Allen RH, et al. (2008) Cytoplasmic serine hydroxymethyltransferase regulates the metabolic partitioning of methylenetetrahydrofolate but is not essential in mice. J Biol Chem 283: 25846-25853.

52. Engle MR, Singh SP, Czernik PJ, Gaddy D, Montague DC, et al. (2004) Physiological role of mGSTA4-4, a glutathione S-transferase metabolizing 4-hydroxynonenal: generation and analysis of mGsta4 null mouse. Toxicol Appl Pharmacol 194: 296-308.

53. Lobo C, Ruiz-Bellido MA, Aledo JC, Marquez J, Nunez De Castro I, et al. (2000) Inhibition of glutaminase expression by antisense mRNA decreases growth and tumourigenicity of tumour cells. Biochem J 348 Pt 2: 257-261.

54. Farese RV, Jr., Herz J (1998) Cholesterol metabolism and embryogenesis. Trends Genet 14: 115-120.

55. Gautier T, Tietge UJ, Boverhof R, Perton FG, Le Guern N, et al. (2007) Hepatic lipid accumulation in apolipoprotein C-I-deficient mice is potentiated by cholesteryl ester transfer protein. J Lipid Res 48: 30-40.

56. van Ree JH, van den Broek WJ, Dahlmans VE, Groot PH, Vidgeon-Hart M, et al. (1994) Diet-induced hypercholesterolemia and atherosclerosis in heterozygous apolipoprotein E-deficient mice. Atherosclerosis 111: 25-37.

57. Mesli S, Javorschi S, Berard AM, Landry M, Priddle H, et al. (2004) Distribution of the lipolysis stimulated receptor in adult and embryonic murine tissues and lethality of LSR-/- embryos at 12.5 to 14.5 days of gestation. Eur J Biochem 271: 3103-3114.

58. Hessle L, Johnson KA, Anderson HC, Narisawa S, Sali A, et al. (2002) Tissue-nonspecific alkaline phosphatase and plasma cell membrane glycoprotein-1 are central antagonistic regulators of bone mineralization. Proc Natl Acad Sci U S A 99: 9445-9449.

59. Henthorn PS, Whyte MP (1992) Missense mutations of the tissue-nonspecific alkaline phosphatase gene in hypophosphatasia. Clin Chem 38: 2501-2505.

60. Hara-Chikuma M, Verkman AS (2008) Prevention of skin tumorigenesis and impairment of epidermal cell proliferation by targeted aquaporin-3 gene disruption. Mol Cell Biol 28: 326-332.

61. Meng QX, Gao HJ, Xu CM, Dong MY, Sheng X, et al. (2008) Reduced expression and function of aquaporin-3 in mouse metaphase-II oocytes induced by controlled ovarian hyperstimulation were associated with subsequent low fertilization rate. Cell Physiol Biochem 21: 123-128.

62. Chu FF, Esworthy RS, Chu PG, Longmate JA, Huycke MM, et al. (2004) Bacteria-induced intestinal cancer in mice with disrupted Gpx1 and Gpx2 genes. Cancer Res 64: 962-968.

63. Jiang M, Bujo H, Ohwaki K, Unoki H, Yamazaki H, et al. (2008) Ang II-stimulated migration of vascular smooth muscle cells is dependent on LR11 in mice. J Clin Invest 118: 2733-2746.

64. Szabo R, Molinolo A, List K, Bugge TH (2007) Matriptase inhibition by hepatocyte growth factor activator inhibitor-1 is essential for placental development. Oncogene 26: 1546-1556.

65. Tanaka H, Nagaike K, Takeda N, Itoh H, Kohama K, et al. (2005) Hepatocyte growth factor activator inhibitor type 1 (HAI-1) is required for branching morphogenesis in the chorioallantoic placenta. Mol Cell Biol 25: 5687-5698.

66. List K, Haudenschild CC, Szabo R, Chen W, Wahl SM, et al. (2002) Matriptase/MT-SP1 is required for postnatal survival, epidermal barrier function, hair follicle development, and thymic homeostasis. Oncogene 21: 3765-3779.

67. Riethmacher D, Brinkmann V, Birchmeier C (1995) A targeted mutation in the mouse E-cadherin gene results in defective preimplantation development. Proc Natl Acad Sci U S A 92: 855-859.

68. Li L, Baibakov B, Dean J (2008) A subcortical maternal complex essential for preimplantation mouse embryogenesis. Dev Cell 15: 416-425.

69. Moriwaki K, Tsukita S, Furuse M (2007) Tight junctions containing claudin 4 and 6 are essential for blastocyst formation in preimplantation mouse embryos. Dev Biol 312: 509-522.

70. Xu J, Kausalya PJ, Phua DC, Ali SM, Hossain Z, et al. (2008) Early embryonic lethality of mice lacking ZO-2, but Not ZO-3, reveals critical and nonredundant roles for individual zonula occludens proteins in mammalian development. Mol Cell Biol 28: 1669-1678.

71. Tokuzawa Y, Kaiho E, Maruyama M, Takahashi K, Mitsui K, et al. (2003) Fbx15 is a novel target of Oct3/4 but is dispensable for embryonic stem cell self-renewal and mouse development. Mol Cell Biol 23: 2699-2708.

72. Payer B, Saitou M, Barton SC, Thresher R, Dixon JP, et al. (2003) Stella is a maternal effect gene required for normal early development in mice. Curr Biol 13: 2110-2117.

73. Bortvin A, Goodheart M, Liao M, Page DC (2004) Dppa3 / Pgc7 / stella is a maternal factor and is not required for germ cell specification in mice. BMC Dev Biol 4: 2.

74. Sharma A, Ray R, Rajeswari MR (2008) Overexpression of high mobility group (HMG) B1 and B2 proteins directly correlates with the progression of squamous cell carcinoma in skin. Cancer Invest 26: 843-851.

75. Kim DS, Matsuda T, Cepko CL (2008) A core paired-type and POU homeodomain-containing transcription factor program drives retinal bipolar cell gene expression. J Neurosci 28: 7748-7764.

76. Tian E, Kimura C, Takeda N, Aizawa S, Matsuo I (2002) Otx2 is required to respond to signals from anterior neural ridge for forebrain specification. Dev Biol 242: 204-223.

77. Boyer LA, Plath K, Zeitlinger J, Brambrink T, Medeiros LA, et al. (2006) Polycomb complexes repress developmental regulators in murine embryonic stem cells. Nature 441: 349-353.

78. Jiang J, Chan YS, Loh YH, Cai J, Tong GQ, et al. (2008) A core Klf circuitry regulates self-renewal of embryonic stem cells. Nat Cell Biol 10: 353-360.

79. Ema M, Mori D, Niwa H, Hasegawa Y, Yamanaka Y, et al. (2008) Kruppel-like factor 5 is essential for blastocyst development and the normal self-renewal of mouse ESCs. Cell Stem Cell 3: 555-567.

80. Adhikary S, Eilers M (2005) Transcriptional regulation and transformation by Myc proteins. Nat Rev Mol Cell Biol 6: 635-645.

81. Malynn BA, de Alboran IM, O'Hagan RC, Bronson R, Davidson L, et al. (2000) N-myc can functionally replace c-myc in murine development, cellular growth, and differentiation. Genes Dev 14: 1390-1399.

82. Adams IR, McLaren A (2004) Identification and characterisation of mRif1: a mouse telomere-associated protein highly expressed in germ cells and embryo-derived pluripotent stem cells. Dev Dyn 229: 733-744.

83. Schulman BR, Esquela-Kerscher A, Slack FJ (2005) Reciprocal expression of lin-41 and the microRNAs let-7 and mir-125 during mouse embryogenesis. Dev Dyn 234: 1046-1054.

84. Maller Schulman BR, Liang X, Stahlhut C, DelConte C, Stefani G, et al. (2008) The let-7 microRNA target gene, Mlin41/Trim71 is required for mouse embryonic survival and neural tube closure. Cell Cycle 7: 3935-3942.

85. Strizzi L, Bianco C, Normanno N, Salomon D (2005) Cripto-1: a multifunctional modulator during embryogenesis and oncogenesis. Oncogene 24: 5731-5741.

86. Kadler KE, Holmes DF, Trotter JA, Chapman JA (1996) Collagen fibril formation. Biochem J 316 (Pt 1): 1-11.

87. Van Agtmael T, Schlotzer-Schrehardt U, McKie L, Brownstein DG, Lee AW, et al. (2005) Dominant mutations of Col4a1 result in basement membrane defects which lead to anterior segment dysgenesis and glomerulopathy. Hum Mol Genet 14: 3161-3168.

88. Braghetta P, Ferrari A, Fabbro C, Bizzotto D, Volpin D, et al. (2008) An enhancer required for transcription of the Col6a1 gene in muscle connective tissue is induced by signals released from muscle cells. Exp Cell Res 314: 3508-3518.

89. Miyagoe Y, Hanaoka K, Nonaka I, Hayasaka M, Nabeshima Y, et al. (1997) Laminin alpha2 chain-null mutant mice by targeted disruption of the Lama2 gene: a new model of merosin (laminin 2)-deficient congenital muscular dystrophy. FEBS Lett 415: 33-39.

90. Thyboll J, Kortesmaa J, Cao R, Soininen R, Wang L, et al. (2002) Deletion of the laminin alpha4 chain leads to impaired microvessel maturation. Mol Cell Biol 22: 1194-1202.

91. Bornstein P, Agah A, Kyriakides TR (2004) The role of thrombospondins 1 and 2 in the regulation of cell-matrix interactions, collagen fibril formation, and the response to injury. Int J Biochem Cell Biol 36: 1115-1125.

92. Kyriakides TR, Zhu YH, Smith LT, Bain SD, Yang Z, et al. (1998) Mice that lack thrombospondin 2 display connective tissue abnormalities that are associated with disordered collagen fibrillogenesis, an increased vascular density, and a bleeding diathesis. J Cell Biol 140: 419-430.

93. Lawler J, Sunday M, Thibert V, Duquette M, George EL, et al. (1998) Thrombospondin-1 is required for normal murine pulmonary homeostasis and its absence causes pneumonia. J Clin Invest 101: 982-992.

94. Brown EJ, Frazier WA (2001) Integrin-associated protein (CD47) and its ligands. Trends Cell Biol 11: 130-135.

95. Popova SN, Barczyk M, Tiger CF, Beertsen W, Zigrino P, et al. (2007) Alpha11 beta1 integrin-dependent regulation of periodontal ligament function in the erupting mouse incisor. Mol Cell Biol 27: 4306-4316.

96. Danielson KG, Baribault H, Holmes DF, Graham H, Kadler KE, et al. (1997) Targeted disruption of decorin leads to abnormal collagen fibril morphology and skin fragility. J Cell Biol 136: 729-743.

97. Carta L, Pereira L, Arteaga-Solis E, Lee-Arteaga SY, Lenart B, et al. (2006) Fibrillins 1 and 2 perform partially overlapping functions during aortic development. J Biol Chem 281: 8016-8023.

98. Nakamura T, Lozano PR, Ikeda Y, Iwanaga Y, Hinek A, et al. (2002) Fibulin-5/DANCE is essential for elastogenesis in vivo. Nature 415: 171-175.

99. Yanagisawa H, Davis EC, Starcher BC, Ouchi T, Yanagisawa M, et al. (2002) Fibulin-5 is an elastin-binding protein essential for elastic fibre development in vivo. Nature 415: 168-171.

100. Carmeliet P, Schoonjans L, Kieckens L, Ream B, Degen J, et al. (1994) Physiological consequences of loss of plasminogen activator gene function in mice. Nature 368: 419-424.

101. Conover CA, Bale LK, Overgaard MT, Johnstone EW, Laursen UH, et al. (2004) Metalloproteinase pregnancy-associated plasma protein A is a critical growth regulatory factor during fetal development. Development 131: 1187-1194.

102. Rehage M, Mohan S, Wergedal JE, Bonafede B, Tran K, et al. (2007) Transgenic overexpression of pregnancy-associated plasma protein-A increases the somatic growth and skeletal muscle mass in mice. Endocrinology 148: 6176-6185.

103. Norris RA, Moreno-Rodriguez RA, Sugi Y, Hoffman S, Amos J, et al. (2008) Periostin regulates atrioventricular valve maturation. Dev Biol 316: 200-213.

104. Snider P, Hinton RB, Moreno-Rodriguez RA, Wang J, Rogers R, et al. (2008) Periostin is required for maturation and extracellular matrix stabilization of noncardiomyocyte lineages of the heart. Circ Res 102: 752-760.

105. Csiszar K (2001) Lysyl oxidases: a novel multifunctional amine oxidase family. Prog Nucleic Acid Res Mol Biol 70: 1-32.

106. Hornstra IK, Birge S, Starcher B, Bailey AJ, Mecham RP, et al. (2003) Lysyl oxidase is required for vascular and diaphragmatic development in mice. J Biol Chem 278: 14387-14393.

107. Liu X, Zhao Y, Gao J, Pawlyk B, Starcher B, et al. (2004) Elastic fiber homeostasis requires lysyl oxidase-like 1 protein. Nat Genet 36: 178-182.

108. Lee UJ, Gustilo-Ashby AM, Daneshgari F, Kuang M, Vurbic D, et al. (2008) Lower urogenital tract anatomical and functional phenotype in lysyl oxidase like-1 knockout mice resembles female pelvic floor dysfunction in humans. Am J Physiol Renal Physiol 295: F545-555.

109. Todorovic V, Frendewey D, Gutstein DE, Chen Y, Freyer L, et al. (2007) Long form of latent TGF-beta binding protein 1 (Ltbp1L) is essential for cardiac outflow tract septation and remodeling. Development 134: 3723-3732.

110. Hirai M, Horiguchi M, Ohbayashi T, Kita T, Chien KR, et al. (2007) Latent TGF-beta-binding protein 2 binds to DANCE/fibulin-5 and regulates elastic fiber assembly. Embo J 26: 3283-3295.

111. Oh J, Takahashi R, Adachi E, Kondo S, Kuratomi S, et al. (2004) Mutations in two matrix metalloproteinase genes, MMP-2 and MT1-MMP, are synthetic lethal in mice. Oncogene 23: 5041-5048.

112. VanSaun M, Herrera AA, Werle MJ (2003) Structural alterations at the neuromuscular junctions of matrix metalloproteinase 3 null mutant mice. J Neurocytol 32: 1129-1142.

113. Leco KJ, Waterhouse P, Sanchez OH, Gowing KL, Poole AR, et al. (2001) Spontaneous air space enlargement in the lungs of mice lacking tissue inhibitor of metalloproteinases-3 (TIMP-3). J Clin Invest 108: 817-829.

114. Janssen A, Hoellenriegel J, Fogarasi M, Schrewe H, Seeliger M, et al. (2008) Abnormal vessel formation in the choroid of mice lacking tissue inhibitor of metalloprotease-3. Invest Ophthalmol Vis Sci 49: 2812-2822.

115. Lehembre F, Yilmaz M, Wicki A, Schomber T, Strittmatter K, et al. (2008) NCAM-induced focal adhesion assembly: a functional switch upon loss of E-cadherin. Embo J 27: 2603-2615.

116. Radice GL, Rayburn H, Matsunami H, Knudsen KA, Takeichi M, et al. (1997) Developmental defects in mouse embryos lacking N-cadherin. Dev Biol 181: 64-78.

117. Drab M, Verkade P, Elger M, Kasper M, Lohn M, et al. (2001) Loss of caveolae, vascular dysfunction, and pulmonary defects in caveolin-1 gene-disrupted mice. Science 293: 2449-2452.

118. Liu L, Brown D, McKee M, Lebrasseur NK, Yang D, et al. (2008) Deletion of Cavin/PTRF causes global loss of caveolae, dyslipidemia, and glucose intolerance. Cell Metab 8: 310-317.

119. Luo G, Ducy P, McKee MD, Pinero GJ, Loyer E, et al. (1997) Spontaneous calcification of arteries and cartilage in mice lacking matrix GLA protein. Nature 386: 78-81.

120. Sasaki T, Irie-Sasaki J, Jones RG, Oliveira-dos-Santos AJ, Stanford WL, et al. (2000) Function of PI3Kgamma in thymocyte development, T cell activation, and neutrophil migration. Science 287: 1040-1046.

121. Nagasawa T, Hirota S, Tachibana K, Takakura N, Nishikawa S, et al. (1996) Defects of B-cell lymphopoiesis and bone-marrow myelopoiesis in mice lacking the CXC chemokine PBSF/SDF-1. Nature 382: 635-638.

122. Shea-Donohue T, Thomas K, Cody MJ, Aiping Z, Detolla LJ, et al. (2008) Mice deficient in the CXCR2 ligand, CXCL1 (KC/GRO-alpha), exhibit increased susceptibility to dextran sodium sulfate (DSS)-induced colitis. Innate Immun 14: 117-124.

123. Srivastava K, Cockburn IA, Swaim A, Thompson LE, Tripathi A, et al. (2008) Platelet factor 4 mediates inflammation in experimental cerebral malaria. Cell Host Microbe 4: 179-187.

124. Eslin DE, Zhang C, Samuels KJ, Rauova L, Zhai L, et al. (2004) Transgenic mice studies demonstrate a role for platelet factor 4 in thrombosis: dissociation between anticoagulant and antithrombotic effect of heparin. Blood 104: 3173-3180.

125. Matzuk MM, Lu N, Vogel H, Sellheyer K, Roop DR, et al. (1995) Multiple defects and perinatal death in mice deficient in follistatin. Nature 374: 360-363.

126. Adams D, Larman B, Oxburgh L (2007) Developmental expression of mouse Follistatin-like 1 (Fstl1): Dynamic regulation during organogenesis of the kidney and lung. Gene Expr Patterns 7: 491-500.

127. Ouchi N, Oshima Y, Ohashi K, Higuchi A, Ikegami C, et al. (2008) Follistatin-like 1, a secreted muscle protein, promotes endothelial cell function and revascularization in ischemic tissue through a nitric-oxide synthase-dependent mechanism. J Biol Chem 283: 32802-32811.

128. Dalkilic I, Schienda J, Thompson TG, Kunkel LM (2006) Loss of FilaminC (FLNc) results in severe defects in myogenesis and myotube structure. Mol Cell Biol 26: 6522-6534.

129. Crawford K, Flick R, Close L, Shelly D, Paul R, et al. (2002) Mice lacking skeletal muscle actin show reduced muscle strength and growth deficits and die during the neonatal period. Mol Cell Biol 22: 5887-5896.

130. Schildmeyer LA, Braun R, Taffet G, Debiasi M, Burns AE, et al. (2000) Impaired vascular contractility and blood pressure homeostasis in the smooth muscle alpha-actin null mouse. Faseb J 14: 2213-2220.

131. Ideno H, Takanabe R, Shimada A, Imaizumi K, Araki R, et al. (2009) Protein related to DAN and cerberus (PRDC) inhibits osteoblastic differentiation and its suppression promotes osteogenesis in vitro. Exp Cell Res 315: 474-484.

132. Kwee L, Baldwin HS, Shen HM, Stewart CL, Buck C, et al. (1995) Defective development of the embryonic and extraembryonic circulatory systems in vascular cell adhesion molecule (VCAM-1) deficient mice. Development 121: 489-503.

133. Gurtner GC, Davis V, Li H, McCoy MJ, Sharpe A, et al. (1995) Targeted disruption of the murine VCAM1 gene: essential role of VCAM-1 in chorioallantoic fusion and placentation. Genes Dev 9: 1-14.

134. Koni PA, Joshi SK, Temann UA, Olson D, Burkly L, et al. (2001) Conditional vascular cell adhesion molecule 1 deletion in mice: impaired lymphocyte migration to bone marrow. J Exp Med 193: 741-754.

135. Kurisaki T, Masuda A, Sudo K, Sakagami J, Higashiyama S, et al. (2003) Phenotypic analysis of Meltrin alpha (ADAM12)-deficient mice: involvement of Meltrin alpha in adipogenesis and myogenesis. Mol Cell Biol 23: 55-61.

136. Alexander WS, Rakar S, Robb L, Farley A, Willson TA, et al. (1999) Suckling defect in mice lacking the soluble haemopoietin receptor NR6. Curr Biol 9: 605-608.

137. Irvine KM, Burns CJ, Wilks AF, Su S, Hume DA, et al. (2006) A CSF-1 receptor kinase inhibitor targets effector functions and inhibits pro-inflammatory cytokine production from murine macrophage populations. Faseb J 20: 1921-1923.

138. Yao GQ, Sun BH, Weir EC, Insogna KL (2002) A role for cell-surface CSF-1 in osteoblast-mediated osteoclastogenesis. Calcif Tissue Int 70: 339-346.

139. Yoshikawa H, Taniguchi SI, Yamamura H, Mori S, Sugimoto M, et al. (1998) Mice lacking smooth muscle calponin display increased bone formation that is associated with enhancement of bone morphogenetic protein responses. Genes Cells 3: 685-695.

140. Rege TA, Hagood JS (2006) Thy-1, a versatile modulator of signaling affecting cellular adhesion, proliferation, survival, and cytokine/growth factor responses. Biochim Biophys Acta 1763: 991-999.

141. Jongstra-Bilen J, Misener VL, Wang C, Ginzberg H, Auerbach A, et al. (2000) LSP1 modulates leukocyte populations in resting and inflamed peritoneum. Blood 96: 1827-1835.

142. Dunker N, Krieglstein K (2000) Targeted mutations of transforming growth factor-beta genes reveal important roles in mouse development and adult homeostasis. Eur J Biochem 267: 6982-6988.

143. Kaartinen V, Voncken JW, Shuler C, Warburton D, Bu D, et al. (1995) Abnormal lung development and cleft palate in mice lacking TGF-beta 3 indicates defects of epithelial-mesenchymal interaction. Nat Genet 11: 415-421.

144. Farnworth PG, Stanton PG, Wang Y, Escalona R, Findlay JK, et al. (2006) Inhibins differentially antagonize activin and bone morphogenetic protein action in a mouse adrenocortical cell line. Endocrinology 147: 3462-3471.

145. Bernard DJ, Chapman SC, Woodruff TK (2001) Mechanisms of inhibin signal transduction. Recent Prog Horm Res 56: 417-450.

146. Suzuki N, Labosky PA, Furuta Y, Hargett L, Dunn R, et al. (1996) Failure of ventral body wall closure in mouse embryos lacking a procollagen C-proteinase encoded by Bmp1, a mammalian gene related to Drosophila tolloid. Development 122: 3587-3595.

147. Powell-Braxton L, Hollingshead P, Warburton C, Dowd M, Pitts-Meek S, et al. (1993) IGF-I is required for normal embryonic growth in mice. Genes Dev 7: 2609-2617.

148. DeChiara TM, Efstratiadis A, Robertson EJ (1990) A growth-deficiency phenotype in heterozygous mice carrying an insulin-like growth factor II gene disrupted by targeting. Nature 345: 78-80.

149. Bobola N, Engist B (2008) IGFBP5 is a potential regulator of craniofacial skeletogenesis. Genesis 46: 52-59.

150. Cobb LJ, Salih DA, Gonzalez I, Tripathi G, Carter EJ, et al. (2004) Partitioning of IGFBP-5 actions in myogenesis: IGF-independent anti-apoptotic function. J Cell Sci 117: 1737-1746.

151. Ning Y, Hoang B, Schuller AG, Cominski TP, Hsu MS, et al. (2007) Delayed mammary gland involution in mice with mutation of the insulin-like growth factor binding protein 5 gene. Endocrinology 148: 2138-2147.

152. Holley SJ, Hall SB, Mellon PL (2002) Complementary expression of IGF-II and IGFBP-5 during anterior pituitary development. Dev Biol 244: 319-328.

153. Amet LE, Lauri SE, Hienola A, Croll SD, Lu Y, et al. (2001) Enhanced hippocampal long-term potentiation in mice lacking heparin-binding growth-associated molecule. Mol Cell Neurosci 17: 1014-1024.

154. Tare RS, Oreffo RO, Clarke NM, Roach HI (2002) Pleiotrophin/Osteoblast-stimulating factor 1: dissecting its diverse functions in bone formation. J Bone Miner Res 17: 2009-2020.

155. Rubin JS, Bottaro DP, Chedid M, Miki T, Ron D, et al. (1995) Keratinocyte growth factor as a cytokine that mediates mesenchymal-epithelial interaction. Exs 74: 191-214.

156. Guo L, Degenstein L, Fuchs E (1996) Keratinocyte growth factor is required for hair development but not for wound healing. Genes Dev 10: 165-175.

157. Raghunandan R, Ruiz-Hidalgo M, Jia Y, Ettinger R, Rudikoff E, et al. (2008) Dlk1 influences differentiation and function of B lymphocytes. Stem Cells Dev 17: 495-507.

158. Moon YS, Smas CM, Lee K, Villena JA, Kim KH, et al. (2002) Mice lacking paternally expressed Pref-1/Dlk1 display growth retardation and accelerated adiposity. Mol Cell Biol 22: 5585-5592.

159. Hoch RV, Soriano P (2003) Roles of PDGF in animal development. Development 130: 4769-4784.

160. Soriano P (1994) Abnormal kidney development and hematological disorders in PDGF beta-receptor mutant mice. Genes Dev 8: 1888-1896.

161. Soriano P (1997) The PDGF alpha receptor is required for neural crest cell development and for normal patterning of the somites. Development 124: 2691-2700.

162. Oshima M, Oshima H, Taketo MM (1996) TGF-beta receptor type II deficiency results in defects of yolk sac hematopoiesis and vasculogenesis. Dev Biol 179: 297-302.

163. Rowland JE, Lichanska AM, Kerr LM, White M, d'Aniello EM, et al. (2005) In vivo analysis of growth hormone receptor signaling domains and their associated transcripts. Mol Cell Biol 25: 66-77.

164. Cool SM, Grunert M, Jackson R, Li H, Nurcombe V, et al. (2005) Role of growth hormone receptor signaling in osteogenesis from murine bone marrow progenitor cells. Biochem Biophys Res Commun 338: 1048-1058.

165. Tamemoto H, Kadowaki T, Tobe K, Yagi T, Sakura H, et al. (1994) Insulin resistance and growth retardation in mice lacking insulin receptor substrate-1. Nature 372: 182-186.

166. Threadgill DW, Dlugosz AA, Hansen LA, Tennenbaum T, Lichti U, et al. (1995) Targeted disruption of mouse EGF receptor: effect of genetic background on mutant phenotype. Science 269: 230-234.

167. Miettinen PJ, Berger JE, Meneses J, Phung Y, Pedersen RA, et al. (1995) Epithelial immaturity and multiorgan failure in mice lacking epidermal growth factor receptor. Nature 376: 337-341.

168. Higuchi C, Nakamura N, Yoshikawa H, Itoh K (2009) Transient dynamic actin cytoskeletal change stimulates the osteoblastic differentiation. J Bone Miner Metab 27: 158-167.

169. Etienne-Manneville S, Hall A (2002) Rho GTPases in cell biology. Nature 420: 629-635.

170. Jaffe AB, Hall A (2005) Rho GTPases: biochemistry and biology. Annu Rev Cell Dev Biol 21: 247-269.

171. Hirsch DS, Pirone DM, Burbelo PD (2001) A new family of Cdc42 effector proteins, CEPs, function in fibroblast and epithelial cell shape changes. J Biol Chem 276: 875-883.

172. Chiang SH, Hou JC, Hwang J, Pessin JE, Saltiel AR (2002) Cloning and functional characterization of related TC10 isoforms, a subfamily of Rho proteins involved in insulin-stimulated glucose transport. J Biol Chem 277: 13067-13073.

173. Nishizuka M, Arimoto E, Tsuchiya T, Nishihara T, Imagawa M (2003) Crucial role of TCL/TC10beta L, a subfamily of Rho GTPase, in adipocyte differentiation. J Biol Chem 278: 15279-15284.

174. Ishizaki H, Togawa A, Tanaka-Okamoto M, Hori K, Nishimura M, et al. (2006) Defective chemokine-directed lymphocyte migration and development in the absence of Rho guanosine diphosphate-dissociation inhibitors alpha and beta. J Immunol 177: 8512-8521.

175. Katoh M, Katoh M (2004) Identification and characterization of ARHGAP24 and ARHGAP25 genes in silico. Int J Mol Med 14: 333-338.

176. Barrow JR, Capecchi MR (1996) Targeted disruption of the Hoxb-2 locus in mice interferes with expression of Hoxb-1 and Hoxb-4. Development 122: 3817-3828.

177. Jeannotte L, Lemieux M, Charron J, Poirier F, Robertson EJ (1993) Specification of axial identity in the mouse: role of the Hoxa-5 (Hox1.3) gene. Genes Dev 7: 2085-2096.

178. Garcia-Gasca A, Spyropoulos DD (2000) Differential mammary morphogenesis along the anteroposterior axis in Hoxc6 gene targeted mice. Dev Dyn 219: 261-276.

179. Fromental-Ramain C, Warot X, Lakkaraju S, Favier B, Haack H, et al. (1996) Specific and redundant functions of the paralogous Hoxa-9 and Hoxd-9 genes in forelimb and axial skeleton patterning. Development 122: 461-472.

180. Salsi V, Vigano MA, Cocchiarella F, Mantovani R, Zappavigna V (2008) Hoxd13 binds in vivo and regulates the expression of genes acting in key pathways for early limb and skeletal patterning. Dev Biol 317: 497-507.

181. Yu L, Gu S, Alappat S, Song Y, Yan M, et al. (2005) Shox2-deficient mice exhibit a rare type of incomplete clefting of the secondary palate. Development 132: 4397-4406.

182. Mankoo BS, Skuntz S, Harrigan I, Grigorieva E, Candia A, et al. (2003) The concerted action of Meox homeobox genes is required upstream of genetic pathways essential for the formation, patterning and differentiation of somites. Development 130: 4655-4664.

183. Birk OS, Casiano DE, Wassif CA, Cogliati T, Zhao L, et al. (2000) The LIM homeobox gene Lhx9 is essential for mouse gonad formation. Nature 403: 909-913.

184. Martin JF, Bradley A, Olson EN (1995) The paired-like homeo box gene MHox is required for early events of skeletogenesis in multiple lineages. Genes Dev 9: 1237-1249.

185. O'Rourke MP, Tam PP (2002) Twist functions in mouse development. Int J Dev Biol 46: 401-413.

186. Zhang Y, Hassan MQ, Li ZY, Stein JL, Lian JB, et al. (2008) Intricate gene regulatory networks of helix-loop-helix (HLH) proteins support regulation of bone-tissue related genes during osteoblast differentiation. J Cell Biochem 105: 487-496.

187. Sosic D, Richardson JA, Yu K, Ornitz DM, Olson EN (2003) Twist regulates cytokine gene expression through a negative feedback loop that represses NF-kappaB activity. Cell 112: 169-180.

188. Samokhvalov IM, Thomson AM, Lalancette C, Liakhovitskaia A, Ure J, et al. (2006) Multifunctional reversible knockout/reporter system enabling fully functional reconstitution of the AML1/Runx1 locus and rescue of hematopoiesis. Genesis 44: 115-121.

189. Higuchi M, O'Brien D, Kumaravelu P, Lenny N, Yeoh EJ, et al. (2002) Expression of a conditional AML1-ETO oncogene bypasses embryonic lethality and establishes a murine model of human t(8;21) acute myeloid leukemia. Cancer Cell 1: 63-74.

190. Xiao ZS, Hjelmeland AB, Quarles LD (2004) Selective deficiency of the "bone-related" Runx2-II unexpectedly preserves osteoblast-mediated skeletogenesis. J Biol Chem 279: 20307-20313.

191. Kuo YH, Zaidi SK, Gornostaeva S, Komori T, Stein GS, et al. (2009) Runx2 induces acute myeloid leukemia in cooperation with Cbfbeta-SMMHC in mice. Blood 113: 3323-3332.

192. Muthusamy N, Barton K, Leiden JM (1995) Defective activation and survival of T cells lacking the Ets-1 transcription factor. Nature 377: 639-642.

193. Ayadi A, Zheng H, Sobieszczuk P, Buchwalter G, Moerman P, et al. (2001) Net-targeted mutant mice develop a vascular phenotype and up-regulate egr-1. Embo J 20: 5139-5152.

194. Zhou X, Benson KF, Ashar HR, Chada K (1995) Mutation responsible for the mouse pygmy phenotype in the developmentally regulated factor HMGI-C. Nature 376: 771-774.

195. Monzen K, Ito Y, Naito AT, Kasai H, Hiroi Y, et al. (2008) A crucial role of a high mobility group protein HMGA2 in cardiogenesis. Nat Cell Biol 10: 567-574.

196. Shamblott MJ, Bugg EM, Lawler AM, Gearhart JD (2002) Craniofacial abnormalities resulting from targeted disruption of the murine Sim2 gene. Dev Dyn 224: 373-380.

197. Goshu E, Jin H, Fasnacht R, Sepenski M, Michaud JL, et al. (2002) Sim2 mutants have developmental defects not overlapping with those of Sim1 mutants. Mol Cell Biol 22: 4147-4157.

198. Swiatek PJ, Gridley T (1993) Perinatal lethality and defects in hindbrain development in mice homozygous for a targeted mutation of the zinc finger gene Krox20. Genes Dev 7: 2071-2084.

199. Singh MK, Petry M, Haenig B, Lescher B, Leitges M, et al. (2005) The T-box transcription factor Tbx15 is required for skeletal development. Mech Dev 122: 131-144.

200. Karreth F, Hoebertz A, Scheuch H, Eferl R, Wagner EF (2004) The AP1 transcription factor Fra2 is required for efficient cartilage development. Development 131: 5717-5725.

201. Aruga J, Minowa O, Yaginuma H, Kuno J, Nagai T, et al. (1998) Mouse Zic1 is involved in cerebellar development. J Neurosci 18: 284-293.

202. Hayashi H, Kume T (2008) Forkhead transcription factors regulate expression of the chemokine receptor CXCR4 in endothelial cells and CXCL12-induced cell migration. Biochem Biophys Res Commun 367: 584-589.

203. Seo S, Fujita H, Nakano A, Kang M, Duarte A, et al. (2006) The forkhead transcription factors, Foxc1 and Foxc2, are required for arterial specification and lymphatic sprouting during vascular development. Dev Biol 294: 458-470.

204. Mattiske D, Kume T, Hogan BL (2006) The mouse forkhead gene Foxc1 is required for primordial germ cell migration and antral follicle development. Dev Biol 290: 447-458.

205. Iida K, Koseki H, Kakinuma H, Kato N, Mizutani-Koseki Y, et al. (1997) Essential roles of the winged helix transcription factor MFH-1 in aortic arch patterning and skeletogenesis. Development 124: 4627-4638.

206. Pereira FA, Qiu Y, Zhou G, Tsai MJ, Tsai SY (1999) The orphan nuclear receptor COUP-TFII is required for angiogenesis and heart development. Genes Dev 13: 1037-1049.

207. Chen WY, Wang DH, Yen RC, Luo J, Gu W, et al. (2005) Tumor suppressor HIC1 directly regulates SIRT1 to modulate p53-dependent DNA-damage responses. Cell 123: 437-448.

208. Hoyt PR, Bartholomew C, Davis AJ, Yutzey K, Gamer LW, et al. (1997) The Evi1 proto-oncogene is required at midgestation for neural, heart, and paraxial mesenchyme development. Mech Dev 65: 55-70.

209. Kim JI, Li T, Ho IC, Grusby MJ, Glimcher LH (1999) Requirement for the c-Maf transcription factor in crystallin gene regulation and lens development. Proc Natl Acad Sci U S A 96: 3781-3785.

210. MacLean HE, Kim JI, Glimcher MJ, Wang J, Kronenberg HM, et al. (2003) Absence of transcription factor c-maf causes abnormal terminal differentiation of hypertrophic chondrocytes during endochondral bone development. Dev Biol 262: 51-63.

211. Tevosian SG, Deconinck AE, Tanaka M, Schinke M, Litovsky SH, et al. (2000) FOG-2, a cofactor for GATA transcription factors, is essential for heart morphogenesis and development of coronary vessels from epicardium. Cell 101: 729-739.

212. Svensson EC, Huggins GS, Lin H, Clendenin C, Jiang F, et al. (2000) A syndrome of tricuspid atresia in mice with a targeted mutation of the gene encoding Fog-2. Nat Genet 25: 353-356.

213. Steele-Perkins G, Plachez C, Butz KG, Yang G, Bachurski CJ, et al. (2005) The transcription factor gene Nfib is essential for both lung maturation and brain development. Mol Cell Biol 25: 685-698.

214. Driller K, Pagenstecher A, Uhl M, Omran H, Berlis A, et al. (2007) Nuclear factor I X deficiency causes brain malformation and severe skeletal defects. Mol Cell Biol 27: 3855-3867.

215. das Neves L, Duchala CS, Tolentino-Silva F, Haxhiu MA, Colmenares C, et al. (1999) Disruption of the murine nuclear factor I-A gene (Nfia) results in perinatal lethality, hydrocephalus, and agenesis of the corpus callosum. Proc Natl Acad Sci U S A 96: 11946-11951.

216. Briegel KJ, Joyner AL (2001) Identification and characterization of Lbh, a novel conserved nuclear protein expressed during early limb and heart development. Dev Biol 233: 291-304.

217. Briegel KJ, Baldwin HS, Epstein JA, Joyner AL (2005) Congenital heart disease reminiscent of partial trisomy 2p syndrome in mice transgenic for the transcription factor Lbh. Development 132: 3305-3316.

218. Barash IA, Bang ML, Mathew L, Greaser ML, Chen J, et al. (2007) Structural and regulatory roles of muscle ankyrin repeat protein family in skeletal muscle. Am J Physiol Cell Physiol 293: C218-227.

219. Carver EA, Jiang R, Lan Y, Oram KF, Gridley T (2001) The mouse snail gene encodes a key regulator of the epithelial-mesenchymal transition. Mol Cell Biol 21: 8184-8188.

220. Murray SA, Carver EA, Gridley T (2006) Generation of a Snail1 (Snai1) conditional null allele. Genesis 44: 7-11.

221. Rukstalis JM, Habener JF (2007) Snail2, a mediator of epithelial-mesenchymal transitions, expressed in progenitor cells of the developing endocrine pancreas. Gene Expr Patterns 7: 471-479.

222. Labalette C, Nouet Y, Sobczak-Thepot J, Armengol C, Levillayer F, et al. (2008) The LIM-only protein FHL2 regulates cyclin D1 expression and cell proliferation. J Biol Chem 283: 15201-15208.

223. Kong Y, Shelton JM, Rothermel B, Li X, Richardson JA, et al. (2001) Cardiac-specific LIM protein FHL2 modifies the hypertrophic response to beta-adrenergic stimulation. Circulation 103: 2731-2738.

224. Saether T, Berge T, Ledsaak M, Matre V, Alm-Kristiansen AH, et al. (2007) The chromatin remodeling factor Mi-2alpha acts as a novel co-activator for human c-Myb. J Biol Chem 282: 13994-14005.

225. Paul S, Lombroso PJ (2003) Receptor and nonreceptor protein tyrosine phosphatases in the nervous system. Cell Mol Life Sci 60: 2465-2482.

226. Harroch S, Palmeri M, Rosenbluth J, Custer A, Okigaki M, et al. (2000) No obvious abnormality in mice deficient in receptor protein tyrosine phosphatase beta. Mol Cell Biol 20: 7706-7715.

227. Ceschin DG, Sanchez MC, Chiabrando GA (2009) Insulin induces the low density lipoprotein receptor-related protein 1 (LRP1) degradation by the proteasomal system in J774 macrophage-derived cells. J Cell Biochem 106: 372-380.

228. Herz J, Clouthier DE, Hammer RE (1992) LDL receptor-related protein internalizes and degrades uPA-PAI-1 complexes and is essential for embryo implantation. Cell 71: 411-421.

229. Zheng H, Jiang M, Trumbauer ME, Sirinathsinghji DJ, Hopkins R, et al. (1995) beta-Amyloid precursor protein-deficient mice show reactive gliosis and decreased locomotor activity. Cell 81: 525-531.

230. Rothe J, Lesslauer W, Lotscher H, Lang Y, Koebel P, et al. (1993) Mice lacking the tumour necrosis factor receptor 1 are resistant to TNF-mediated toxicity but highly susceptible to infection by Listeria monocytogenes. Nature 364: 798-802.

231. Pfeffer K, Matsuyama T, Kundig TM, Wakeham A, Kishihara K, et al. (1993) Mice deficient for the 55 kd tumor necrosis factor receptor are resistant to endotoxic shock, yet succumb to L. monocytogenes infection. Cell 73: 457-467.

232. Xanthoulea S, Pasparakis M, Kousteni S, Brakebusch C, Wallach D, et al. (2004) Tumor necrosis factor (TNF) receptor shedding controls thresholds of innate immune activation that balance opposing TNF functions in infectious and inflammatory diseases. J Exp Med 200: 367-376.

233. Evers MR, Salmen B, Bukalo O, Rollenhagen A, Bosl MR, et al. (2002) Impairment of L-type Ca2+ channel-dependent forms of hippocampal synaptic plasticity in mice deficient in the extracellular matrix glycoprotein tenascin-C. J Neurosci 22: 7177-7194.

234. Ohtori S, Isogai E, Hasue F, Ozaki T, Nakamura Y, et al. (2004) Reduced inflammatory pain in mice deficient in the differential screening-selected gene aberrative in neuroblastoma. Mol Cell Neurosci 25: 504-514.
