# Supplementary material for: Analysis of Human and Mouse Reprogramming of Somatic Cells to Induced Pluripotent Stem Cells. What Is in the Plate?
Source: PLoS One. 2010 Sep 17;5(9):e12664. doi: 10.1371/journal.pone.0012664 (PMC2941458; doi:10.1371/journal.pone.0012664)

Figure S1. Schematic representation of the strategy used to reconstruct human and mouse networks of genes that are either up-regulated or down-regulated in reprogramming

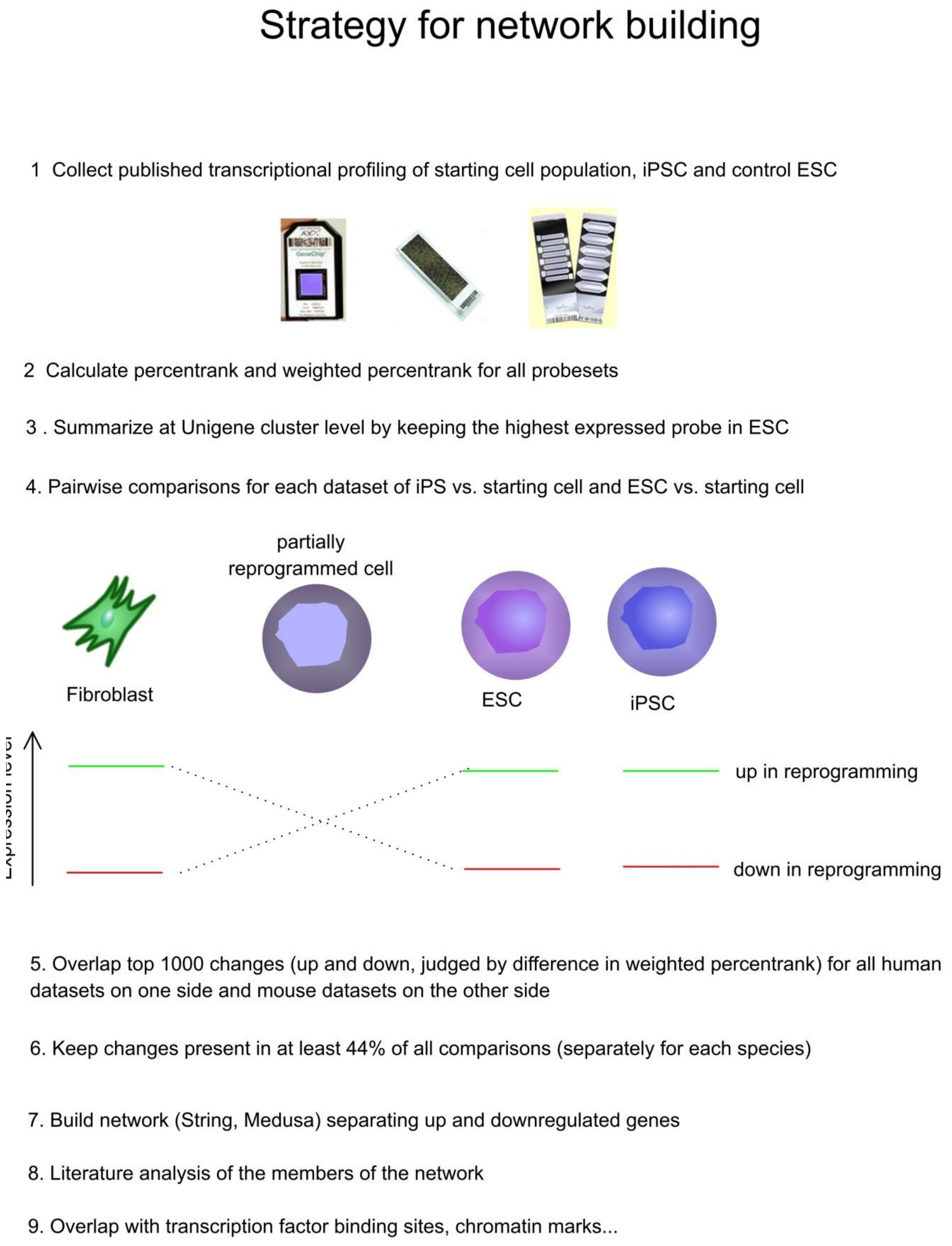

Supplement: Figure S1 — Schematic representation of the strategy used to reconstruct human and mouse networks of genes that are either up-regulated or down-regulated in reprogramming. (0.23 MB PDF) [file pone.0012664.s002.pdf]
