# Supplementary material for: Analysis of Human and Mouse Reprogramming of Somatic Cells to Induced Pluripotent Stem Cells. What Is in the Plate?
Source: PLoS One. 2010 Sep 17;5(9):e12664. doi: 10.1371/journal.pone.0012664 (PMC2941458; doi:10.1371/journal.pone.0012664)

Figure S2. Analysis of bivalent domain-containing genes

In order to evaluate the expression of genes containing bivalent domains, and thereby the quality of the reprogrammed iPSCs compared to ESCs, we have used the overlapping set of genes from 3 genome-wide characterizations of bivalent domain containing genes (see figures below for the human and mouse datasets) which we consider being a high confidence set.

The Venn diagram shows the overlap of genes containing bivalent domains in human ESCs between three datasets. The gene is defined as the Ensembl ID.

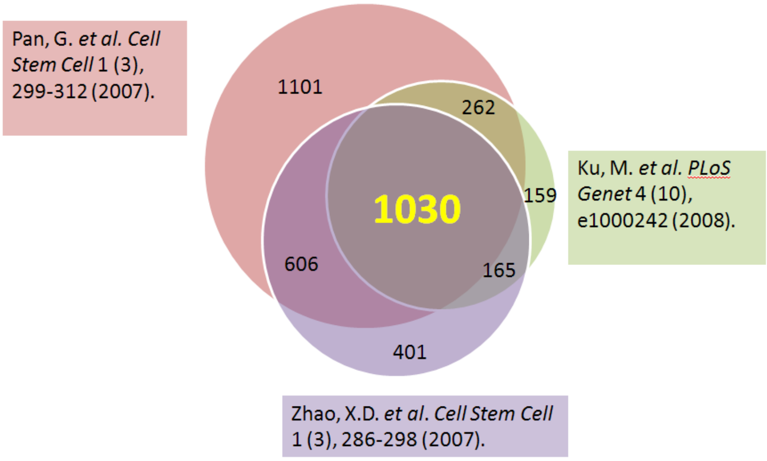

The Venn diagram shows the overlap of genes containing bivalent domains in mouse ESCs between three datasets. The gene ID is defined as the Unigene cluster ID.

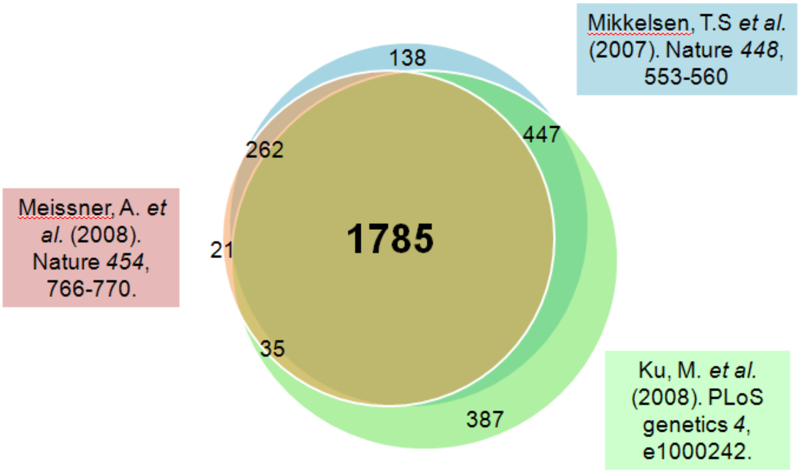

Supplement: Figure S2 — In order to evaluate the expression of genes containing bivalent domains, and thereby the quality of the reprogrammed iPSCs compared to ESCs, we have used the overlapping set of genes from 3 genome-wide characterizations of bivalent domain containing genes (see figures below for the human and mouse datasets) which we consider being a high confidence set. (0.10 MB PDF) [file pone.0012664.s003.pdf]
