# Supplementary material for: Analysis of Human and Mouse Reprogramming of Somatic Cells to Induced Pluripotent Stem Cells. What Is in the Plate?
Source: PLoS One. 2010 Sep 17;5(9):e12664. doi: 10.1371/journal.pone.0012664 (PMC2941458; doi:10.1371/journal.pone.0012664)
Supplement: Figure S3 — Correlation coefficients of different samples and experiments on the profiles of 316 bivalent-domain-containing genes in Human. It is important to note that the profile hasn't been always done on the same platform, which explains why the correlation inter-experiments is sometimes not good. (0.14 MB PDF) [file pone.0012664.s004.pdf]

Figure S3. Correlation coefficients of different samples and experiments on the profiles of 316 bivalent-domain-containing genes in Human.

It is important to note that the profile hasn't been always done on the same platform, which explains why the correlation inter-experiments is sometimes not good.

	Zhao et al.average PR ES	Zhao et al.average PR iPS	Zhao et al.average PR pre-iPS	Zhao et al.average PR fib	Maherali et al.average PR ES	Maherali et al.average PR iPS round 1	Maherali et al.average PR iPS round 2	Maherali et al.average PR fib	Yu et al.average ES	Yu et al.average fib	Yu et al.average iPS	Park et al. average PR ES	Park et al. average PR iPS	Park et al. average PR fib	Yu et al. average PR ES	Yu et al. PR fib	Yu et al. average PR iPS	Ebert et al.average ES	Ebert et al.average iPS
Zhao et al.average PR ES	1.00	0.88	0.50	0.31	0.20	0.15	0.15	-0.03	0.37	0.02	0.36	0.14	0.20	0.00	0.26	-0.03	0.27	0.25	0.26
Zhao et al.average PR iPS	0.88	1.00	0.63	0.44	0.35	0.34	0.34	0.14	0.41	0.17	0.42	0.32	0.34	0.16	0.34	0.14	0.35	0.32	0.33
Zhao et al.average PR pre-iPS	0.50	0.63	1.00	0.52	0.23	0.24	0.24	0.25	0.26	0.24	0.26	0.21	0.18	0.24	0.22	0.24	0.22	0.21	0.21
Zhao et al.average PR fib	0.31	0.44	0.52	1.00	0.24	0.26	0.28	0.56	0.20	0.55	0.21	0.38	0.30	0.59	0.19	0.52	0.21	0.19	0.21
Maherali et al.average PR ES	0.20	0.35	0.23	0.24	1.00	0.96	0.97	0.69	0.62	0.46	0.62	0.84	0.89	0.65	0.81	0.65	0.81	0.80	0.80
Maherali et al.average PR iPS round 1	0.15	0.34	0.24	0.26	0.96	1.00	0.98	0.70	0.56	0.48	0.56	0.85	0.86	0.65	0.75	0.66	0.76	0.74	0.75
Maherali et al.average PR iPS round 2	0.15	0.34	0.24	0.28	0.97	0.98	1.00	0.71	0.57	0.50	0.58	0.84	0.87	0.67	0.75	0.68	0.76	0.75	0.75
Maherali et al.average PR fib	-0.03	0.14	0.25	0.56	0.69	0.70	0.71	1.00	0.34	0.67	0.35	0.71	0.67	0.85	0.52	0.87	0.53	0.53	0.55
Yu et al.average ES	0.37	0.41	0.26	0.20	0.62	0.56	0.57	0.34	1.00	0.50	0.97	0.54	0.61	0.40	0.71	0.37	0.70	0.69	0.66
Yu et al.average fib	0.02	0.17	0.24	0.55	0.46	0.48	0.50	0.67	0.50	1.00	0.52	0.57	0.52	0.72	0.40	0.65	0.40	0.38	0.37
Yu et al.average iPS	0.36	0.42	0.26	0.21	0.62	0.56	0.58	0.35	0.97	0.52	1.00	0.56	0.62	0.41	0.71	0.38	0.70	0.69	0.67
Park et al. average PR ES	0.14	0.32	0.21	0.38	0.84	0.85	0.84	0.71	0.54	0.57	0.56	1.00	0.92	0.80	0.71	0.69	0.72	0.71	0.71
Park et al. average PR iPS	0.20	0.34	0.18	0.30	0.89	0.86	0.87	0.67	0.61	0.52	0.62	0.92	1.00	0.75	0.79	0.64	0.80	0.79	0.80
Park et al. average PR fib	0.00	0.16	0.24	0.59	0.65	0.65	0.67	0.85	0.40	0.72	0.41	0.80	0.75	1.00	0.53	0.80	0.54	0.53	0.53
Yu et al. average PR ES	0.26	0.34	0.22	0.19	0.81	0.75	0.75	0.52	0.71	0.40	0.71	0.71	0.79	0.53	1.00	0.61	0.98	0.95	0.94
Yu et al. PR fib	-0.03	0.14	0.24	0.52	0.65	0.66	0.68	0.87	0.37	0.65	0.38	0.69	0.64	0.80	0.61	1.00	0.62	0.61	0.61
Yu et al. average PR iPS	0.27	0.35	0.22	0.21	0.81	0.76	0.76	0.53	0.70	0.40	0.70	0.72	0.80	0.54	0.98	0.62	1.00	0.92	0.92
Ebert et al.average ES	0.25	0.32	0.21	0.19	0.80	0.74	0.75	0.53	0.69	0.38	0.69	0.71	0.79	0.53	0.95	0.61	0.92	1.00	0.99
Ebert et al.average iPS	0.26	0.33	0.21	0.21	0.80	0.75	0.75	0.55	0.66	0.37	0.67	0.71	0.80	0.53	0.94	0.61	0.92	0.99	1.00
Ebert et al.average fib	0.00	0.13	0.22	0.55	0.64	0.63	0.65	0.80	0.40	0.63	0.40	0.69	0.67	0.80	0.60	0.89	0.61	0.65	0.64
Takahashi et al. rank in ES9	0.28	0.35	0.21	0.17	0.59	0.53	0.55	0.34	0.59	0.29	0.61	0.52	0.57	0.37	0.61	0.35	0.61	0.62	0.61
Takahashi et al. rank in human iPS	0.29	0.34	0.20	0.21	0.58	0.52	0.52	0.36	0.60	0.34	0.63	0.54	0.62	0.41	0.64	0.37	0.66	0.64	0.65
Takahashi et al. rank in fibroblast	-0.02	0.11	0.26	0.60	0.39	0.38	0.41	0.68	0.29	0.62	0.30	0.51	0.43	0.69	0.34	0.65	0.36	0.37	0.36
Lowry et al. average ES	0.19	0.33	0.22	0.28	0.87	0.83	0.83	0.65	0.60	0.49	0.60	0.86	0.92	0.69	0.76	0.63	0.76	0.77	0.77
Lowry et al. average fibroblasts	-0.03	0.15	0.27	0.59	0.62	0.64	0.66	0.87	0.30	0.66	0.31	0.73	0.68	0.86	0.46	0.81	0.46	0.48	0.49
Lowry et al. average iPS	0.07	0.28	0.25	0.38	0.82	0.84	0.84	0.76	0.47	0.59	0.50	0.86	0.85	0.78	0.64	0.71	0.66	0.64	0.65
Lowry et al. average partial ips	0.03	0.20	0.31	0.44	0.74	0.75	0.74	0.75	0.42	0.60	0.43	0.79	0.79	0.78	0.58	0.71	0.59	0.58	0.59
Aasen et al. average ES	0.19	0.30	0.22	0.23	0.80	0.78	0.77	0.56	0.58	0.41	0.57	0.73	0.76	0.55	0.72	0.54	0.73	0.72	0.73
Aasen et al. average fib	-0.06	0.12	0.25	0.56	0.59	0.61	0.61	0.80	0.31	0.63	0.30	0.67	0.61	0.76	0.45	0.76	0.47	0.47	0.48
Aasen et al. average ker	-0.06	0.09	0.22	0.39	0.58	0.60	0.59	0.65	0.30	0.53	0.28	0.59	0.57	0.65	0.45	0.62	0.46	0.45	0.47
Aasen et al. average kips	0.13	0.29	0.22	0.24	0.79	0.80	0.78	0.56	0.52	0.42	0.52	0.74	0.74	0.55	0.67	0.53	0.68	0.66	0.68
Huangfu et al. average fib	0.01	0.15	0.30	0.59	0.44	0.46	0.47	0.73	0.27	0.63	0.28	0.54	0.48	0.70	0.35	0.70	0.37	0.37	0.38
Huangfu et al. average PR ESC	0.22	0.36	0.24	0.13	0.62	0.62	0.63	0.39	0.47	0.33	0.48	0.52	0.54	0.35	0.53	0.39	0.53	0.52	0.52
Huangfu et al. average PR iPS	0.17	0.35	0.23	0.15	0.63	0.64	0.65	0.42	0.44	0.35	0.45	0.54	0.53	0.38	0.51	0.41	0.52	0.49	0.50
Soldner et al. average PR ESC	0.22	0.35	0.23	0.20	0.78	0.76	0.78	0.51	0.37	0.61	0.60	0.78	0.75	0.53	0.55	0.79	0.76	0.79	0.77
Soldner et al. average fib	-0.04	0.09	0.19	0.54	0.58	0.59	0.57	0.75	0.59	0.36	0.35	0.61	0.63	0.75	0.78	0.52	0.51	0.53	0.51
Soldner et al. average PR iPS	0.23	0.35	0.23	0.21	0.74	0.72	0.75	0.49	0.38	0.62	0.60	0.76	0.73	0.52	0.56	0.79	0.76	0.78	0.76
Soldner et al. average PR iPS int	0.24	0.36	0.22	0.21	0.76	0.74	0.77	0.49	0.36	0.60	0.59	0.76	0.72	0.51	0.54	0.77	0.74	0.77	0.75

	Ebert et al.average fib	Takahashi et al. rank in ES9	Takahashi et al. rank in human iPS	Takahashi et al. rank in fibroblast	Lowry et al. average ES	Lowry et al. average fibroblasts	Lowry et al. average iPS	Lowry et al. average partial ips	Aasen et al. average ES	Aasen et al. average fib	Aasen et al. average ker	Aasen et al. average kips	Huangfu et al. average fib	Huangfu et al. average PR ESC	Huangfu et al. average PR iPS	Soldner et al. average PR ESC	Soldner et al. average fib	Soldner et al. average PR iPS excised	Soldner et al. average PR iPS int
Zhao et al.average PR ES	0.00	0.28	0.29	-0.02	0.19	-0.03	0.07	0.03	0.19	-0.06	-0.06	0.13	0.01	0.22	0.17	0.22	-0.04	0.23	0.24
Zhao et al.average PR iPS	0.13	0.35	0.34	0.11	0.33	0.15	0.28	0.20	0.30	0.12	0.09	0.29	0.15	0.36	0.35	0.35	0.09	0.35	0.36
Zhao et al.average PR pre-iPS	0.22	0.21	0.20	0.26	0.22	0.27	0.25	0.31	0.22	0.25	0.22	0.22	0.30	0.24	0.23	0.23	0.19	0.23	0.22
Zhao et al.average PR fib	0.55	0.17	0.21	0.60	0.28	0.59	0.38	0.44	0.23	0.56	0.39	0.24	0.59	0.13	0.15	0.20	0.54	0.21	0.21
Maherali et al.average PR ES	0.64	0.59	0.58	0.39	0.87	0.62	0.82	0.74	0.80	0.59	0.58	0.79	0.44	0.62	0.63	0.78	0.58	0.74	0.76
Maherali et al.average PR iPS round 1	0.63	0.53	0.52	0.38	0.83	0.64	0.84	0.75	0.78	0.61	0.60	0.80	0.46	0.62	0.64	0.76	0.59	0.72	0.74
Maherali et al.average PR iPS round 2	0.65	0.55	0.52	0.41	0.83	0.66	0.84	0.74	0.77	0.61	0.59	0.78	0.47	0.63	0.65	0.78	0.57	0.75	0.77
Maherali et al.average PR fib	0.80	0.34	0.36	0.68	0.65	0.87	0.76	0.75	0.56	0.80	0.65	0.56	0.73	0.39	0.42	0.51	0.75	0.49	0.49
Yu et al.average ES	0.40	0.59	0.60	0.29	0.60	0.30	0.47	0.42	0.58	0.31	0.30	0.52	0.27	0.47	0.44	0.37	0.59	0.38	0.36
Yu et al.average fib	0.63	0.29	0.34	0.62	0.49	0.66	0.59	0.60	0.41	0.63	0.53	0.42	0.63	0.33	0.35	0.61	0.36	0.62	0.60
Yu et al.average iPS	0.40	0.61	0.63	0.30	0.60	0.31	0.50	0.43	0.57	0.30	0.28	0.52	0.28	0.48	0.45	0.60	0.35	0.60	0.59
Park et al. average PR ES	0.69	0.52	0.54	0.51	0.86	0.73	0.86	0.79	0.73	0.67	0.59	0.74	0.54	0.52	0.54	0.78	0.61	0.76	0.76
Park et al. average PR iPS	0.67	0.57	0.62	0.43	0.92	0.68	0.85	0.79	0.76	0.61	0.57	0.74	0.48	0.54	0.53	0.75	0.63	0.73	0.72
Park et al. average PR fib	0.80	0.37	0.41	0.69	0.69	0.86	0.78	0.78	0.55	0.76	0.65	0.55	0.70	0.35	0.38	0.53	0.75	0.52	0.51
Yu et al. average PR ES	0.60	0.61	0.64	0.34	0.76	0.46	0.64	0.58	0.72	0.45	0.45	0.67	0.35	0.53	0.51	0.55	0.78	0.56	0.54
Yu et al. PR fib	0.89	0.35	0.37	0.65	0.63	0.81	0.71	0.71	0.54	0.76	0.62	0.53	0.70	0.39	0.41	0.79	0.52	0.79	0.77
Yu et al. average PR iPS	0.61	0.61	0.66	0.36	0.76	0.46	0.66	0.59	0.73	0.47	0.46	0.68	0.37	0.53	0.52	0.76	0.51	0.76	0.74
Ebert et al.average ES	0.65	0.62	0.64	0.37	0.77	0.48	0.64	0.58	0.72	0.47	0.45	0.66	0.37	0.52	0.49	0.79	0.53	0.78	0.77
Ebert et al.average iPS	0.64	0.61	0.65	0.36	0.77	0.49	0.65	0.59	0.73	0.48	0.47	0.68	0.38	0.52	0.50	0.77	0.51	0.76	0.75
Ebert et al.average fib	1.00	0.37	0.39	0.67	0.62	0.77	0.68	0.71	0.55	0.75	0.62	0.53	0.67	0.34	0.35	0.57	0.85	0.58	0.57
Takahashi et al. rank in ES9	0.37	1.00	0.89	0.51	0.57	0.30	0.49	0.41	0.53	0.30	0.29	0.48	0.28	0.48	0.46	0.56	0.34	0.54	0.53
Takahashi et al. rank in human iPS	0.39	0.89	1.00	0.53	0.58	0.34	0.47	0.44	0.55	0.33	0.33	0.51	0.31	0.45	0.43	0.31	0.62	0.33	0.31
Takahashi et al. rank in fibroblast	0.67	0.51	0.53	1.00	0.41	0.68	0.51	0.56	0.34	0.65	0.50	0.35	0.67	0.23	0.25	0.58	0.31	0.55	0.55
Lowry et al. average ES	0.62	0.57	0.58	0.41	1.00	0.70	0.89	0.81	0.79	0.62	0.58	0.76	0.45	0.52	0.52	0.47	0.72	0.46	0.46
Lowry et al. average fibroblasts	0.77	0.30	0.34	0.68	0.70	1.00	0.80	0.83	0.53	0.80	0.64	0.55	0.71	0.32	0.36	0.77	0.57	0.74	0.74
Lowry et al. average iPS	0.68	0.49	0.47	0.51	0.89	0.80	1.00	0.90	0.71	0.73	0.67	0.74	0.54	0.52	0.56	0.67	0.64	0.64	0.63
Lowry et al. average partial ips	0.71	0.41	0.44	0.56	0.81	0.83	0.90	1.00	0.67	0.74	0.68	0.69	0.58	0.40	0.43	0.61	0.67	0.58	0.58
Aasen et al. average ES	0.55	0.53	0.55	0.34	0.79	0.53	0.71	0.67	1.00	0.70	0.70	0.95	0.38	0.52	0.51	0.72	0.50	0.70	0.70
Aasen et al. average fib	0.75	0.30	0.33	0.65	0.62	0.80	0.73	0.74	0.70	1.00	0.83	0.72	0.68	0.33	0.37	0.46	0.70	0.45	0.44
Aasen et al. average ker	0.62	0.29	0.33	0.50	0.58	0.64	0.67	0.68	0.70	0.83	1.00	0.71	0.50	0.31	0.35	0.45	0.59	0.44	0.43
Aasen et al. average kips	0.53	0.48	0.51	0.35	0.76	0.55	0.74	0.69	0.95	0.72	0.71	1.00	0.42	0.56	0.57	0.69	0.49	0.67	0.67
Huangfu et al. average fib	0.67	0.28	0.31	0.67	0.45	0.71	0.54	0.58	0.38	0.68	0.50	0.42	1.00	0.58	0.60	0.35	0.62	0.36	0.34
Huangfu et al. average PR ESC	0.34	0.48	0.45	0.23	0.52	0.32	0.52	0.40	0.52	0.33	0.31	0.56	0.58	1.00	0.97	0.56	0.32	0.54	0.54
Huangfu et al. average PR iPS	0.35	0.46	0.43	0.25	0.52	0.36	0.56	0.43	0.51	0.37	0.35	0.57	0.60	0.97	1.00	0.53	0.32	0.50	0.50
Soldner et al. average PR ESC	0.57	0.56	0.31	0.58	0.47	0.77	0.67	0.61	0.72	0.46	0.45	0.69	0.35	0.56	0.53	1.00	0.61	0.97	0.97
Soldner et al. average fib	0.85	0.34	0.62	0.31	0.72	0.57	0.64	0.67	0.50	0.70	0.59	0.49	0.62	0.32	0.32	0.61	1.00	0.62	0.60
Soldner et al. average PR iPS	0.58	0.54	0.33	0.55	0.46	0.74	0.64	0.58	0.70	0.45	0.44	0.67	0.36	0.54	0.50	0.97	0.62	1.00	0.97
Soldner et al. average PR iPS int	0.57	0.53	0.31	0.55	0.46	0.74	0.63	0.58	0.70	0.44	0.43	0.67	0.34	0.54	0.50	0.97	0.60	0.97	1.00
