# Supplementary material for: Analysis of Human and Mouse Reprogramming of Somatic Cells to Induced Pluripotent Stem Cells. What Is in the Plate?
Source: PLoS One. 2010 Sep 17;5(9):e12664. doi: 10.1371/journal.pone.0012664 (PMC2941458; doi:10.1371/journal.pone.0012664)
Supplement: Figure S4 — Principal component analyses of Human and Mouse reprogramming datasets show that ESCs and iPSCs usually cluster together, far from the starting somatic cell population. Principal component analysis of genome-wide intensity values (or log of intensity gcrma-normalized) (A) Human datasets (B) Mouse datasets. (0.72 MB PDF) [file pone.0012664.s005.pdf]

Figure S4. Principal component analyses of Human and Mouse reprogramming datasets show that ESCs and iPSCs usually cluster together, far from the starting somatic cell population.

Principal component analysis of genome-wide intensity values (or log of intensity gcrma-normalized) (A) Human datasets (B) Mouse datasets

A- Human datasets

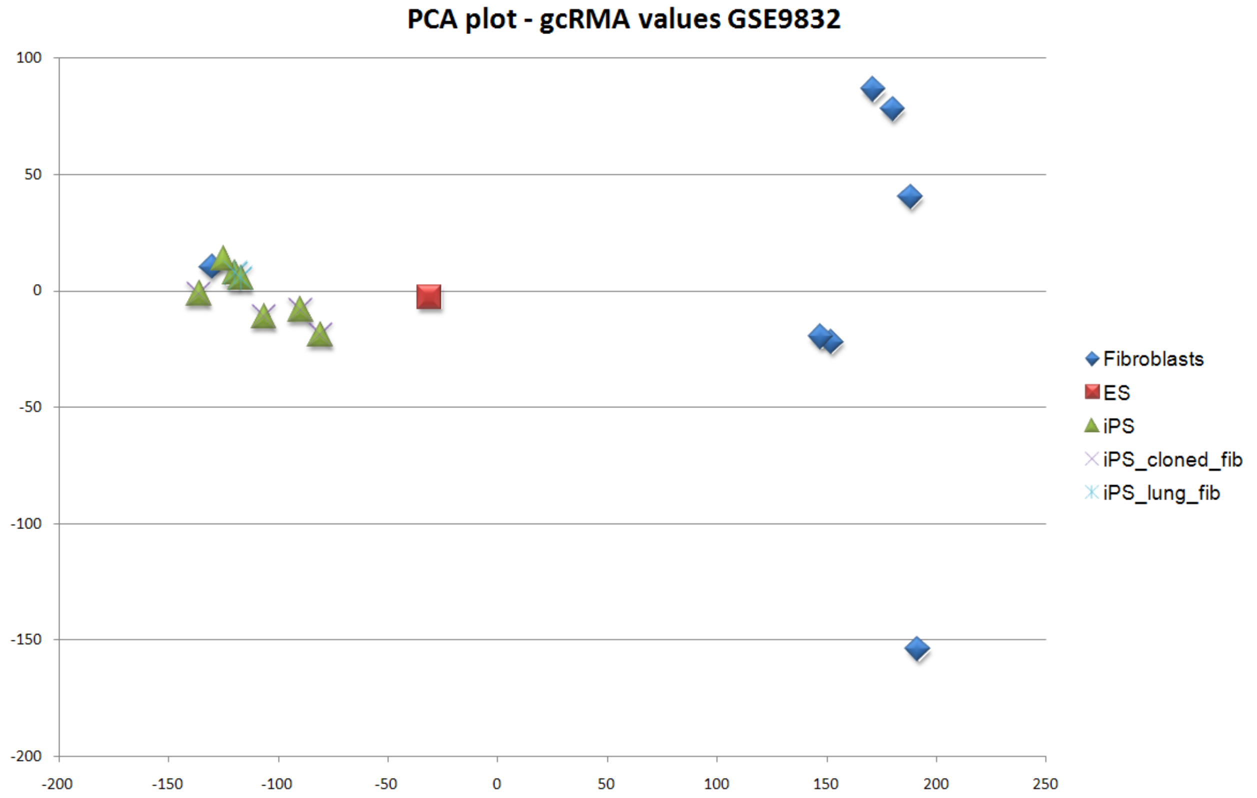

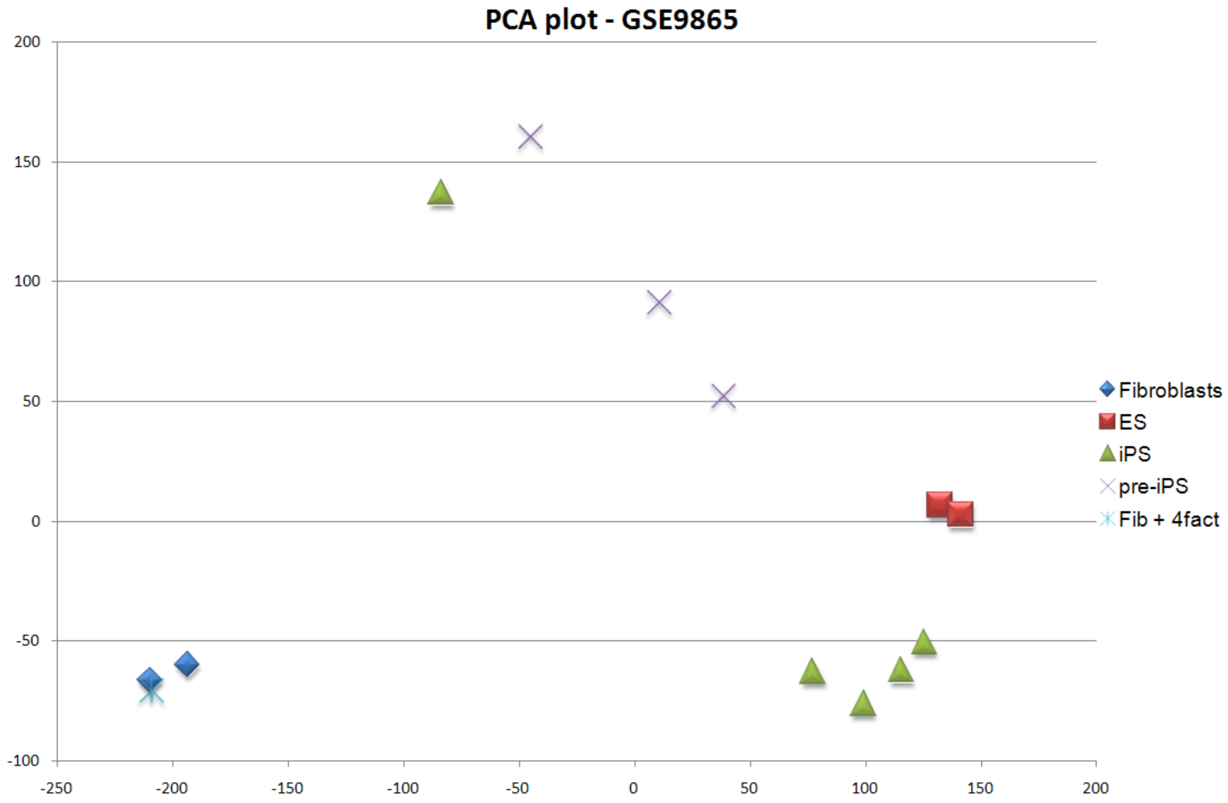
PCA plot - GSE9865
200
150
100
50
0
-50
-100
-250
-200
-150
-100
-50
0
50
100
150
200
Fibroblasts
ES
iPS
pre-iPS
Fib + 4fact

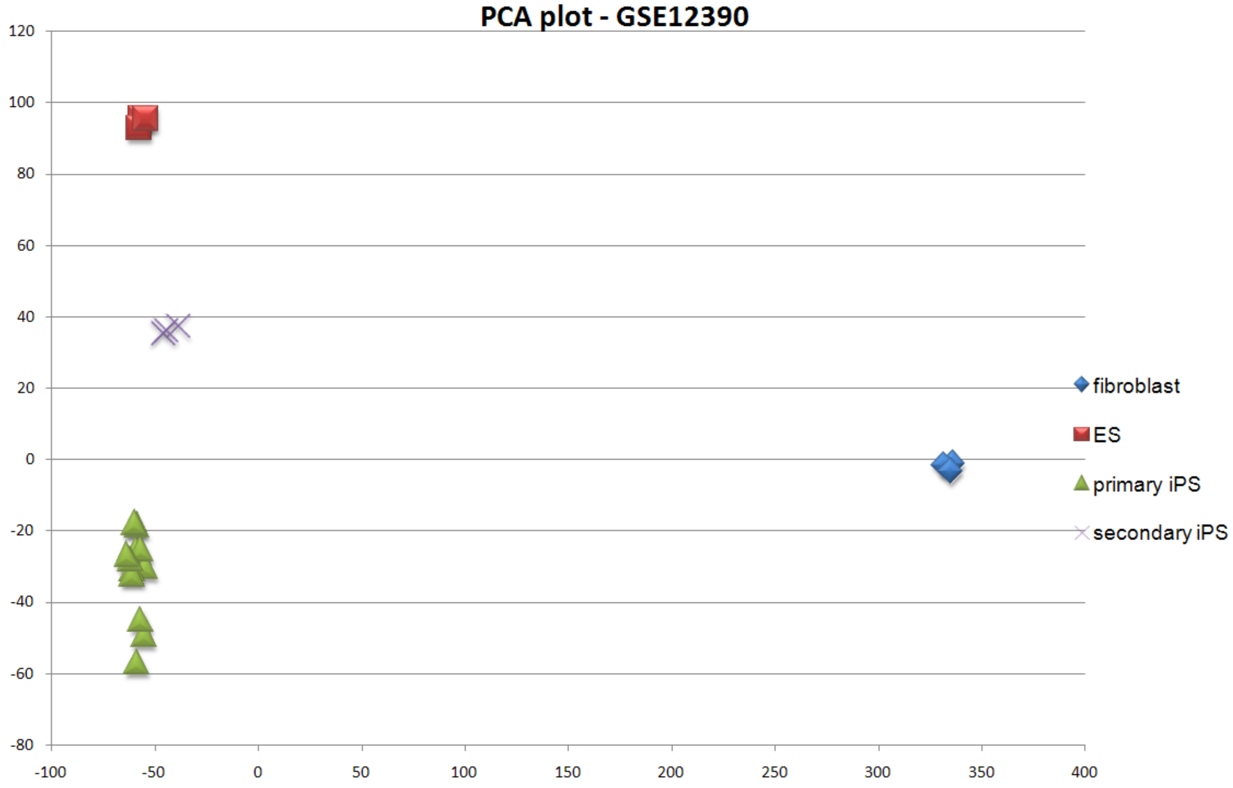
PCA plot - GSE12390
120
100
80
60
40
20
0
-20
-40
-60
-80
-100
-50
0
50
100
150
200
250
300
350
400
fibroblast
ES
primary iPS
secondary iPS

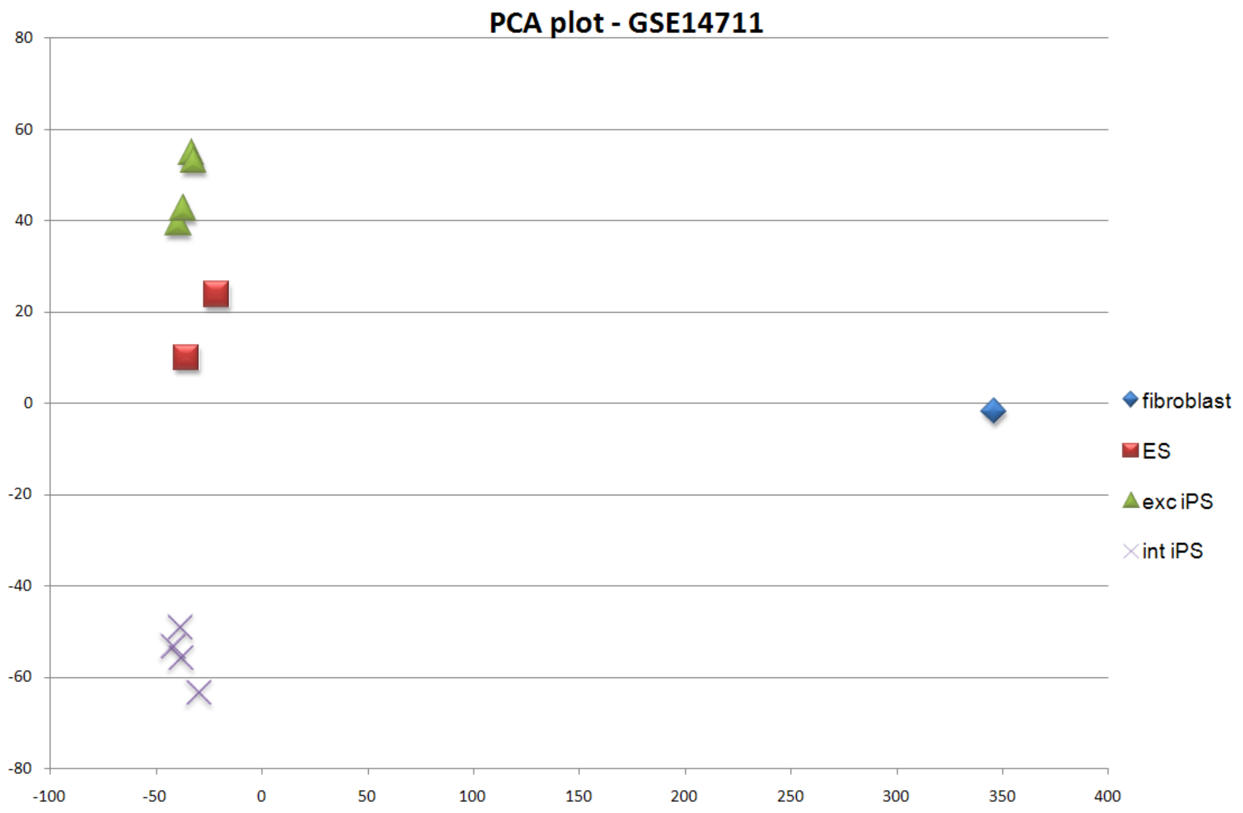
PCA plot - GSE14711
80
60
40
20
0
-20
-40
-60
-80
-100
-50
0
50
100
150
200
250
300
350
400
fibroblast
ES
exc iPS
int iPS

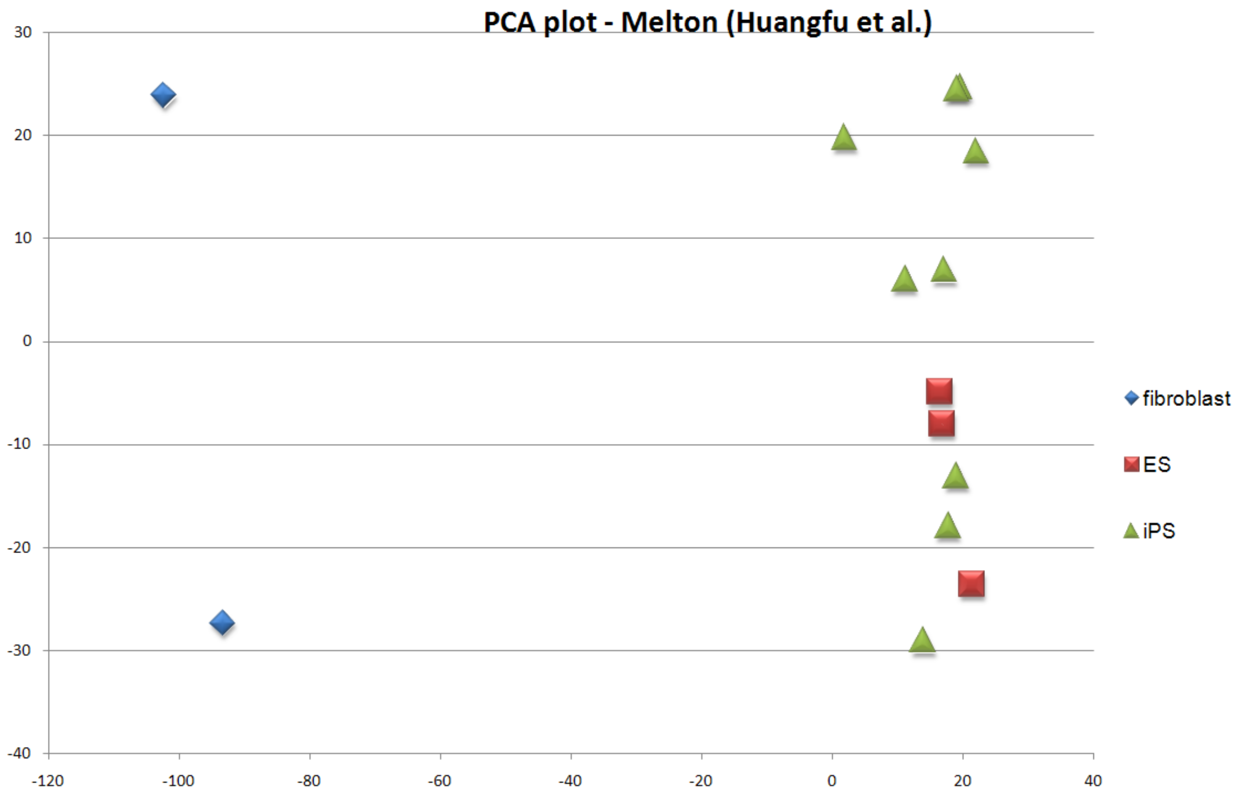
PCA plot - Melton (Huangfu et al.)
30
20
10
0
-10
-20
-30
-40
-120
-100
-80
-60
-40
-20
0
20
40
fibroblast
ES
iPS

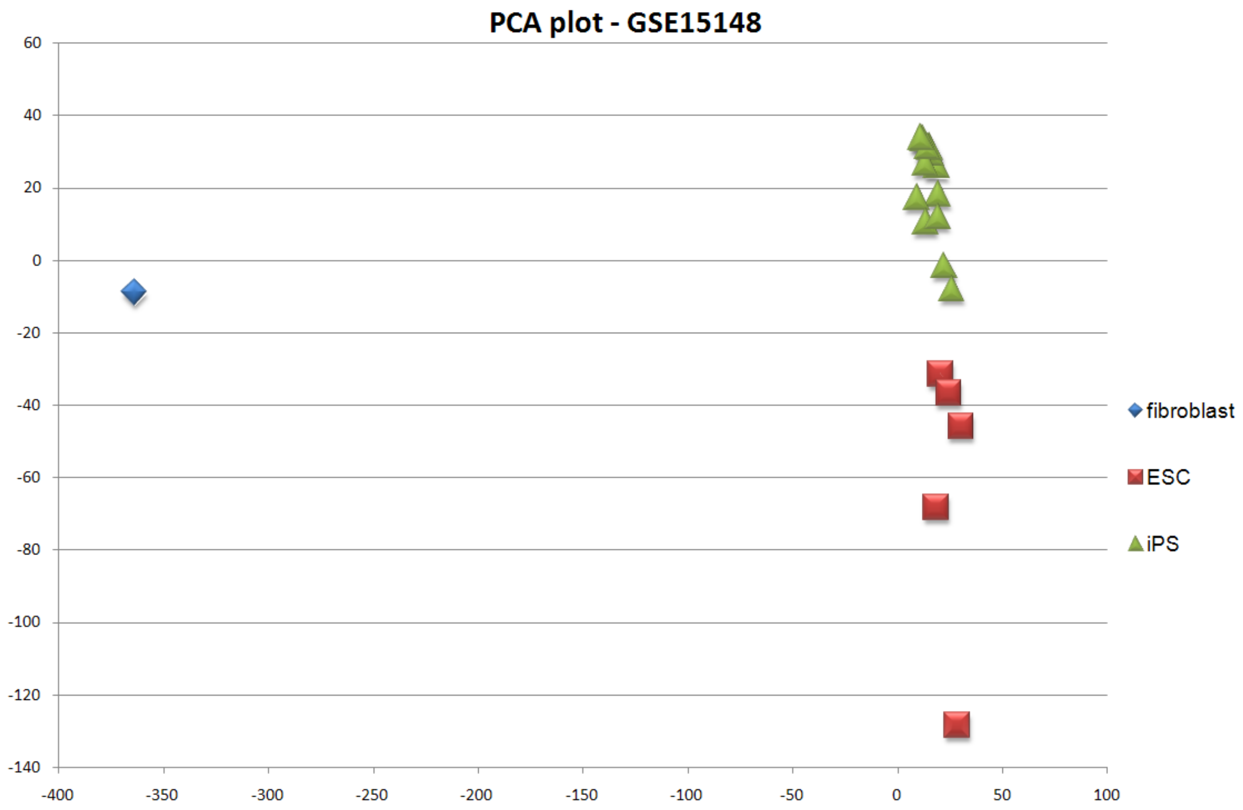
PCA plot - GSE15148
60
40
20
0
-20
-40
-60
-80
-100
-120
-140
-400
-350
-300
-250
-200
-150
-100
-50
0
50
100
fibroblast
ESC
iPS

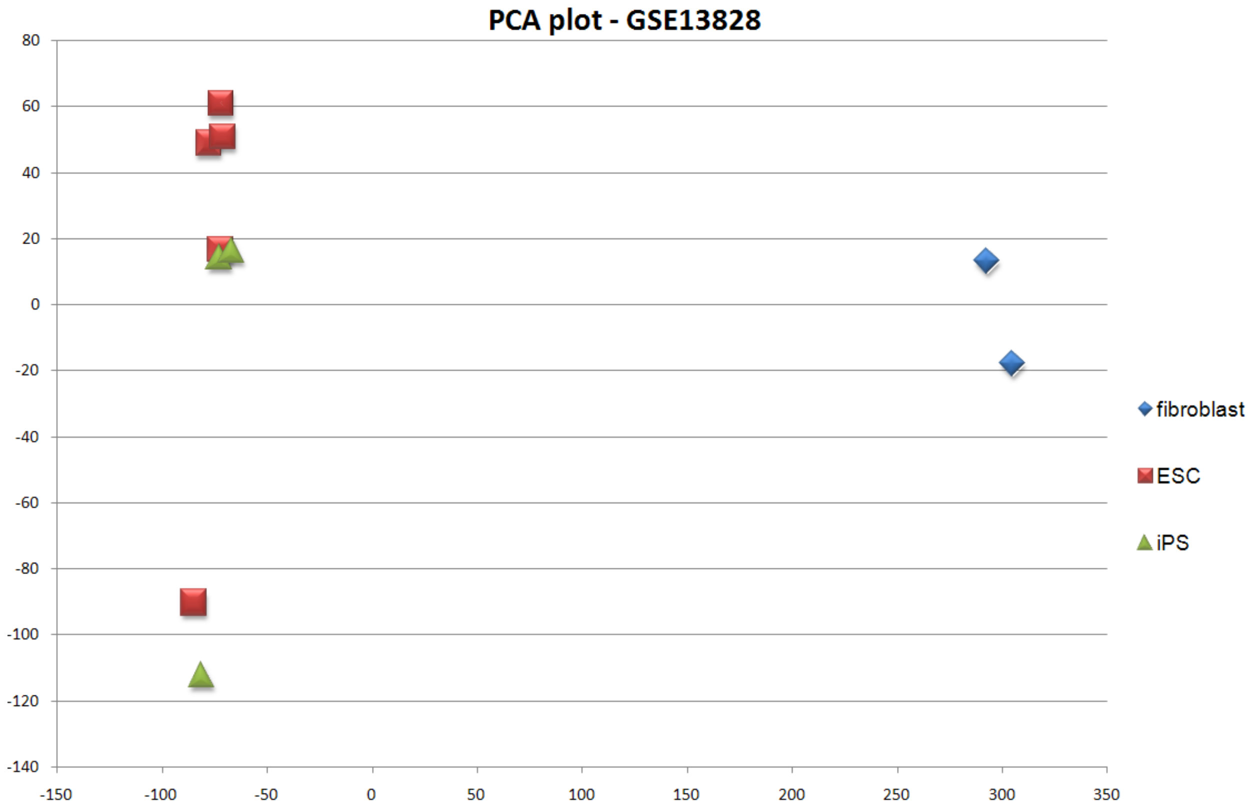
PCA plot - GSE13828
80
60
40
20
0
-20
-40
-60
-80
-100
-120
-140
-150
-100
-50
0
50
100
150
200
250
300
350
fibroblast
ESC
iPS

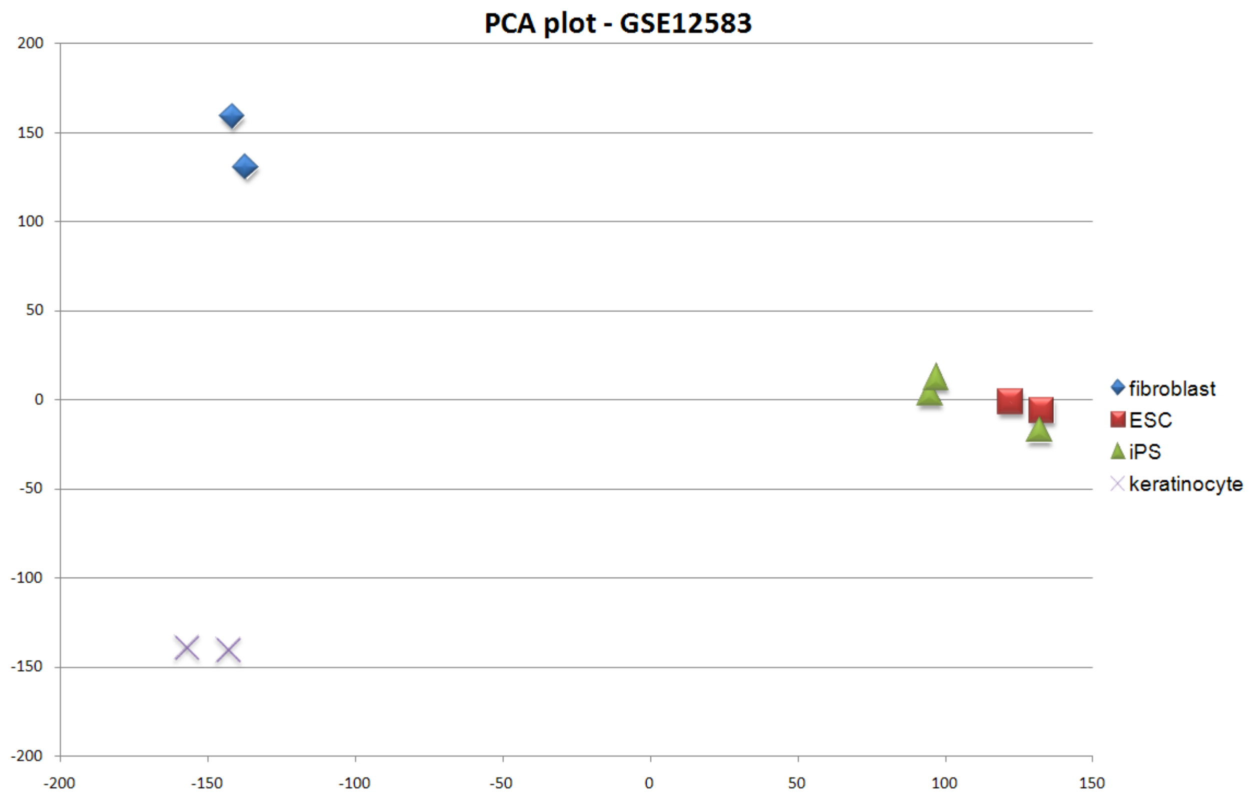
PCA plot - GSE12583
200
150
100
50
0
-50
-100
-150
-200
-200
-150
-100
-50
0
50
100
150
fibroblast
ESC
iPS
keratinocyte

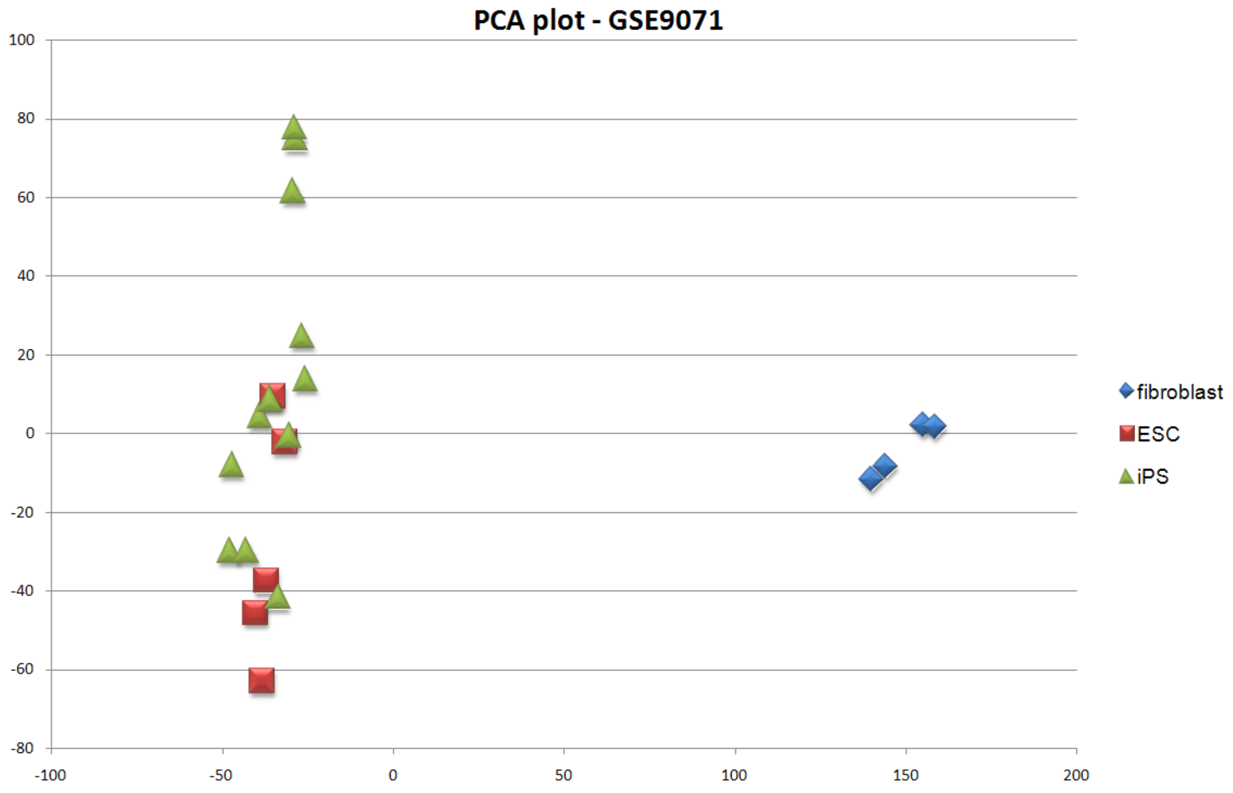
PCA plot - GSE9071
100
80
60
40
20
0
-20
-40
-60
-80
-100
-50
0
50
100
150
200
fibroblast
ESC
iPS

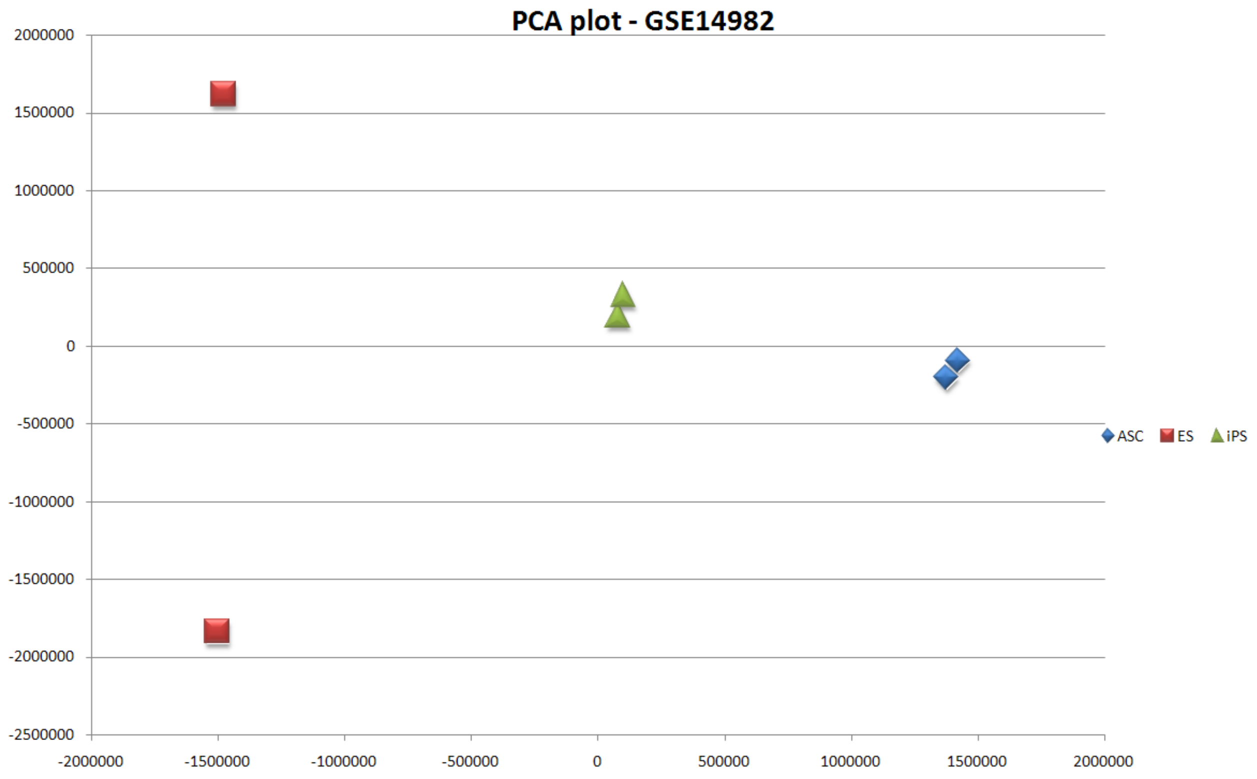
PCA plot - GSE14982
2000000
1500000
1000000
500000
0
-500000
-1000000
-1500000
-2000000
-2500000
-2000000
-1500000
-1000000
-500000
0
500000
1000000
1500000
2000000
ASC
ES
iPS

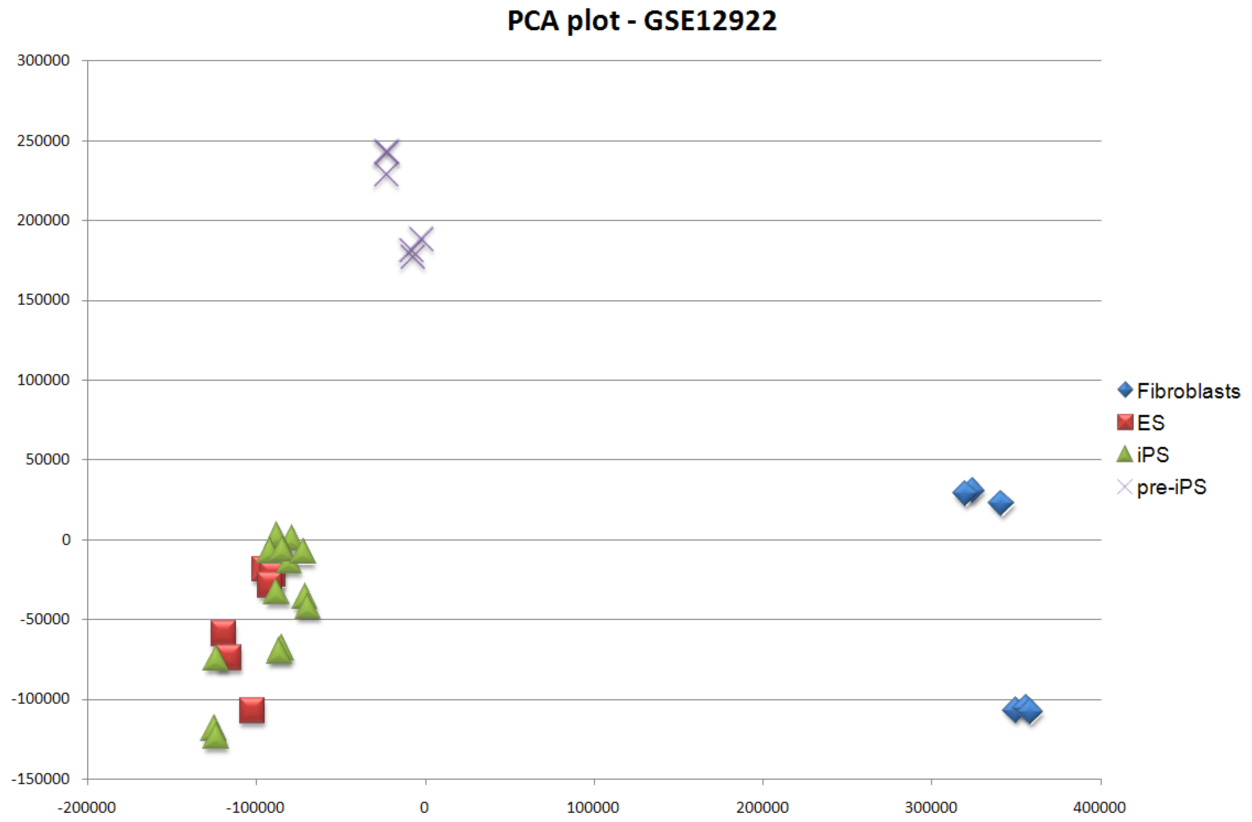
PCA plot - GSE12922
300000
250000
200000
150000
100000
50000
0
-50000
-100000
-150000
-200000
-100000
0
100000
200000
300000
400000
Fibroblasts
ES
iPS
pre-iPS

B- Mouse datasets

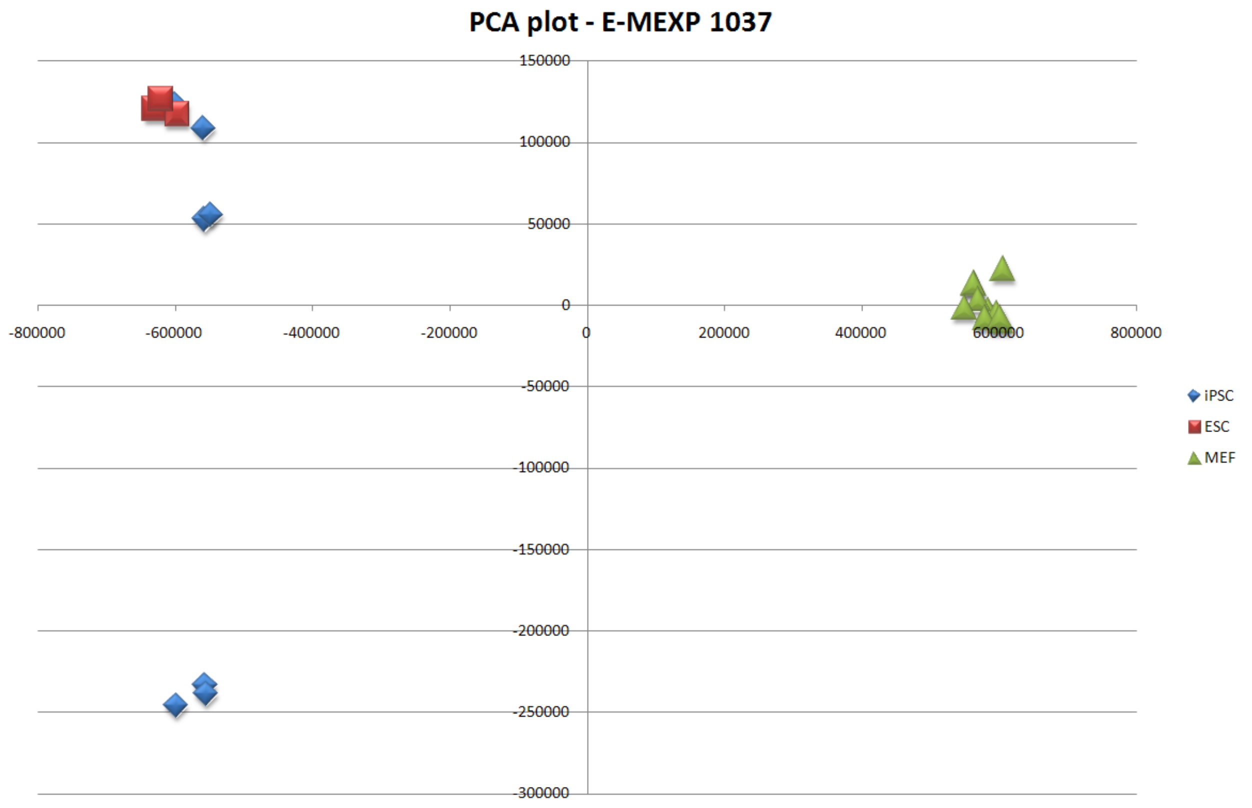

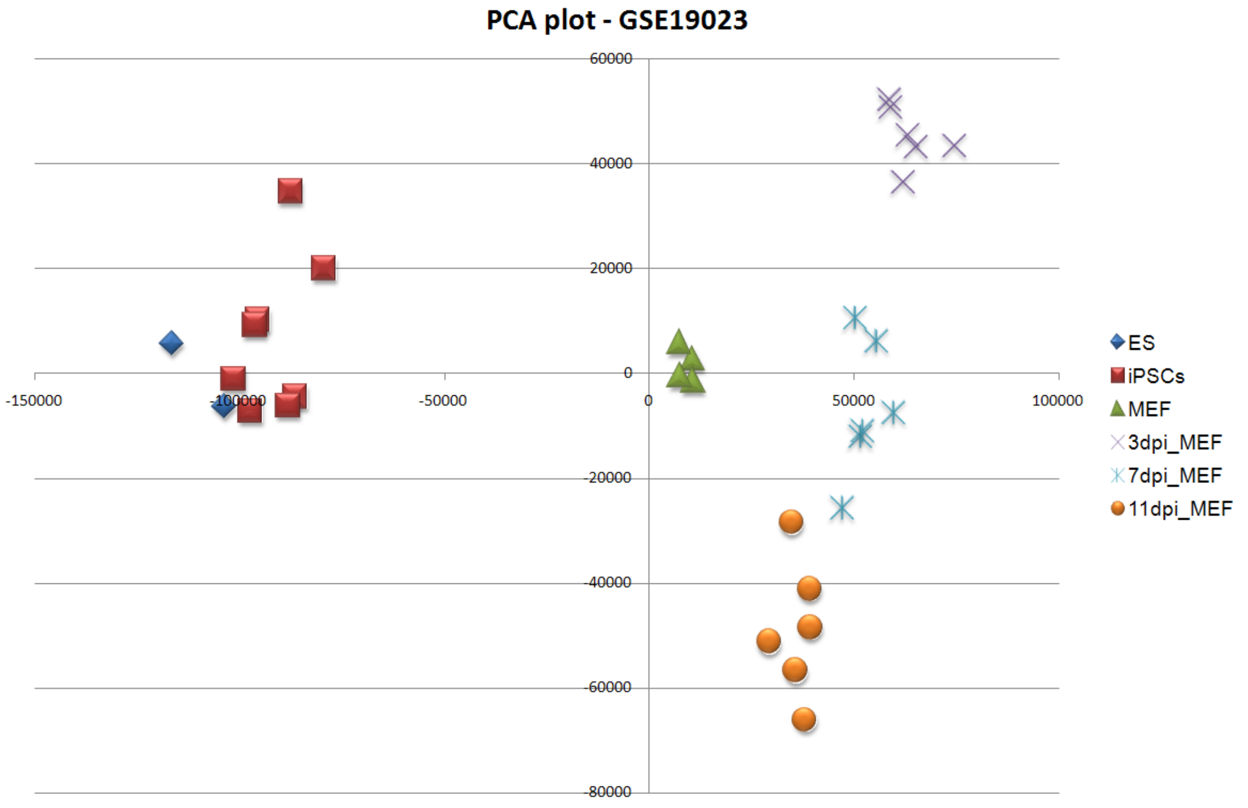

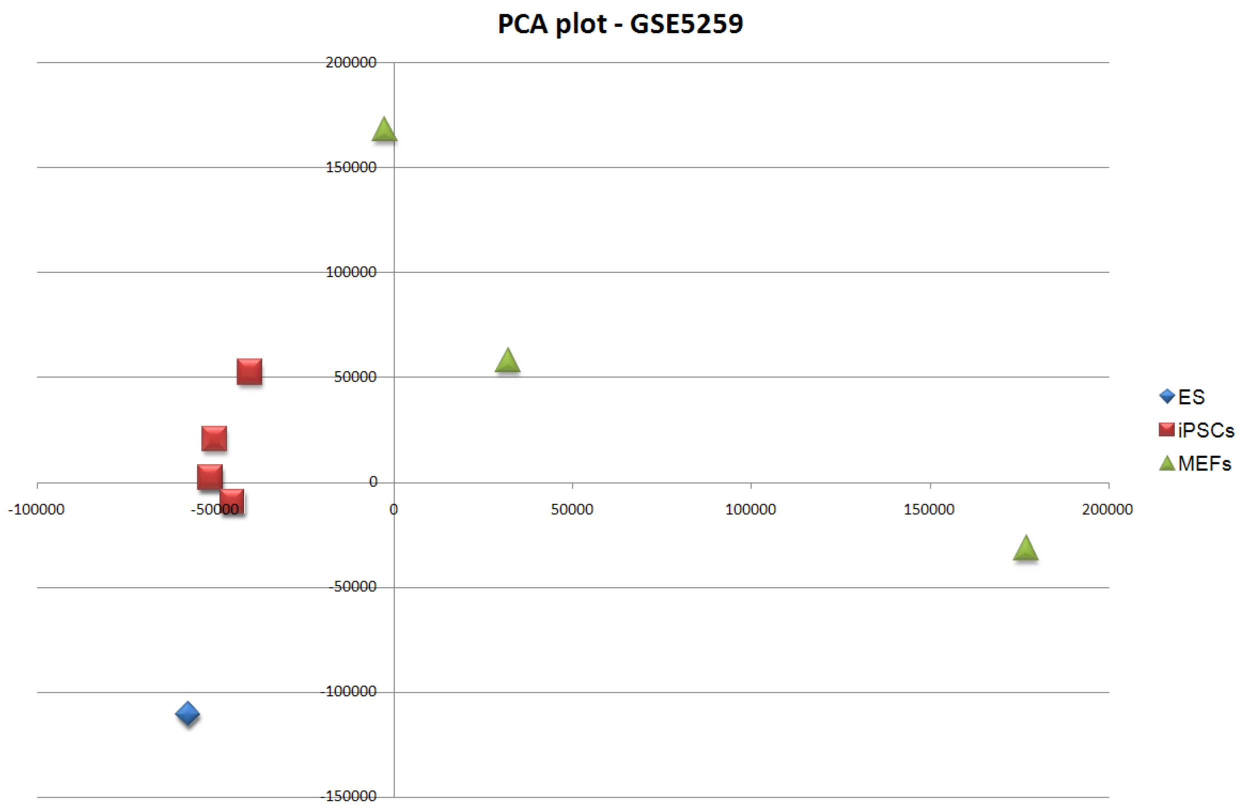
PCA plot - GSE5259
200000
150000
100000
50000
0
-50000
-100000
-150000
-100000
-50000
0
50000
100000
150000
200000
ES
iPSCs
MEFs

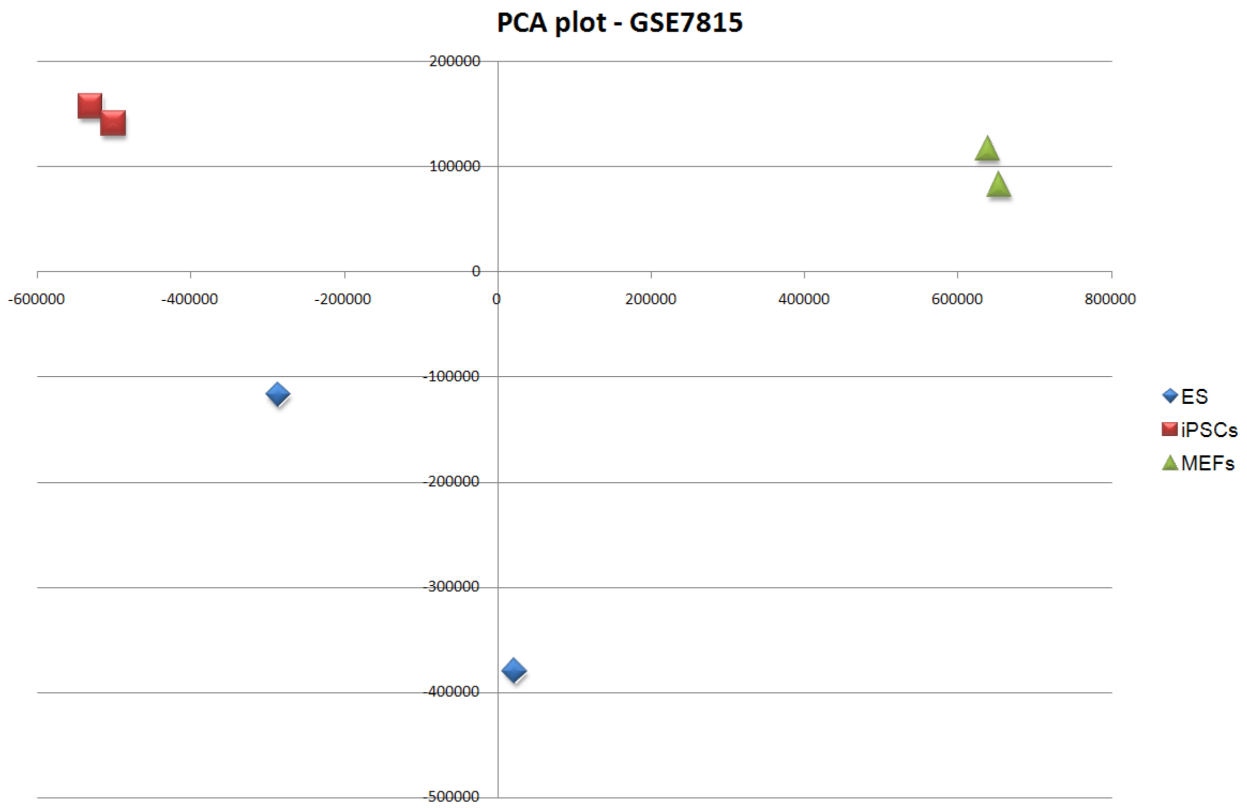
PCA plot - GSE7815
200000
100000
0
-100000
-200000
-300000
-400000
-500000
-600000
-400000
-200000
0
200000
400000
600000
800000
ES
iPSCs
MEFs

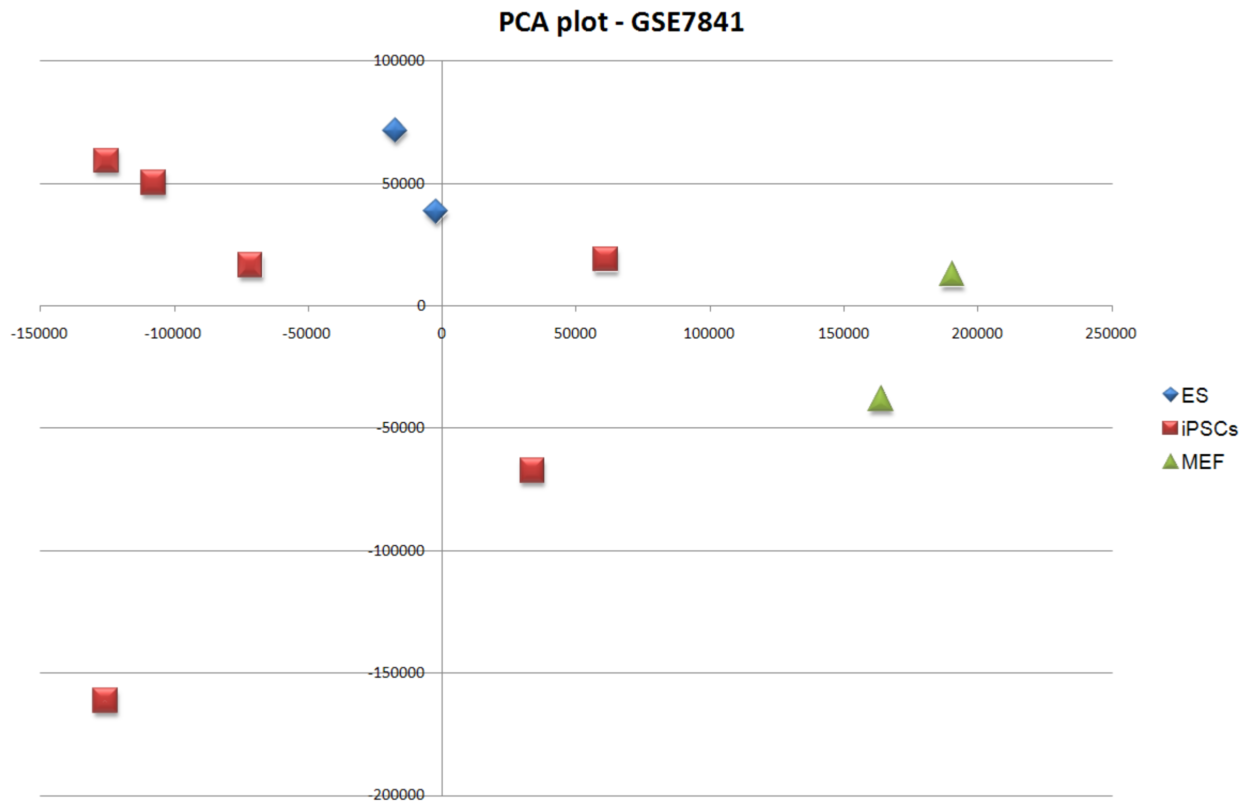
PCA plot - GSE7841
100000
50000
0
-50000
-100000
-150000
-200000
-150000
-100000
-50000
0
50000
100000
150000
200000
250000
ES
iPSCs
MEF

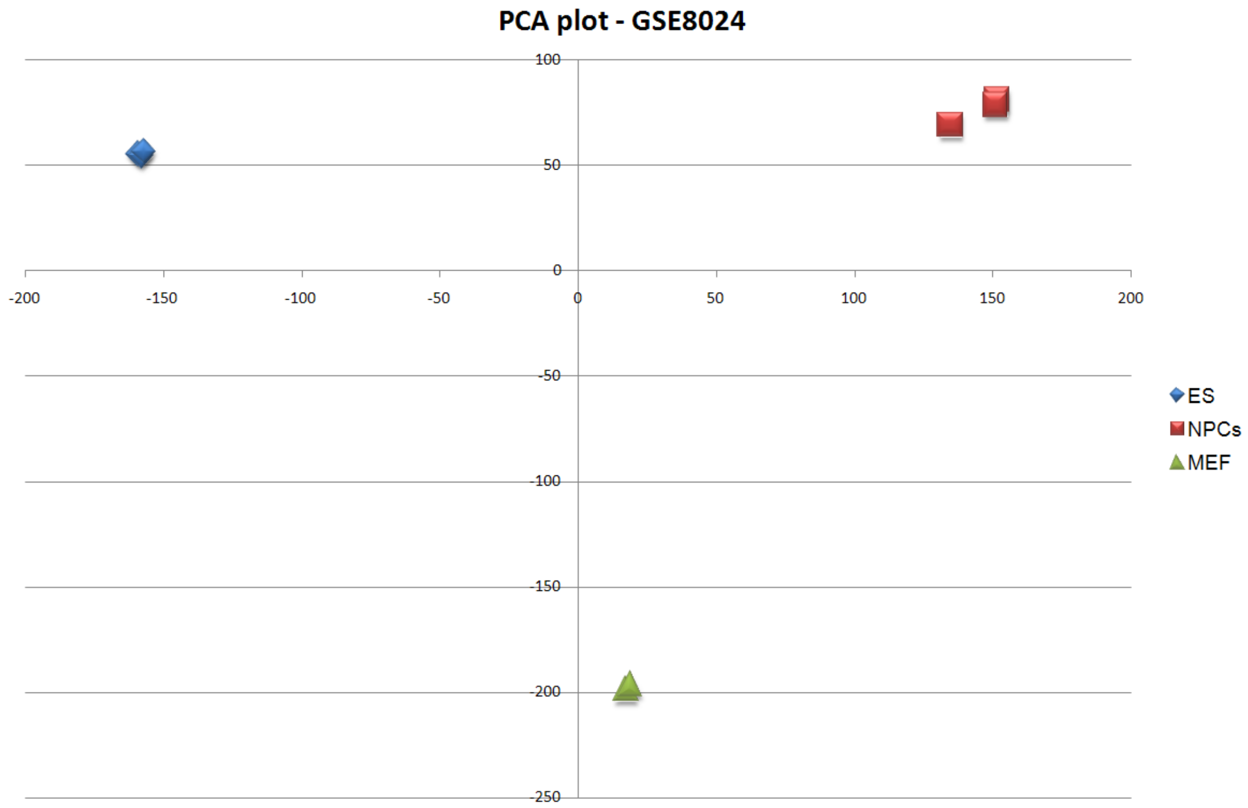
PCA plot - GSE8024
100
50
0
-50
-100
-150
-200
-250
-200
-150
-100
-50
0
50
100
150
200
ES
NPCs
MEF

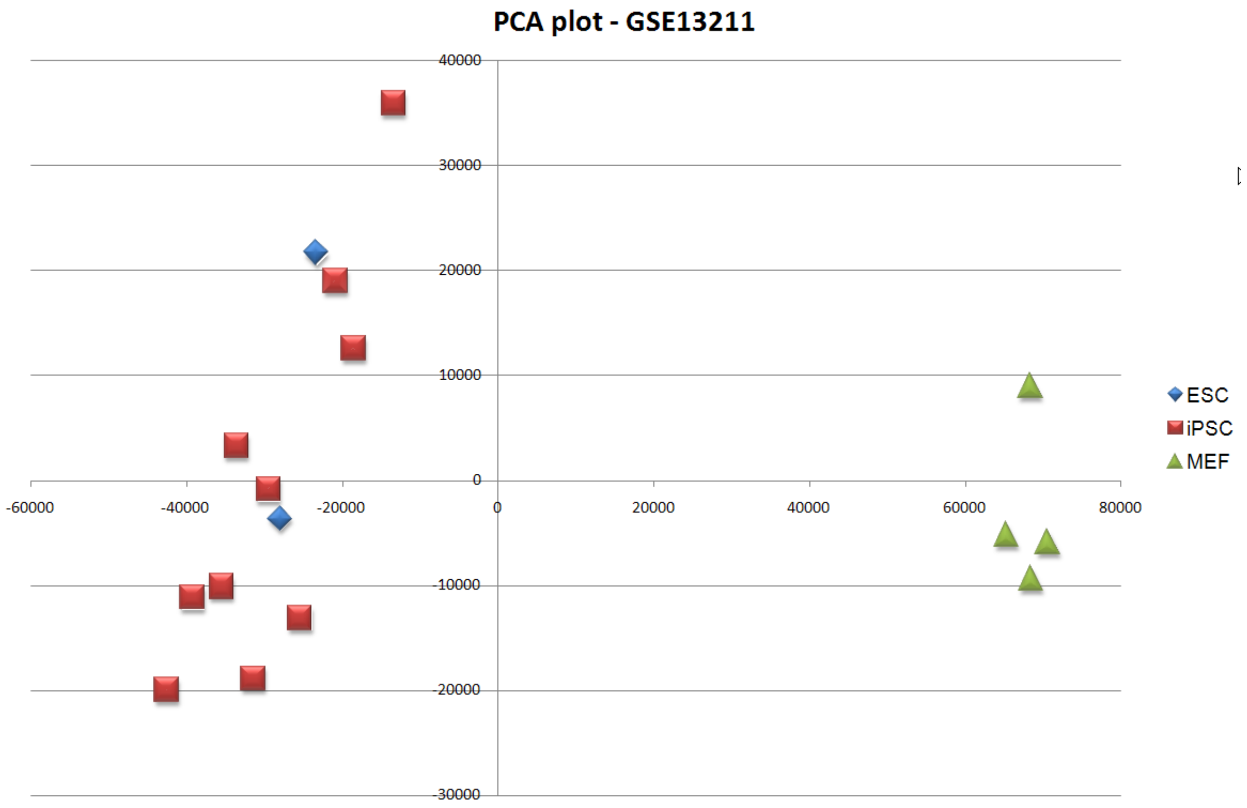

PCA plot - GSE13211
40000
30000
20000
10000
0
-10000
-20000
-30000
-60000
-40000
-20000
0
20000
40000
60000
80000
ESC
iPSC
MEF

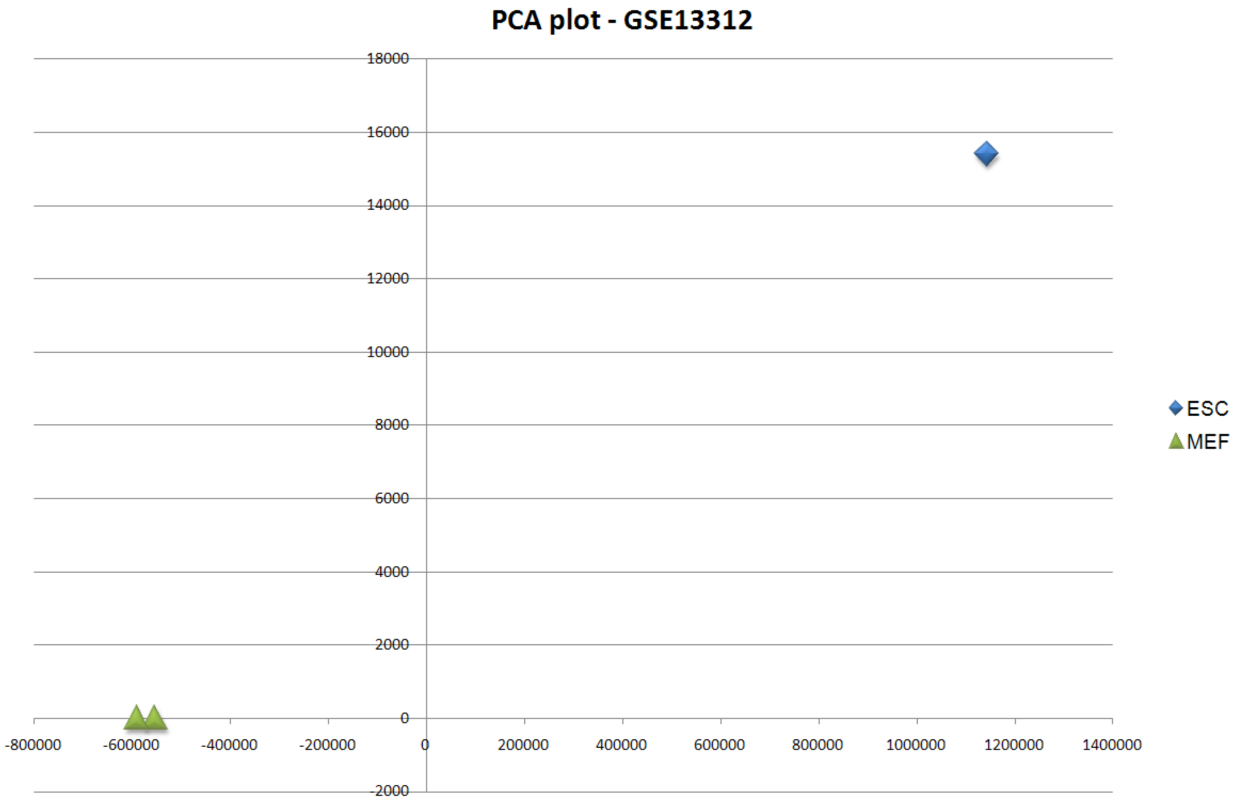

PCA plot - GSE13312
18000
16000
14000
12000
10000
8000
6000
4000
2000
0
-2000
-800000
-600000
-400000
-200000
0
200000
400000
600000
800000
1000000
1200000
1400000
ESC
MEF

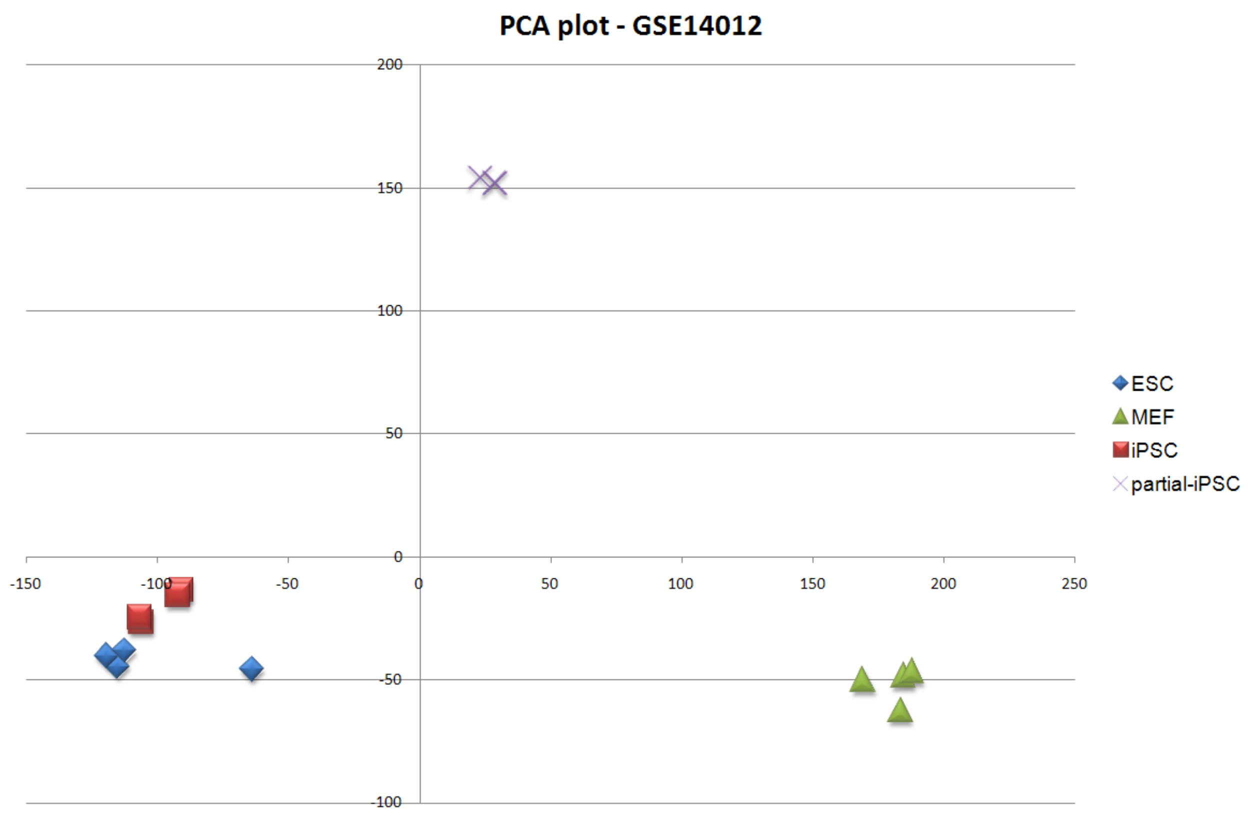

PCA plot - GSE14012
200
150
100
50
0
-50
-100
-150
-100
-50
0
50
100
150
200
250
ESC
MEF
iPSC
partial-iPSC

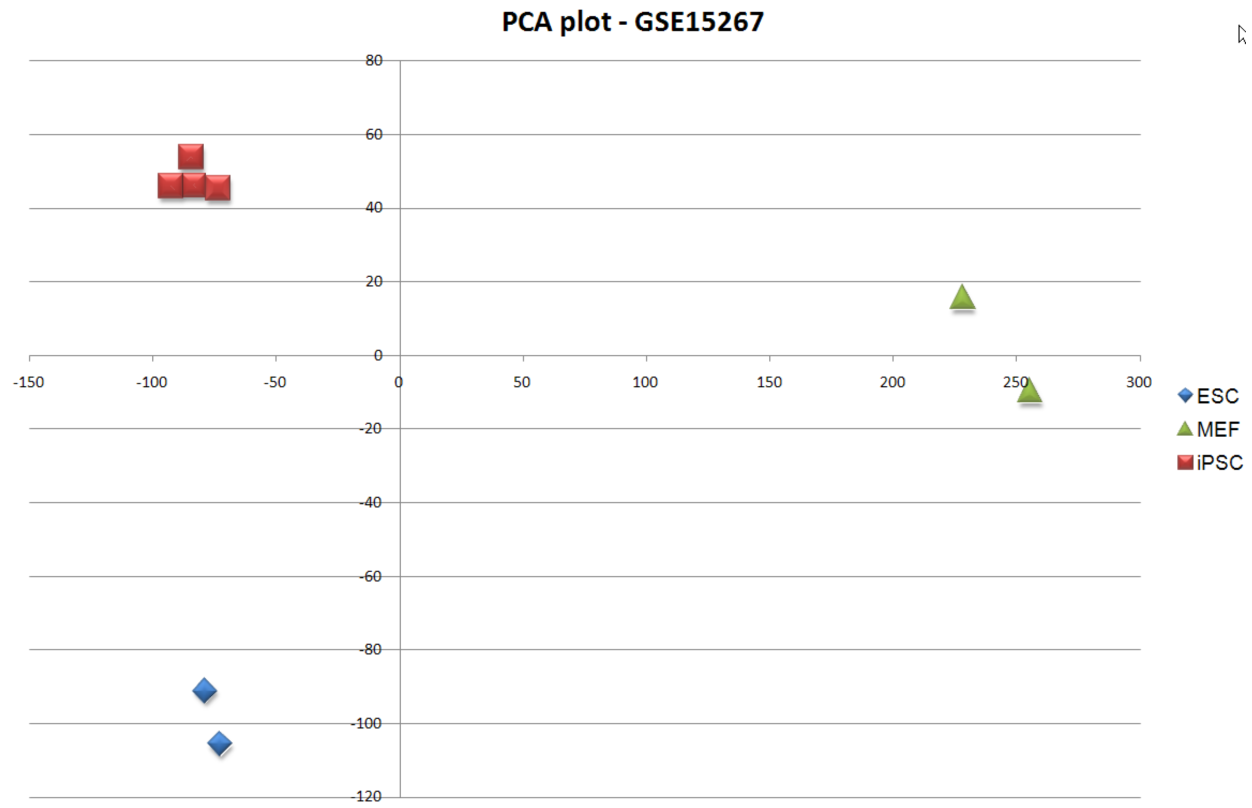

PCA plot - GSE15267
80
60
40
20
0
-20
-40
-60
-80
-100
-120
-150
-100
-50
0
50
100
150
200
250
300
ESC
MEF
iPSC

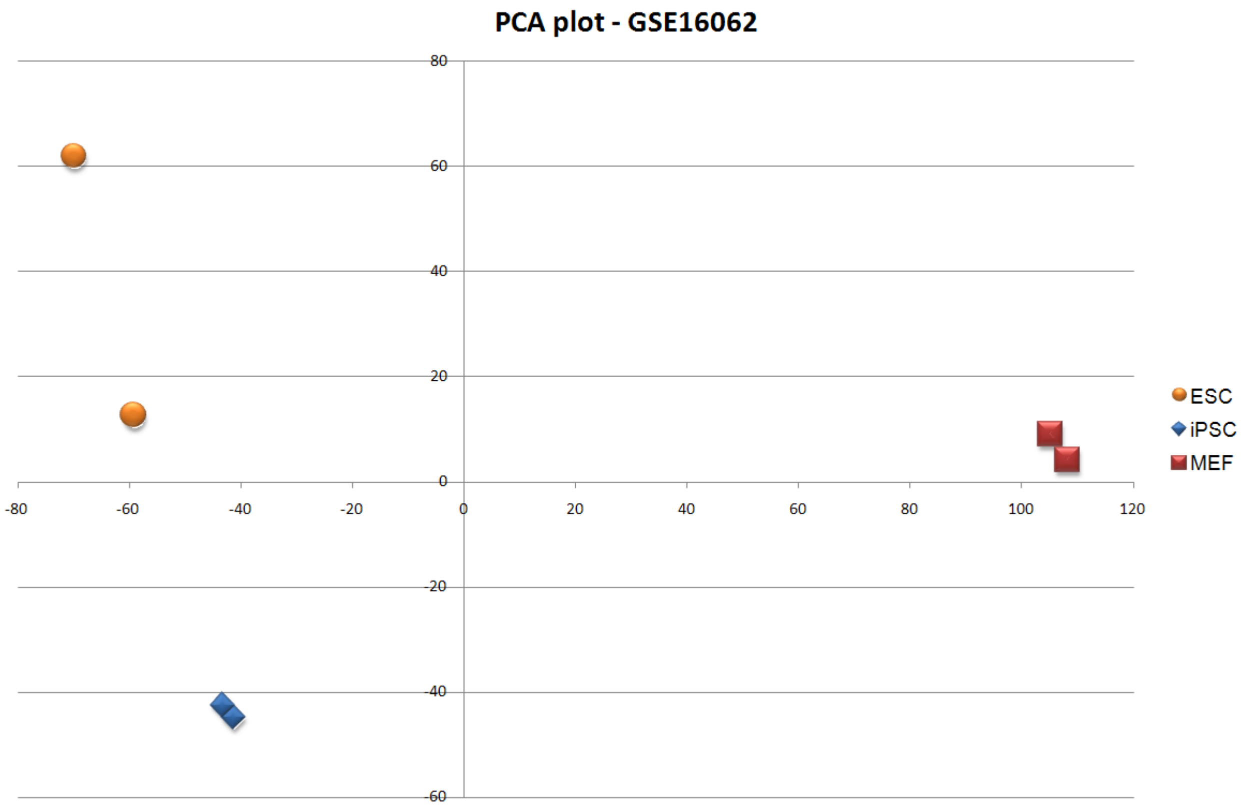

PCA plot - GSE16062
80
60
40
20
0
-20
-40
-60
-80
-60
-40
-20
0
20
40
60
80
100
120
ESC
iPSC
MEF

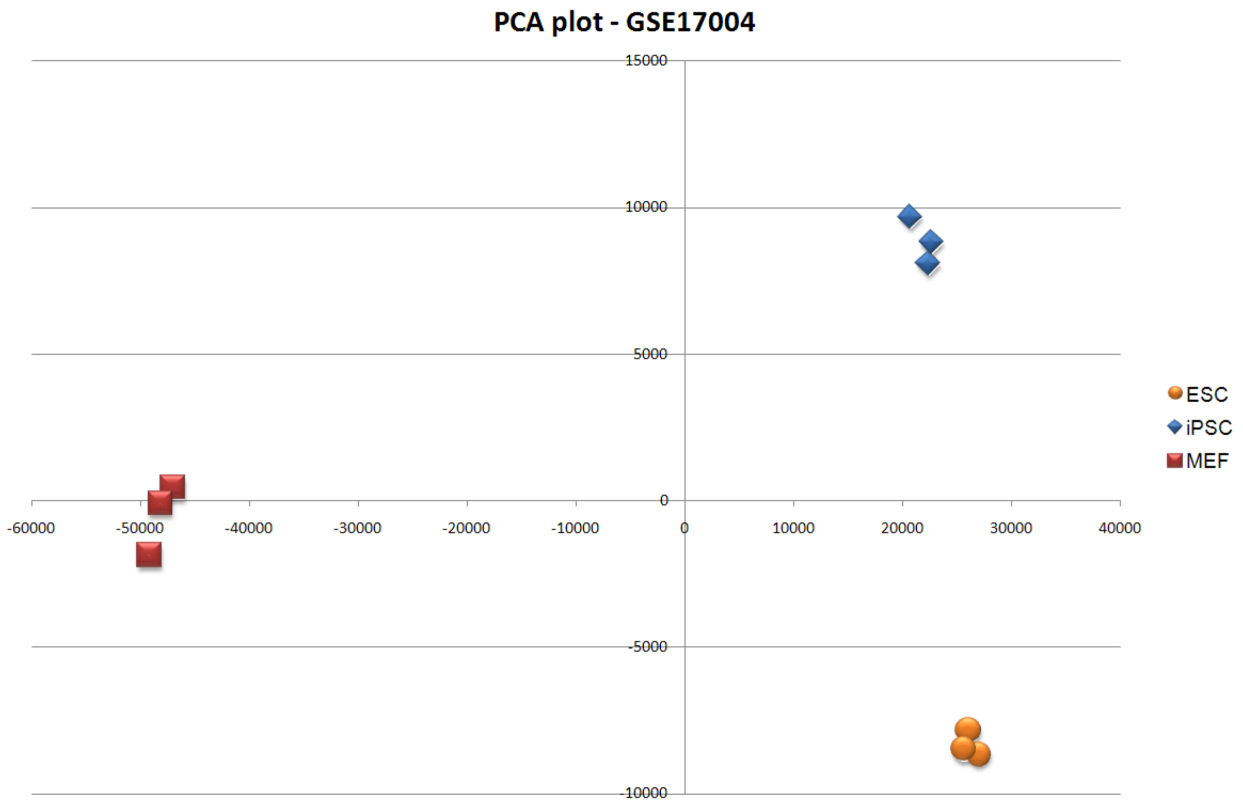

PCA plot - GSE17004
15000
10000
5000
0
-5000
-10000
-60000
-50000
-40000
-30000
-20000
-10000
0
10000
20000
30000
40000
ESC
iPSC
MEF

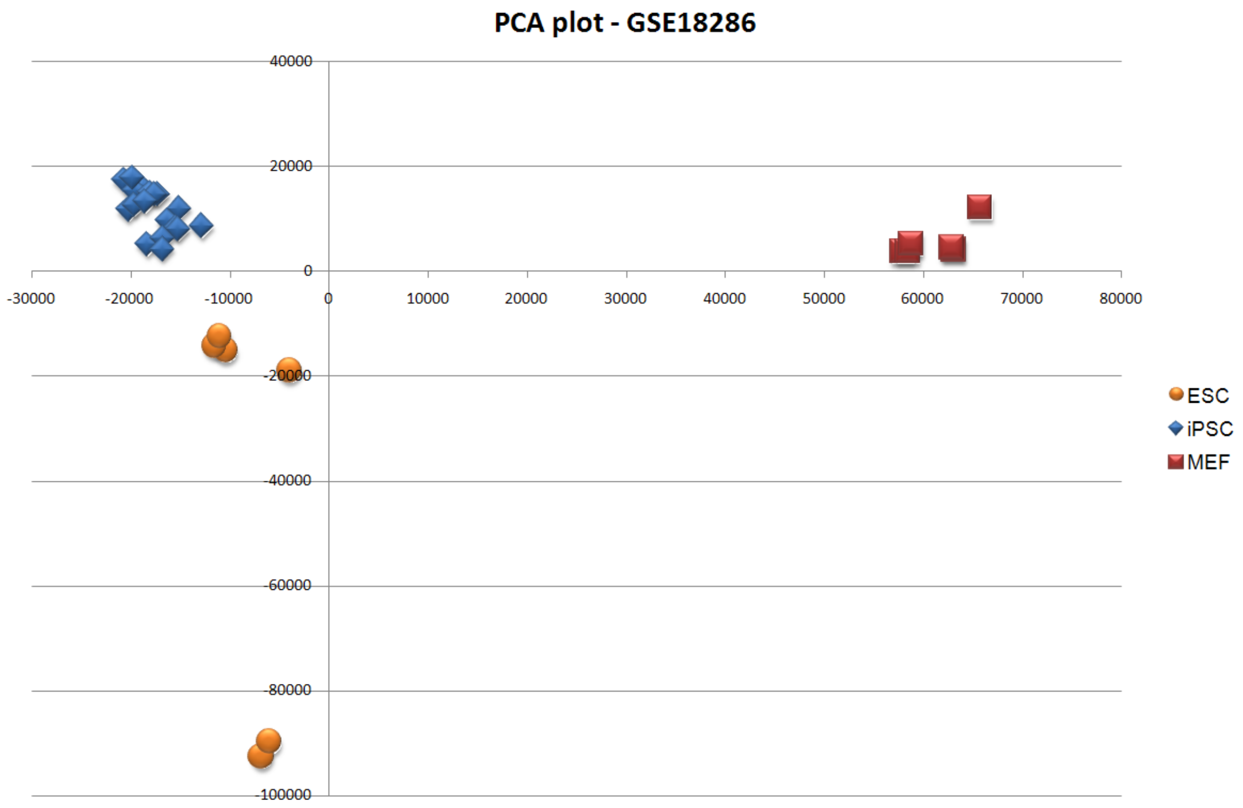

PCA plot - GSE18286
40000
20000
0
-20000
-40000
-60000
-80000
-100000
-30000
-20000
-10000
0
10000
20000
30000
40000
50000
60000
70000
80000
ESC
iPSC
MEF
