# Supplementary material for: Analysis of Human and Mouse Reprogramming of Somatic Cells to Induced Pluripotent Stem Cells. What Is in the Plate?
Source: PLoS One. 2010 Sep 17;5(9):e12664. doi: 10.1371/journal.pone.0012664 (PMC2941458; doi:10.1371/journal.pone.0012664)
Supplement: Figure S5 — Functional analysis of genes up- and downregulated in Human and Mouse ESCs and iPSCs in comparison to somatic cells using DAVID. Gene Ontology annotations of biological process, molecular function and cellular component as well as genes enrichment in KEGG pathways for the following comparisons: (A) genes upregulated in Human ESCs and iPSCs; (B) genes downregulated in Human ESCs and iPSCs; (c) genes upregulated in mouse ESCs and iPSCs; (d) genes downregulated in ESCs and iPSCs. (0.21 MB PDF) [file pone.0012664.s006.pdf]

Figure S5. Functional analysis of genes up- and downregulated in Human and Mouse ESCs and iPSCs in comparison to somatic cells using DAVID

Gene Ontology annotations of biological process, molecular function and cellular component as well as genes enrichment in KEGG pathways for the following comparisons: (A) genes upregulated in Human ESCs and iPSCs; (B) genes downregulated in Human ESCs and iPSCs; (c) genes upregulated in mouse ESCs and iPSCs; (d) genes downregulated in ESCs and iPSCs

A. Functional analysis of genes upregulated in Human ESCs and iPSCs in comparison to somatic cells using DAVID

(a) Gene Ontology annotations of biological process, molecular function and cellular component (b) Genes enrichment in KEGG pathways

(a) **Gene Ontology annotations of biological process, molecular function and cellular component.** Significantly ($p<0.01$) overrepresented annotations are shown as blue bars.

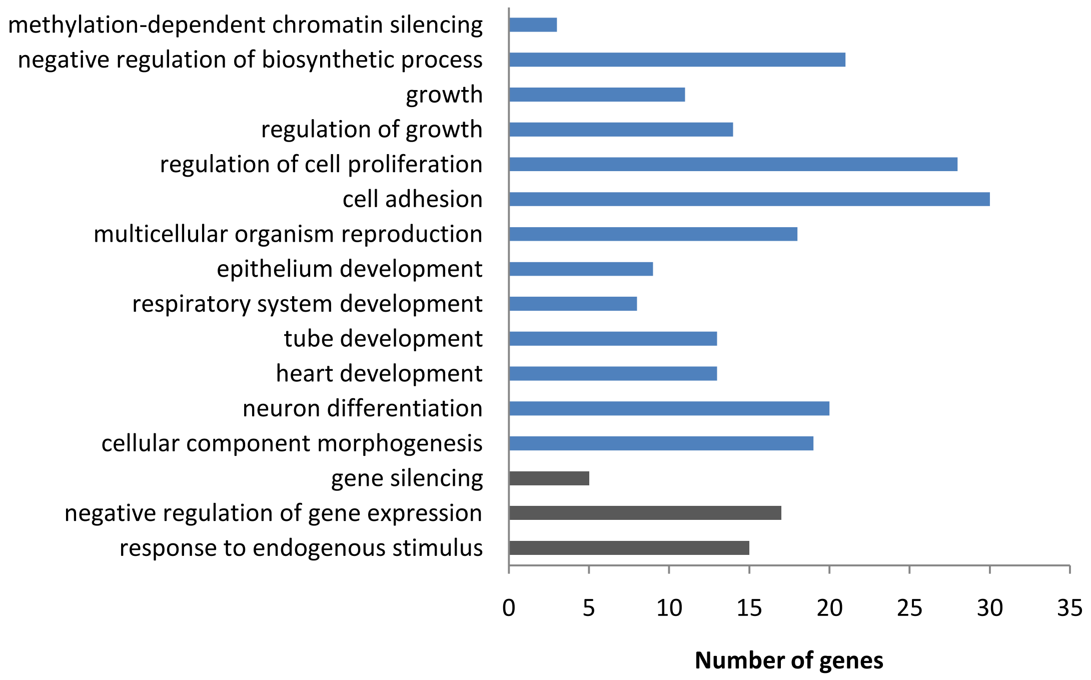

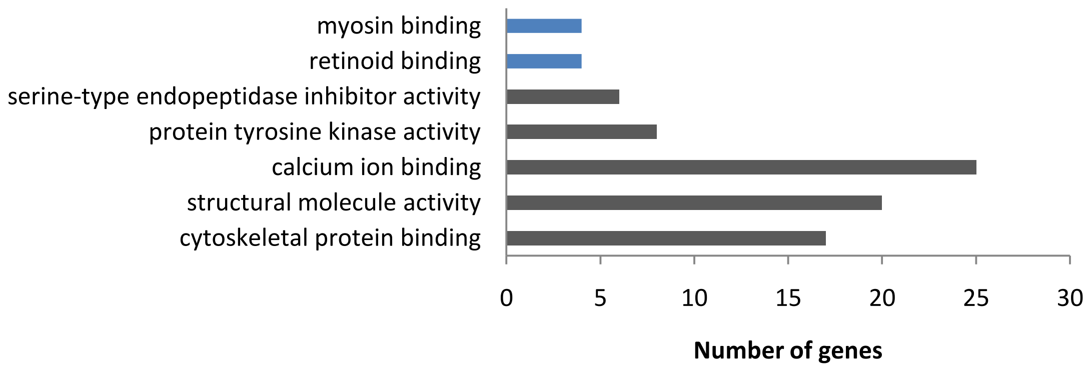

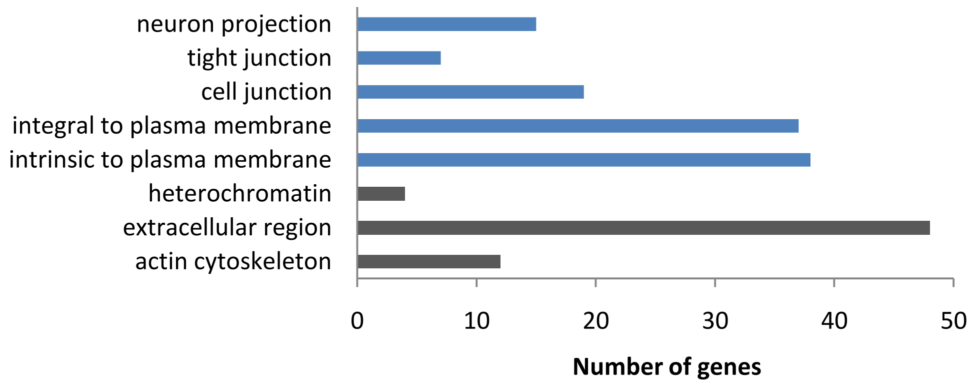

(b) Genes enrichment in KEGG pathways. Significantly ($p<0.01$) enriched pathways are shown in bold.

KEGG pathway	Number of genes
**Cell adhesion molecules (CAMs)**	8
Tight junction	7
TGF-beta signaling pathway	5
Wnt signaling pathway	6
Pathways in cancer	8
Regulation of actin cytoskeleton	5
Leukocyte transendothelial migration	5

B. Functional analysis of genes downregulated in Human ESCs and iPSCs in comparison to somatic cells using DAVID

(a) Gene Ontology annotations of biological process, molecular function and cellular component (b) Genes enrichment in KEGG pathways

(a) Gene Ontology annotations of biological process, molecular function and cellular component. Significantly ($p<0.01$) overrepresented annotations are shown as blue bars.

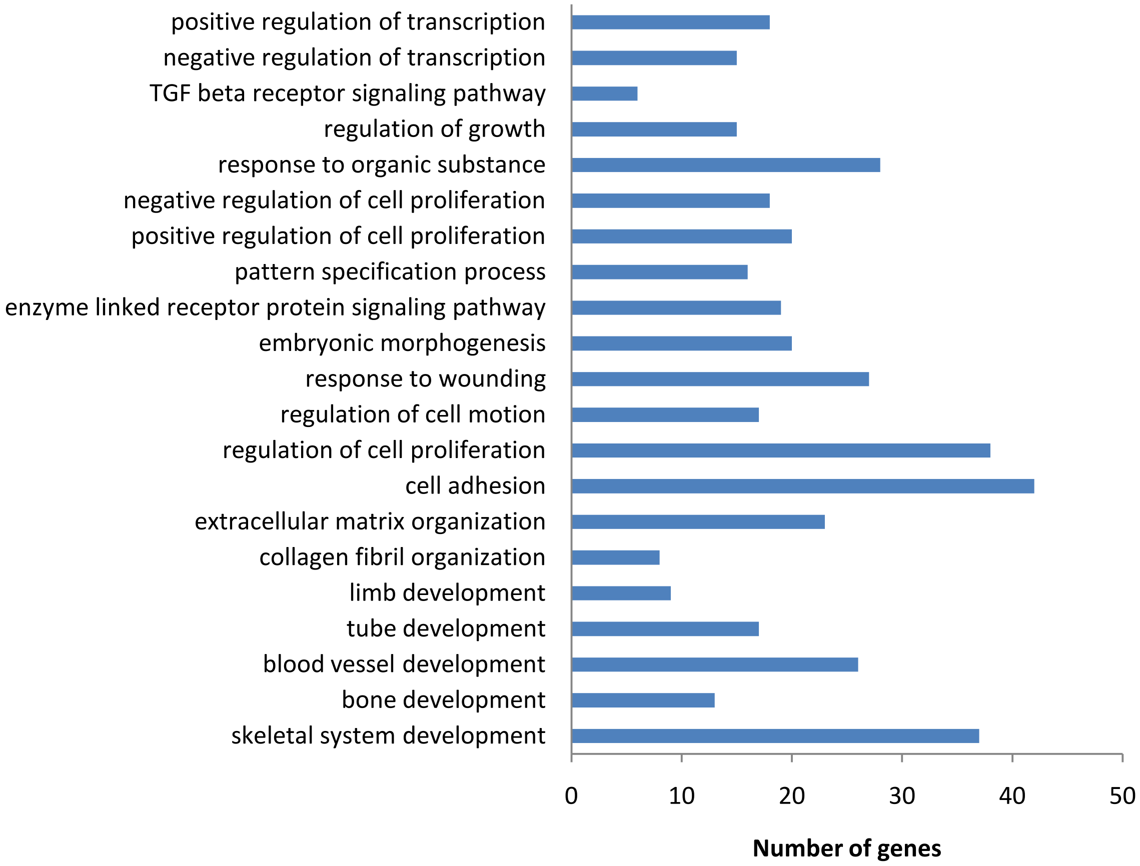

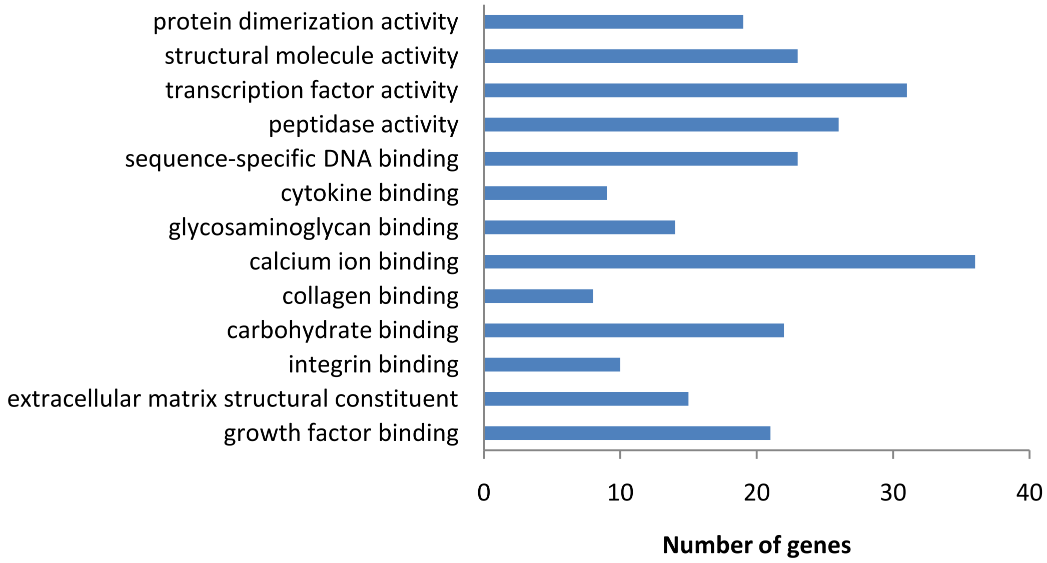

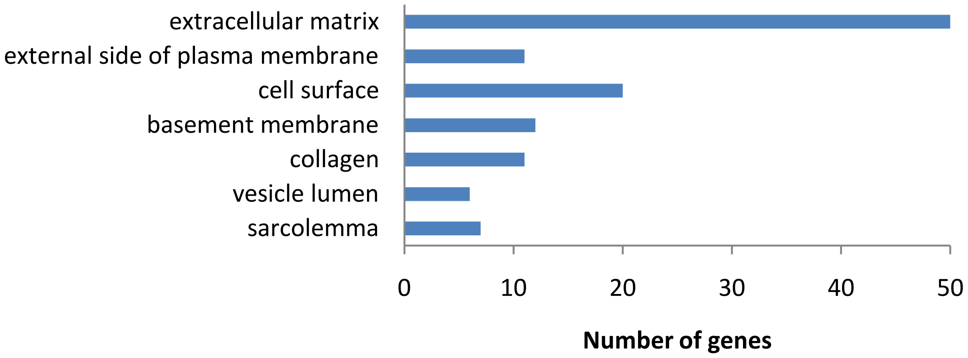

(b) Genes enrichment in KEGG pathways. Significantly ($p<0.01$) enriched pathways are shown in bold.

KEGG pathway	Number of genes
**ECM-receptor interaction**	14
**Focal adhesion**	20
**Pathways in cancer**	16
**Complement and coagulation cascades**	7
**Cytokine-cytokine receptor interaction**	13
**Hematopoietic cell lineage**	7
**Bladder cancer**	5
Axon guidance	5
MAPK signaling pathway	7
Calcium signaling pathway	7
Regulation of actin cytoskeleton	8
Gap junction	5
Prostate cancer	5
TGF-beta signaling pathway	5
Melanoma	5

C. Functional analysis of genes upregulated in mouse ESCs and iPSCs in comparison to MEF using DAVID

(a) Gene Ontology annotations of biological process, molecular function and cellular component (b) Genes enrichment in KEGG pathways

(a) Gene Ontology annotations of biological process, molecular function and cellular component. Significantly ($p<0.01$) overrepresented annotations are shown as blue bars.

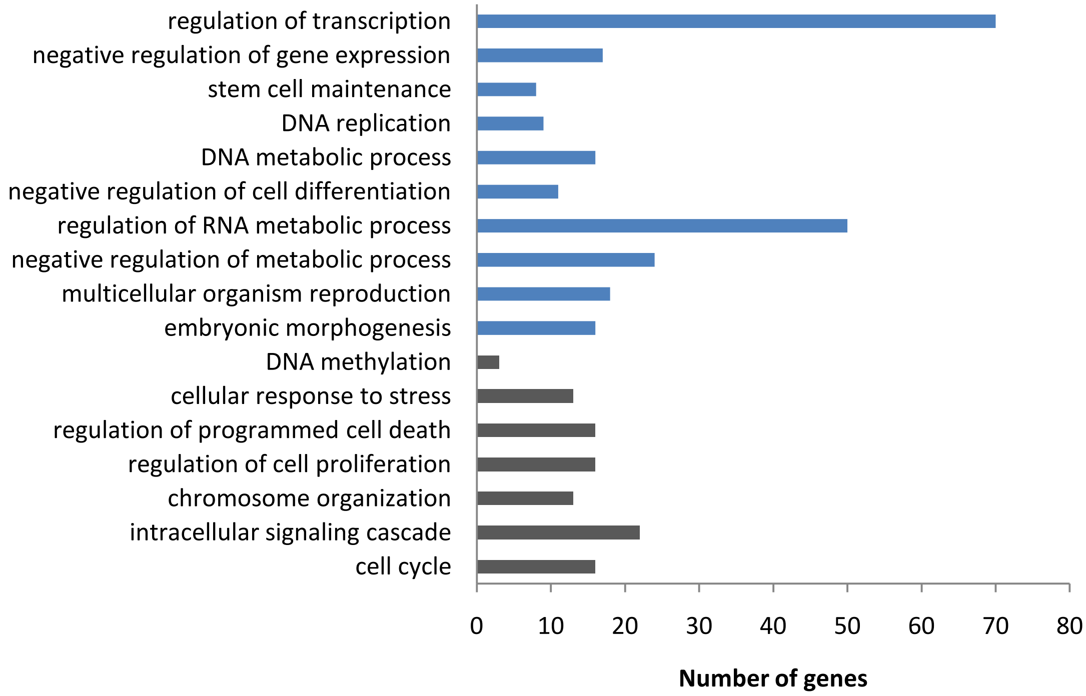

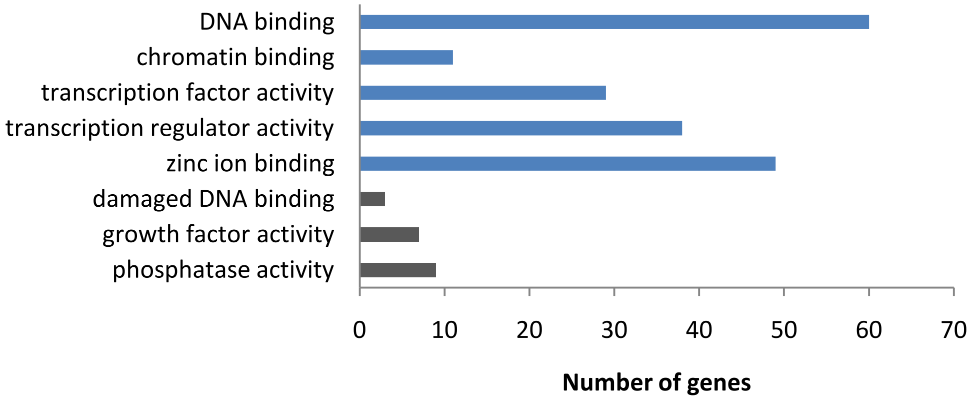

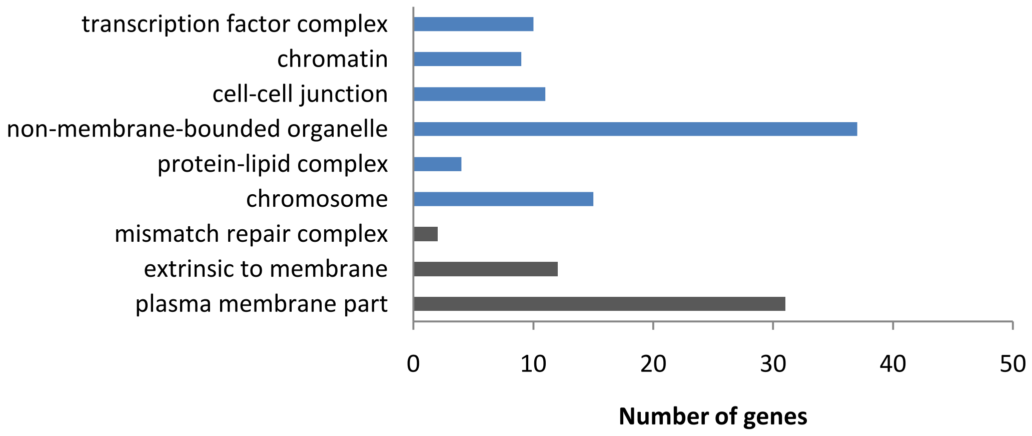

(b) Genes enrichment in KEGG pathways. Significantly ($p<0.01$) enriched pathways are shown in bold.

KEGG pathway	Number of genes
**Tight junction**	9
**Cell adhesion molecules (CAMs)**	8
Pathways in cancer	8
Leukocyte transendothelial migration	6
MAPK signaling pathway	6
Endocytosis	5
Glycine, serine and threonine metabolism	4
Long-term potentiation	4
Arachidonic acid metabolism	4
Colorectal cancer	4
Neurotrophin signaling pathway	4
Purine metabolism	4

D. Functional analysis of genes downregulated in mouse ESCs and iPSCs in comparison to MEF using DAVID

(a) Gene Ontology annotations of biological process, molecular function and cellular component (b) Genes enrichment in KEGG pathways

(a) Gene Ontology annotations of biological process, molecular function and cellular component. Significantly ($p<0.01$) overrepresented annotations are shown as blue bars.

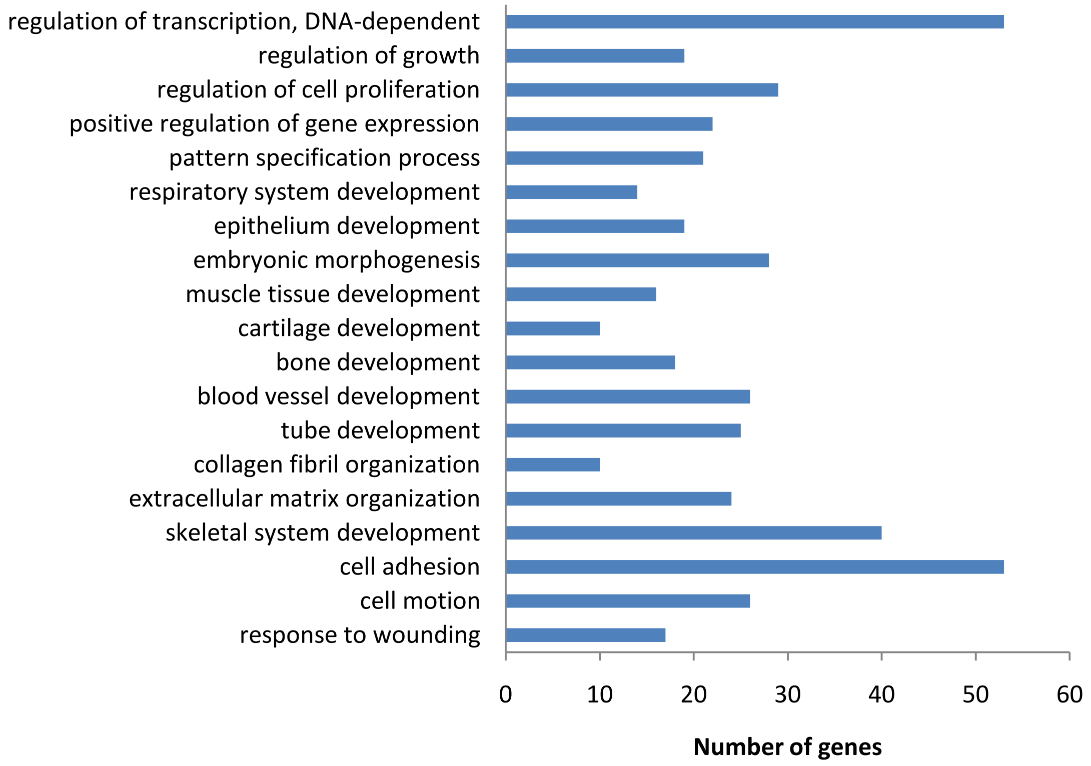

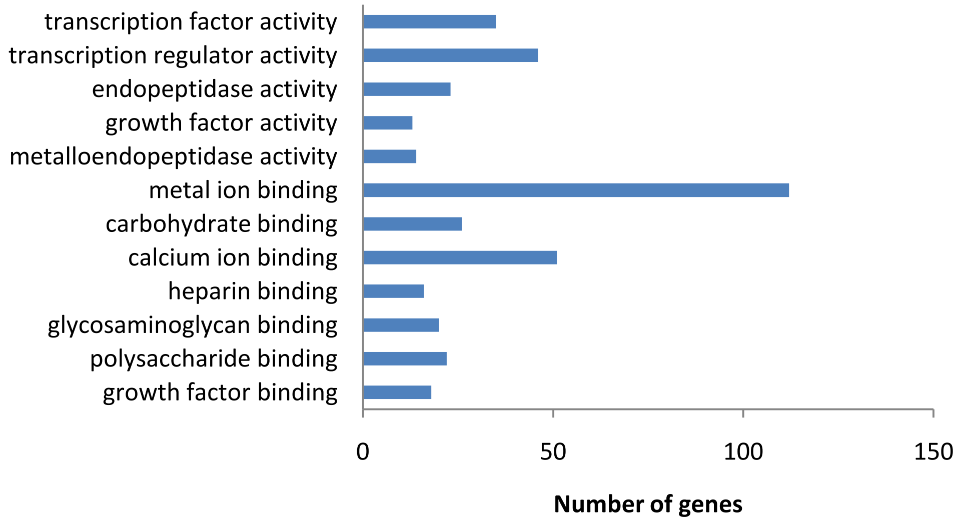

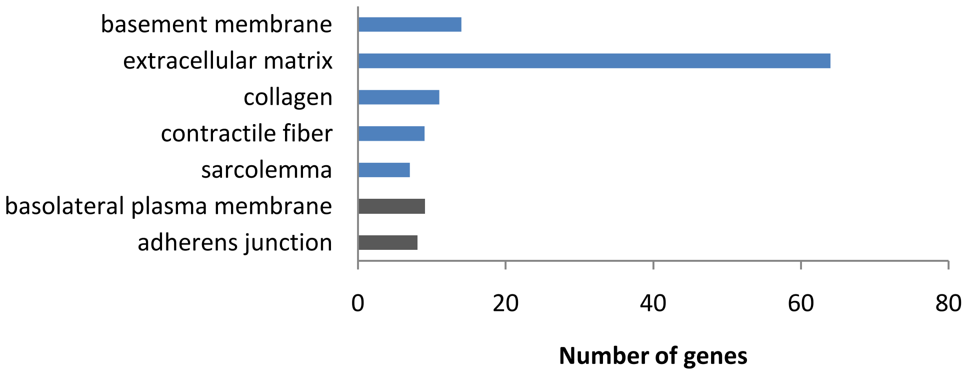

(b) Genes enrichment in KEGG pathways. Significantly (p<0.01) enriched pathways are shown in bold.

KEGG pathway	Number of genes
**Focal adhesion**	29
**ECM-receptor interaction**	20
**Pathways in cancer**	20
**Cytokine-cytokine receptor interaction**	17
**Colorectal cancer**	9
**TGF-beta signaling pathway**	9
**Vascular smooth muscle contraction**	9
**Hypertrophic cardiomyopathy (HCM)**	7
MAPK signaling pathway	11
Regulation of actin cytoskeleton	9
Dilated cardiomyopathy	7
Axon guidance	7
Cell adhesion molecules (CAMs)	7
Calcium signaling pathway	7
Melanoma	6
Prostate cancer	6
Chemokine signaling pathway	6
Chronic myeloid leukemia	6
Leukocyte transendothelial migration	6
