# Supplementary material for: Analysis of Human and Mouse Reprogramming of Somatic Cells to Induced Pluripotent Stem Cells. What Is in the Plate?
Source: PLoS One. 2010 Sep 17;5(9):e12664. doi: 10.1371/journal.pone.0012664 (PMC2941458; doi:10.1371/journal.pone.0012664)

Figure S6. Mouse network of genes upregulated in both ESCs and iPSCs compared to MEF - Overlap with transcription factors binding and chromatin marks in ESCs

Interaction network of genes upregulated in mouse ESCs and iPSCs compared to MEFs coloring genes which (A) are transcription factors, are targets in ESCs of (B) Nanog, (C) Sox2, (D) Oct4, (E) Klf4, (F) c-Myc, (G) Dax1, (H) Nac1, (I) Zfp281, (J) Rex1, (K) Eed, (L) Suz12.

Interaction network of genes upregulated in mouse ESCs and iPSCs compared to MEFs highlighting the chromatin status (marks H3K4me3, usually correlated with expression, and H3K27me3, usually correlated with repression) of the genes in (M) MEFs, (N) ESCs.

Overlap with the published Oct4 network in mouse ESCs.

A. Highlight of transcription factors in the mouse network of most significantly upregulated genes in ES and iPS cells compared to MEF.

Transcription factors's nodes are colored in red. The central part of the network where are most known "pluripotency factors" shows a very high overrepresentation of transcription factors.

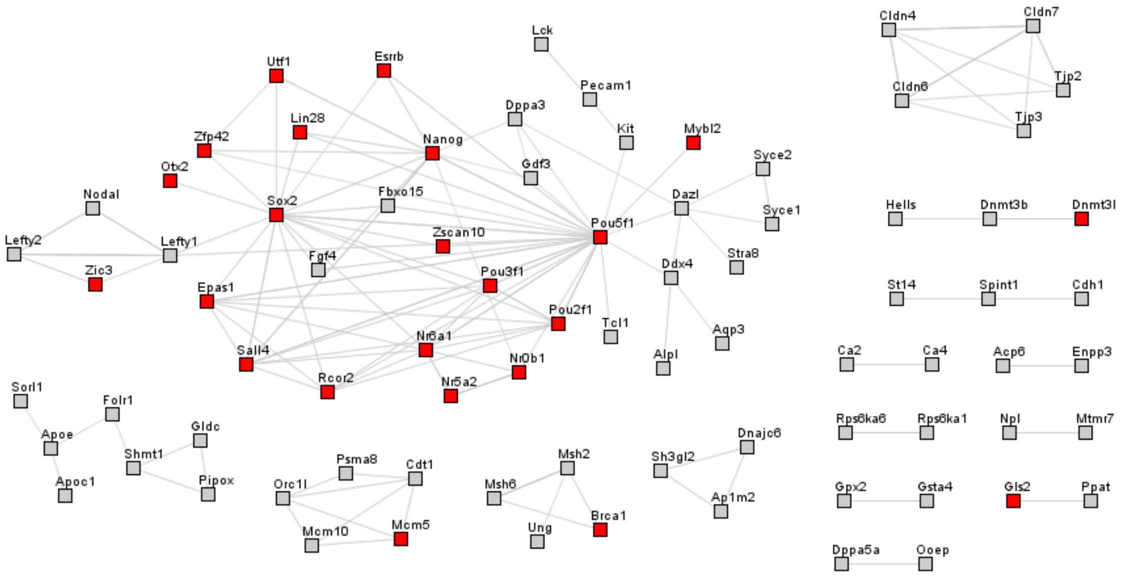

B. Highlight of genes whose promoter is bound by Nanog in the mouse network of most signi1ficantly upregulated genes in ES and iPS cells compared to MEF.

Genes bound by Nanog are colored in green. Again, most genes of the central "pluripotency" network are bound by Nanog

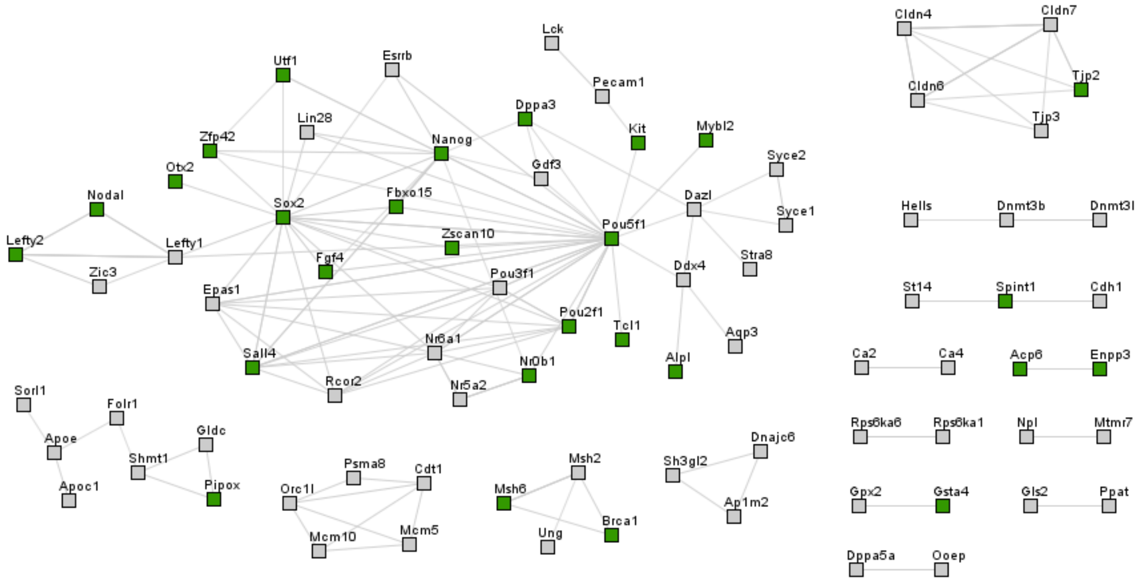

C. Highlight of genes whose promoter is bound by Sox2 in the mouse network of most significantly upregulated genes in ES and iPS cells compared to MEF.

Genes bound by Sox2 are colored in pink. Again, most genes of the central "pluripotency" network are bound by Sox2.

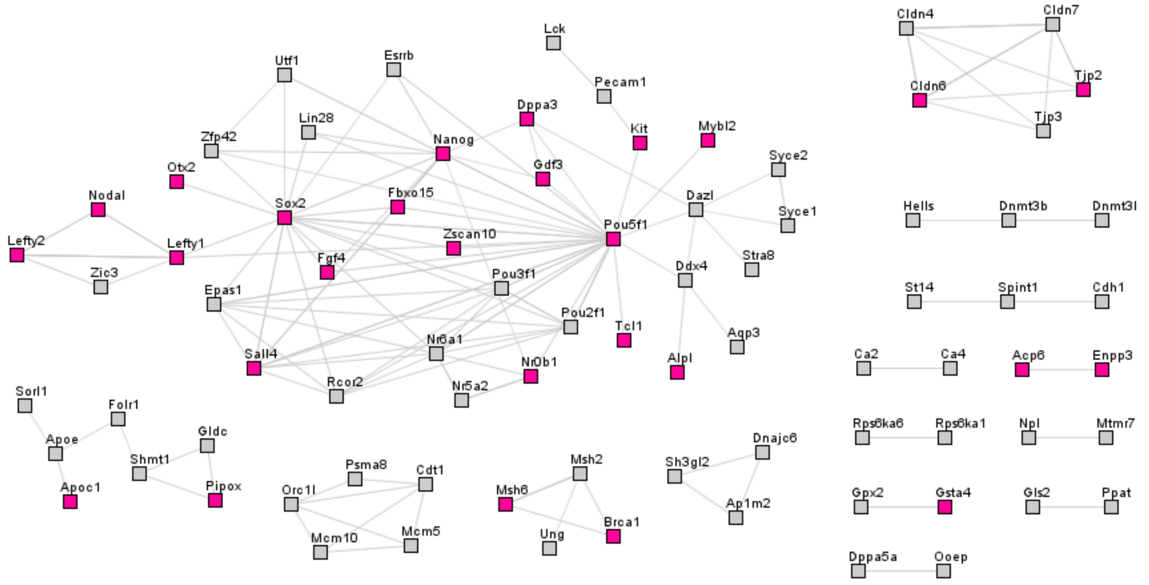

D. Highlight of genes whose promoter is bound by Pou5f1 (Oct4) in the mouse network of most significantly upregulated genes in ES and iPS cells compared to MEF.

Genes bound by Pou5f1 (Oct4) are colored in blue. Many genes of the central "pluripotency" network are bound by Pou5f1, but also a significant number of genes related to other functions.

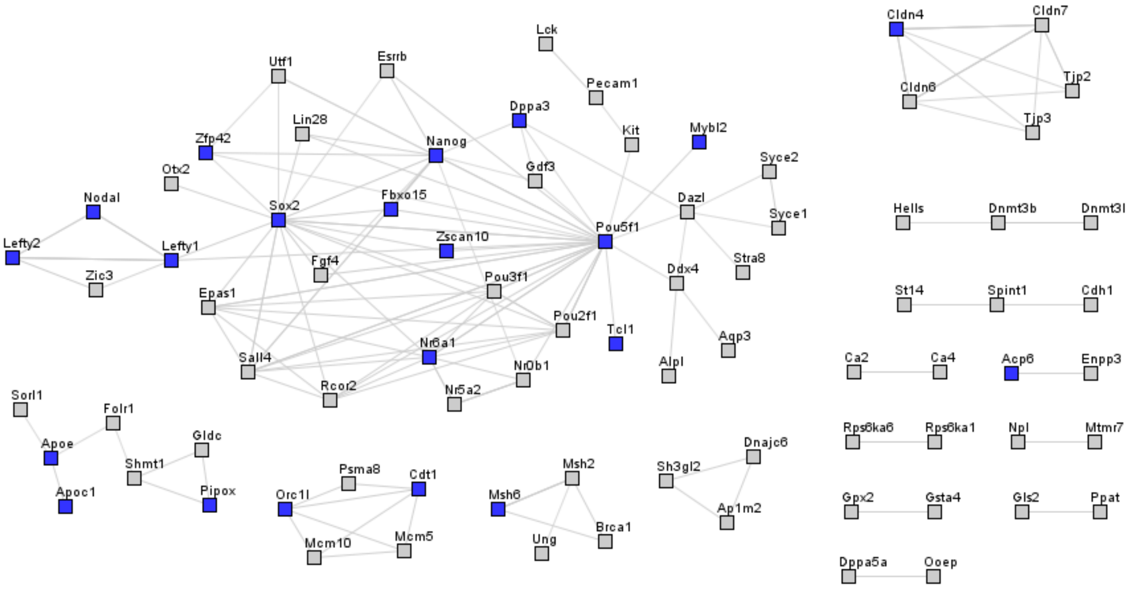

E. Highlight of genes whose promoter is bound by Klf4 in the mouse network of most significantly upregulated genes in ES and iPS cells compared to MEF.

Genes bound by Klf4 are colored in green. Again, most genes of the central "pluripotency" network are bound by Klf4, but also many genes linked to other biological processed, such as tight junctions, DNA repair, or other metabolism.

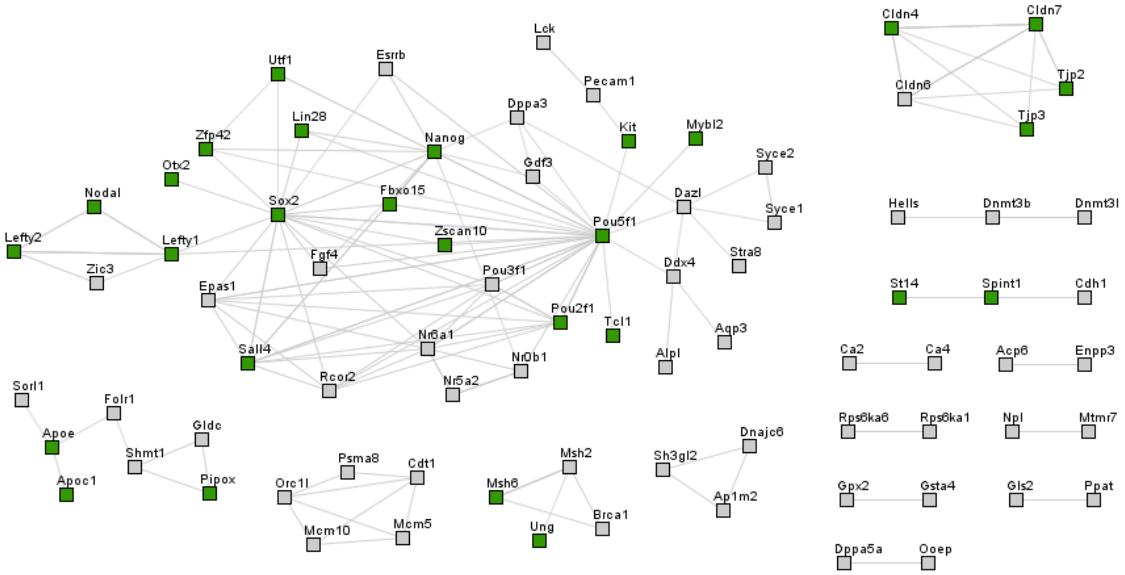

F. Highlight of genes whose promoter is bound by c-Myc in the mouse network of most significantly upregulated genes in ES and iPS cells compared to MEF.

Genes bound by c-Myc are colored in yellow. Many genes of the central “pluripotency” network are bound by c-Myc, but it seems like it binds mostly genes related to other functions, such as DNA or other metabolism.

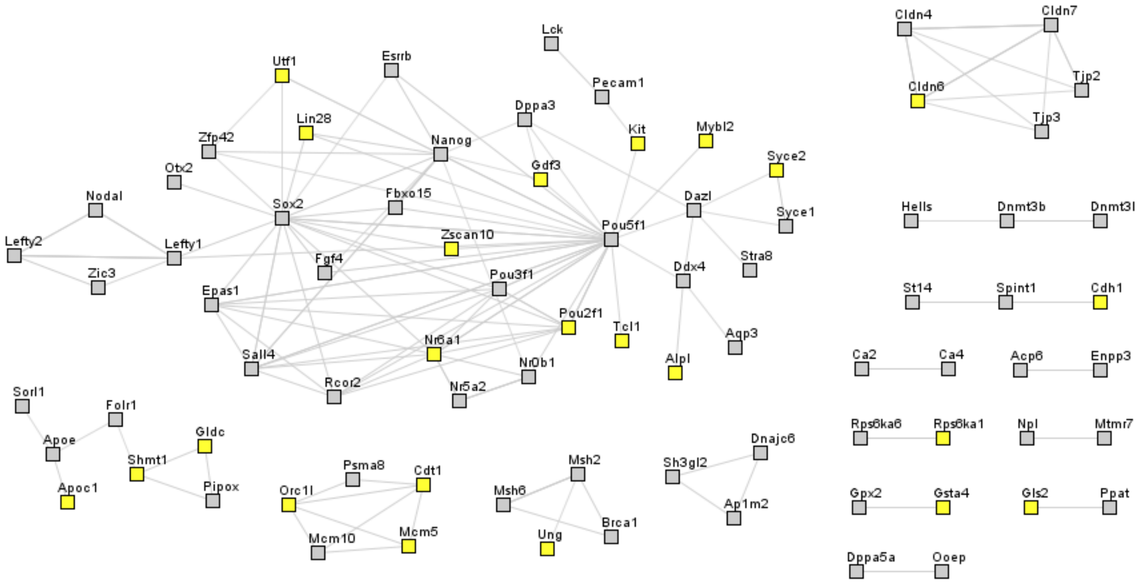

G. Highlight of genes whose promoter is bound by Dax1 in the mouse network of most significantly upregulated genes in ES and iPS cells compared to MEF.

Genes bound by Dax1 are colored in blue. Again, most genes of the central “pluripotency” network are bound by Dax1, but also a significant number of genes involved in other functions.

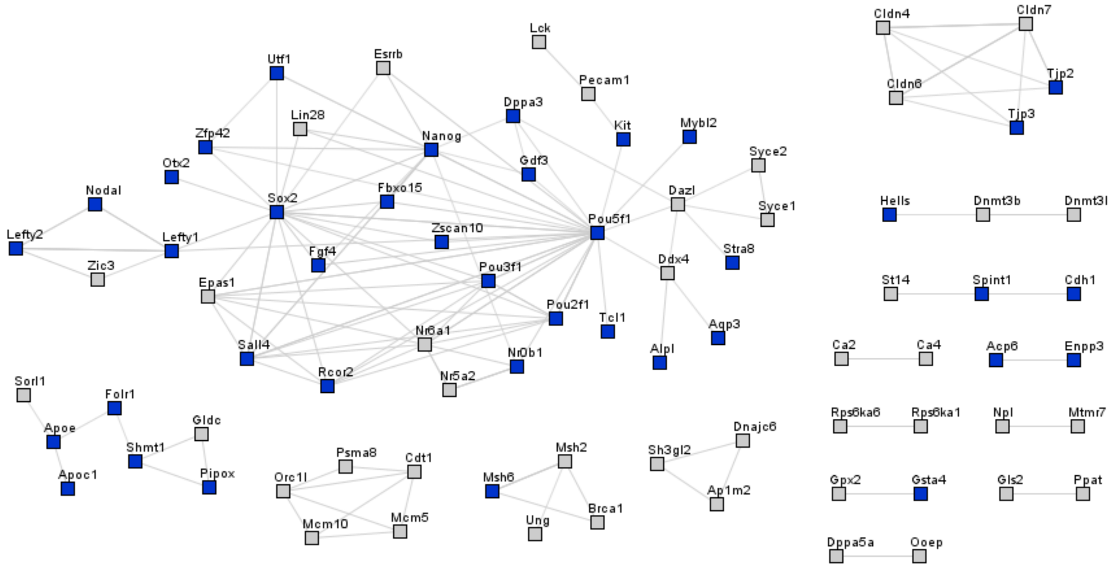

H. Highlight of genes whose promoter is bound by Nac1 in the mouse network of most significantly upregulated genes in ES and iPS cells compared to MEF.

Genes bound by Nac1 are colored in pink. Again, most genes of the central "pluripotency" network are bound by Nac1.

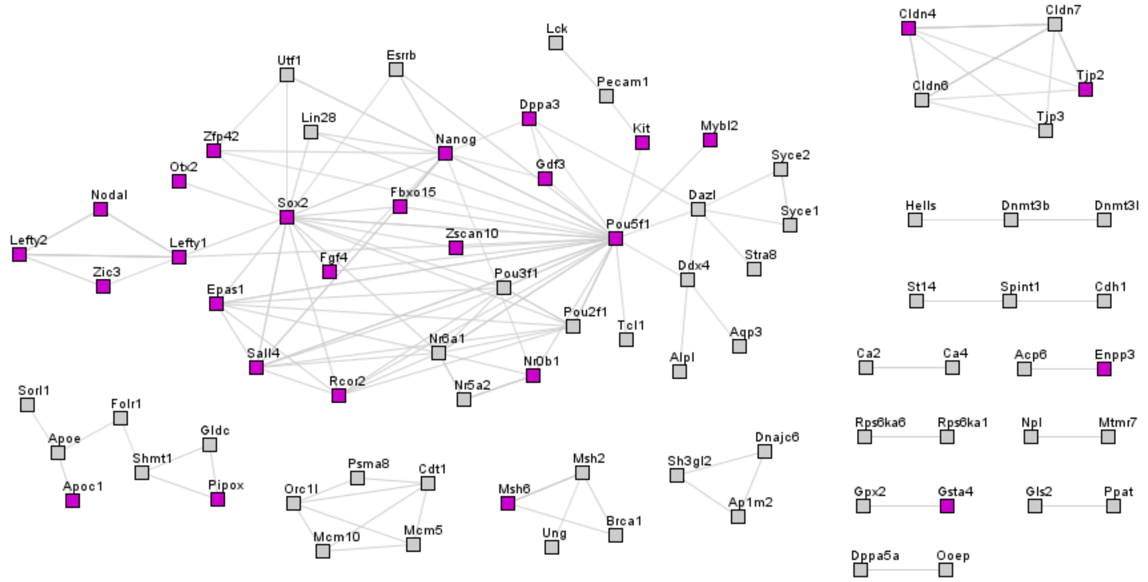

I. Highlight of genes whose promoter is bound by Zfp281 in the mouse network of most significantly upregulated genes in ES and iPS cells compared to MEF.

Genes bound by Zfp281 are colored in dark blue. This gene binds less genes in this network, but most of them are part of the core pluripotency network.

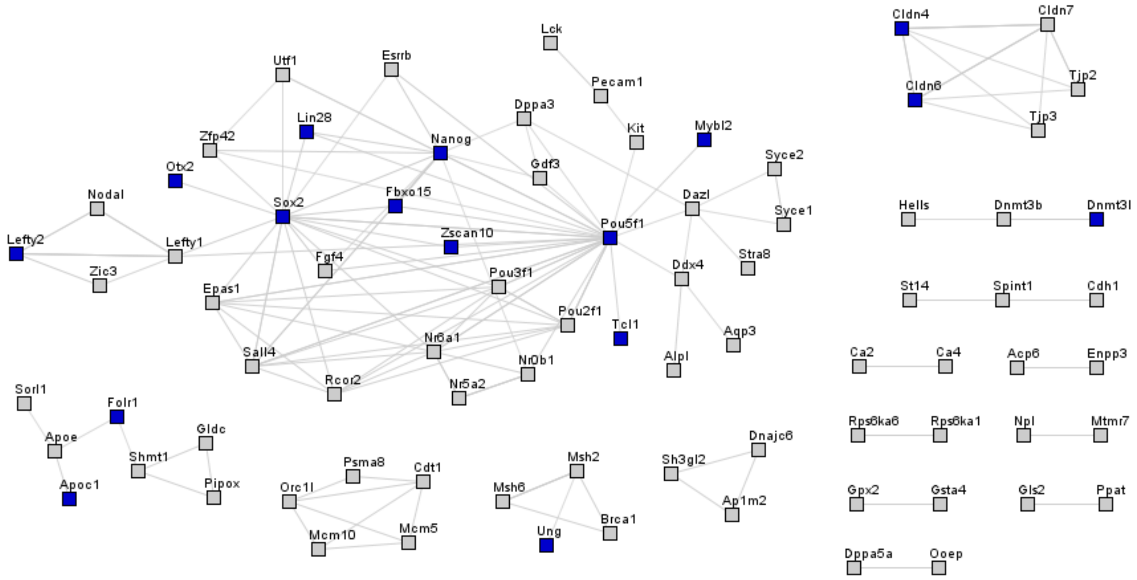

J. Highlight of genes whose promoter is bound by Rex1 in the mouse network of most significantly upregulated genes in ES and iPS cells compared to MEF

Genes bound by Rex1 are colored in yellow. Rex1 binds some genes of the core regulatory network, but also some “auxiliary” ones.

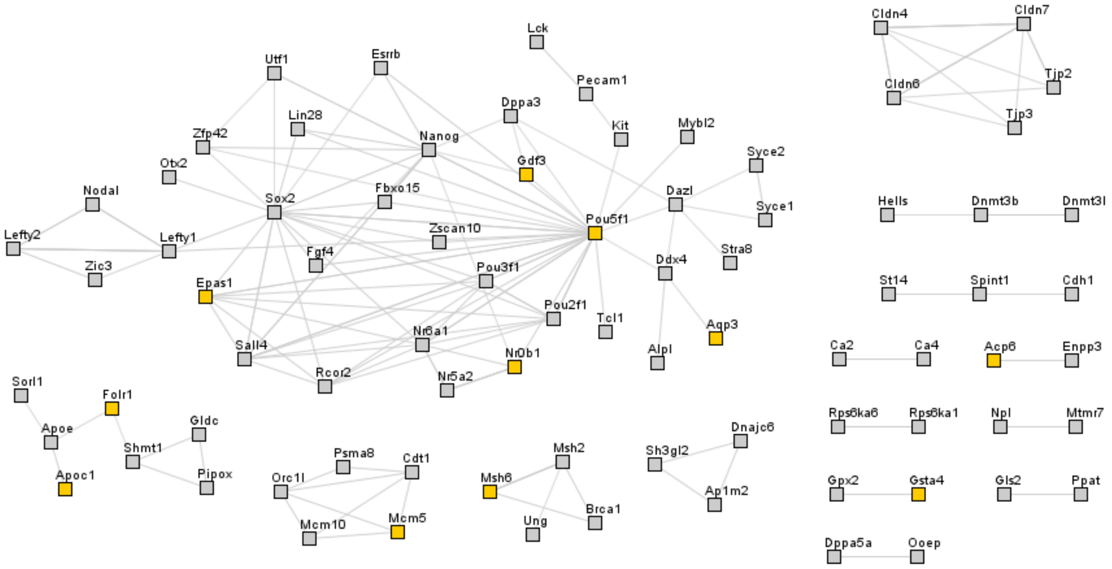

K. Highlight of genes whose promoter is bound by Eed in the mouse network of most significantly upregulated genes in ES and iPS cells compared to MEF.

Genes bound by Eed are colored in red. As expected, few genes are bound by this polycomb-complex member. Moreover, and as can be seen in Figure S4, those are usually not the most reliable genes of the network.

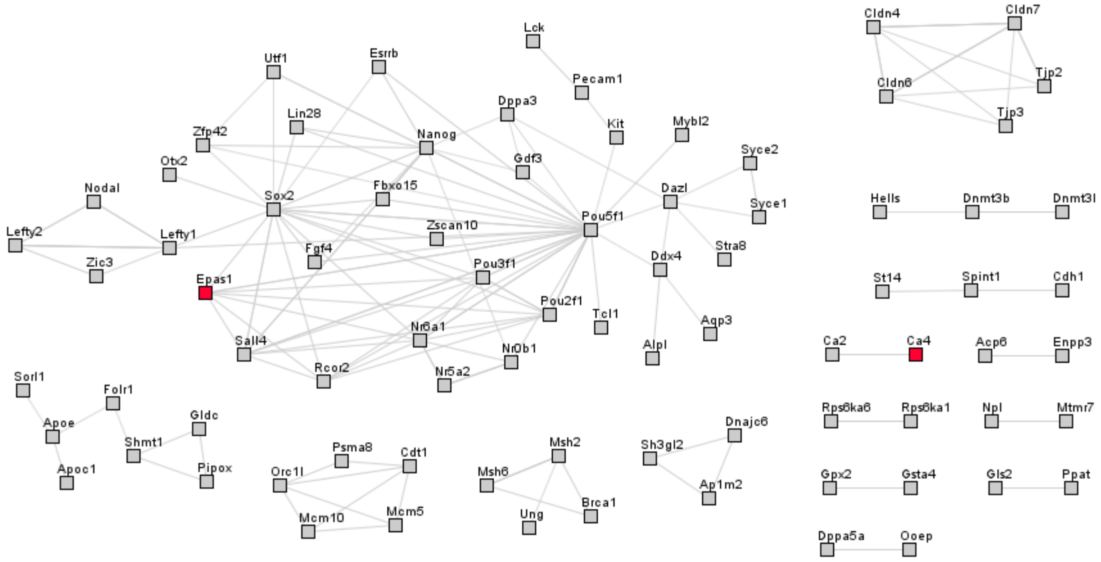

L. Highlight of genes whose promoter is bound by Suz12 in the mouse network of most significantly upregulated genes in ES and iPS cells compared to MEF.

Genes bound by Suz12 are colored in red. As in Figure 21 and as expected, few genes are bound by this polycomb-complex member. Moreover, and as can be seen in Figure S4 those are not the most consistent genes of the network.

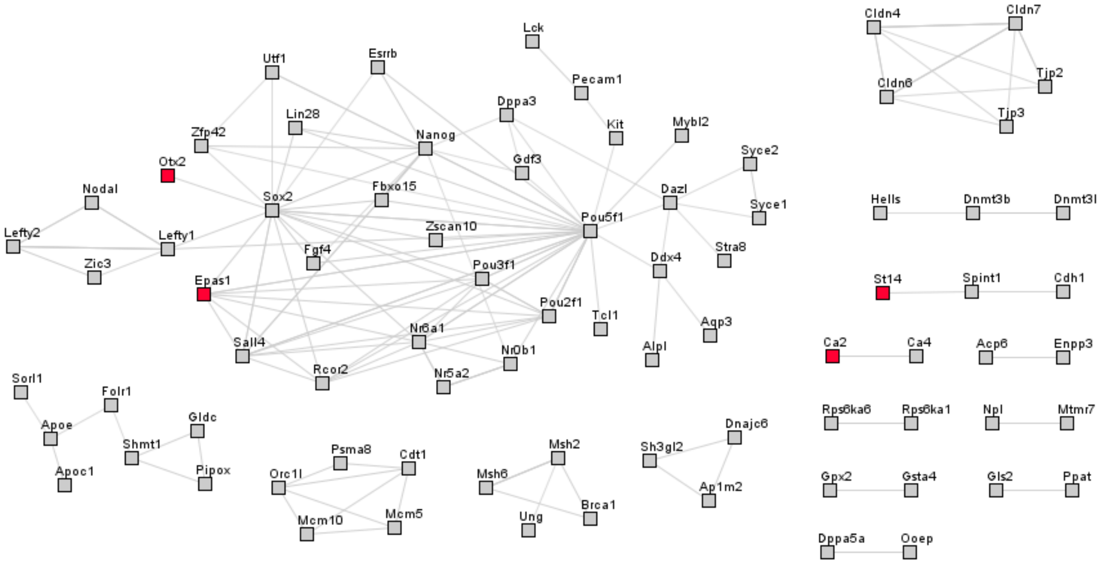

M. Highlight of histone methylation status in MEF of genes in the mouse network of most significantly upregulated genes in ES and iPS cells compared to MEF.

Green: H3K4me3 (activation); Orange: H3K4 and H3K27 (bivalent); Red: H3K27 (repression); Black: no mark; Grey: not tested

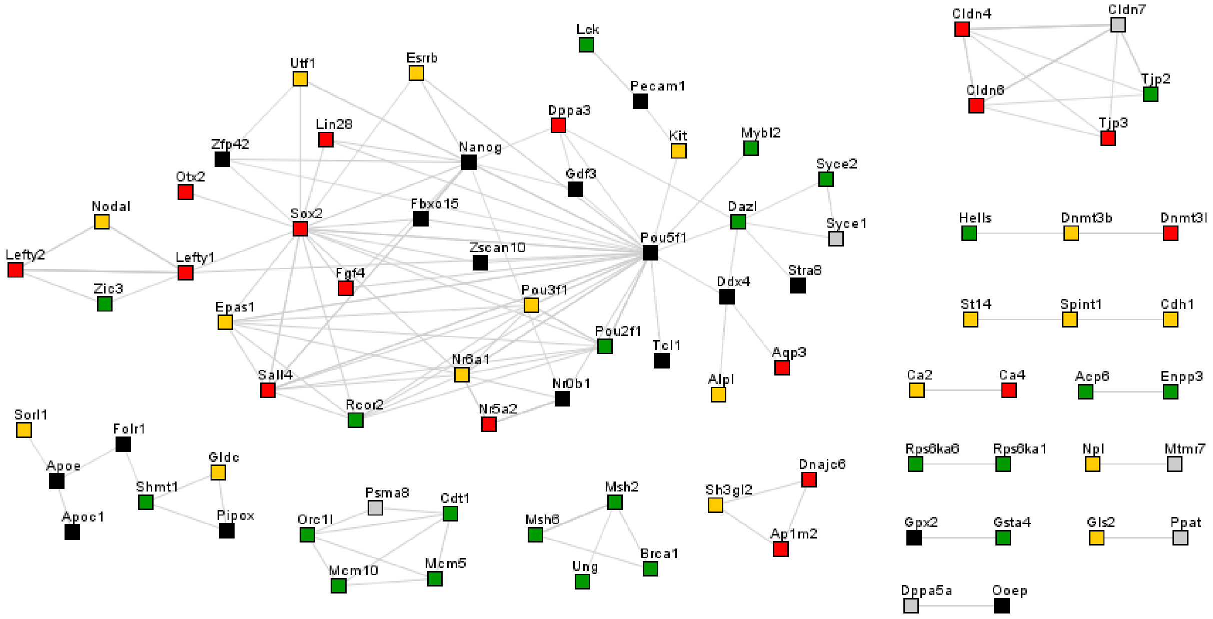

N. Highlight of histone methylation status in mouse ES cells of genes in the mouse network of most significantly upregulated genes in ES and iPS cells compared to MEF.

Green: H3K4me3 (activation); Orange: H3K4 and H3K27 (bivalent); Red: H3K27 (repression); Black: no mark; Grey: not tested

As expected, most genes have the active mark H3K4 in ES cells.

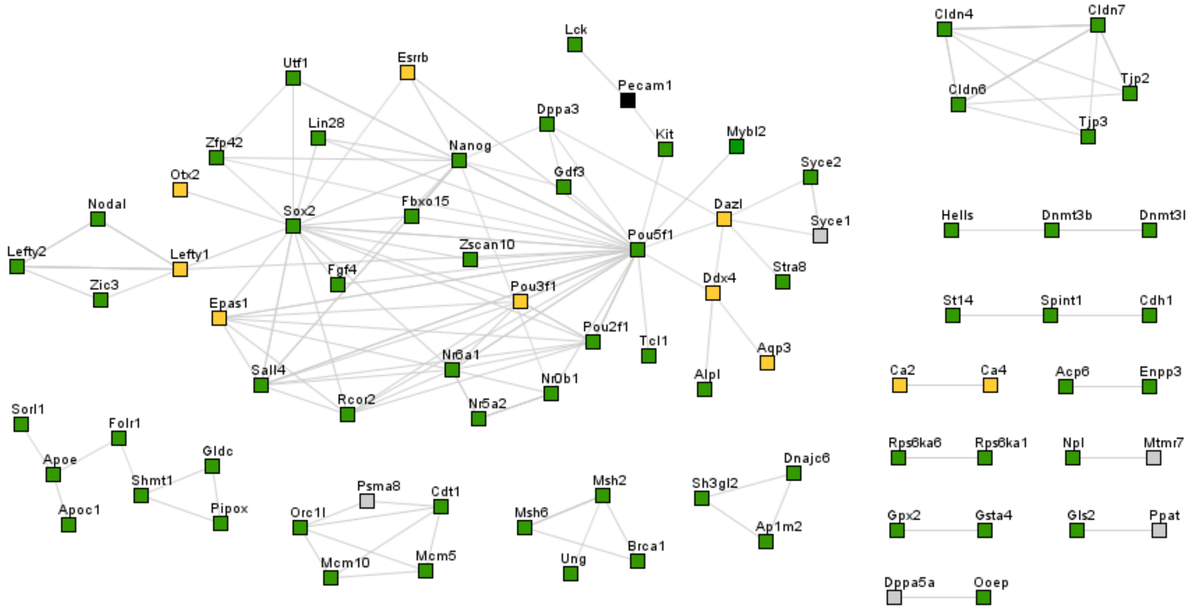

O. Highlight of genes who are members of Pou5f1 protein-protein interaction network in the mouse network of most significantly upregulated genes in ES and iPS cells compared to MEF.

Red: member of Pou5f1 interaction network described at Debbie et al [ref]. Blue: member of Pou5f1 interaction network described at Pardo et al [ref]. Purple: member of both described Pou5f1 interaction networks.

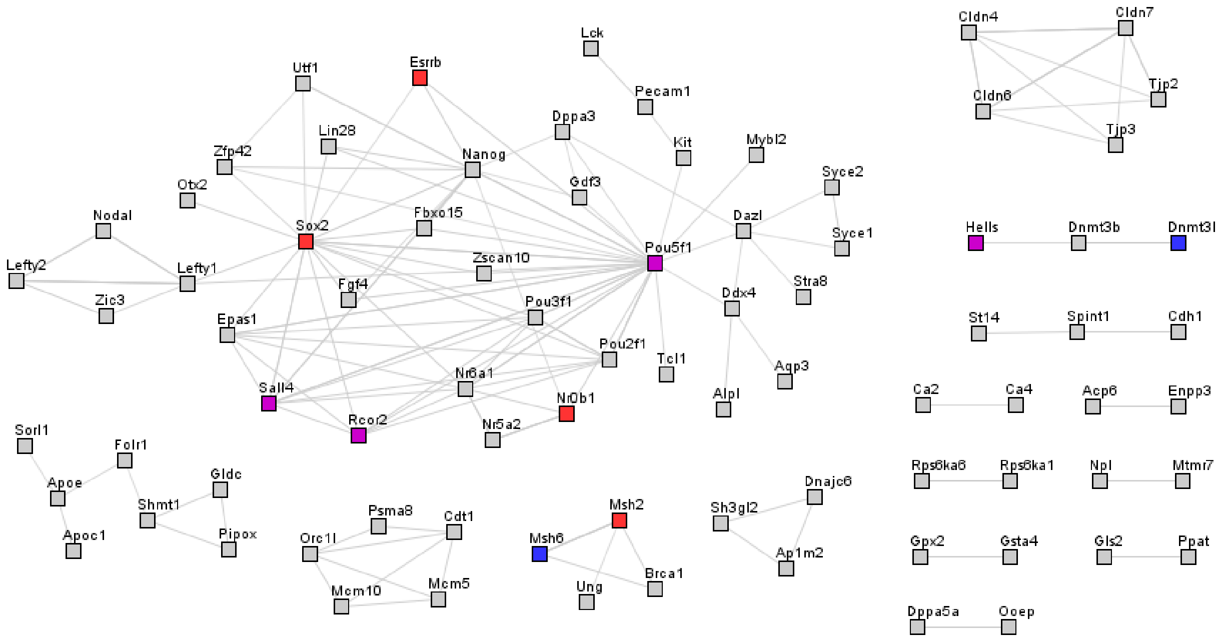

Supplement: Figure S6 — Mouse network of genes upregulated in both ESCs and iPSCs compared to MEF - Overlap with transcription factors binding and chromatin marks in ESCs. (1.01 MB PDF) [file pone.0012664.s007.pdf]
