# Supplementary material for: Analysis of Human and Mouse Reprogramming of Somatic Cells to Induced Pluripotent Stem Cells. What Is in the Plate?
Source: PLoS One. 2010 Sep 17;5(9):e12664. doi: 10.1371/journal.pone.0012664 (PMC2941458; doi:10.1371/journal.pone.0012664)

Figure S7. Level of reproducibility of the observation of a gene expression change between MEF and iPSCs or ESCs within the most significant changes in each studied dataset.

Highlight of the level of reproducibility of the presence of genes in the mouse network of most significantly upregulated genes in ESCs and iPSCs compared to MEF in all available comparisons between (A) ESCs to MEF (B) iPSCs to MEF.

A. Highlight of the level of reproducibility of the presence of genes in the mouse network of most significantly upregulated genes in ESCs and iPSCs compared to MEF in all available comparisons for ESCs to MEF.

red: gene in top 1000 genes most regulated in ESCs compared to MEF in 100% of the experiments available; orange: 81%-99% of the experiments available; yellow: 71%-80% of the experiments available; light green: 61%-70% of the experiments available; green: 51%-60% of the experiments available; dark green: 44%-50% of the experiments available.

Not all genes of this network appear as the most significantly upregulated genes in ESCs compared to MEF. Interestingly, the core network is also the highest confidence part.

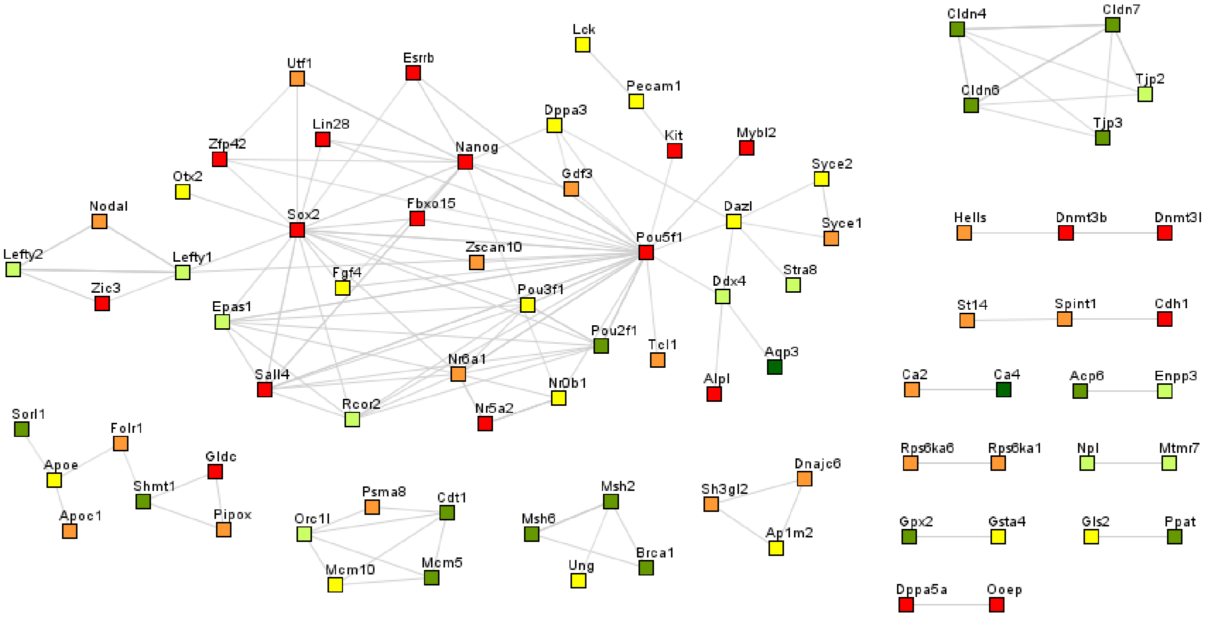

B. Highlight of the level of reproducibility of the presence of genes in the mouse network of most significantly upregulated genes in ESCs and iPSCs compared to MEF in all available comparisons for iPSCs to MEF.

red: gene in top 1000 genes most regulated in iPSCs compared to MEF in 100% of the experiments available; orange: 81%-99% of the experiments available; yellow: 71%-80% of the experiments available; light green: 61%-70% of the experiments available; green: 51%-60% of the experiments available; dark green: 44%-50% of the experiments available.

This reflects that a number of genes even in the core pluripotency network are not found among the top 1000 changes in all experiments.

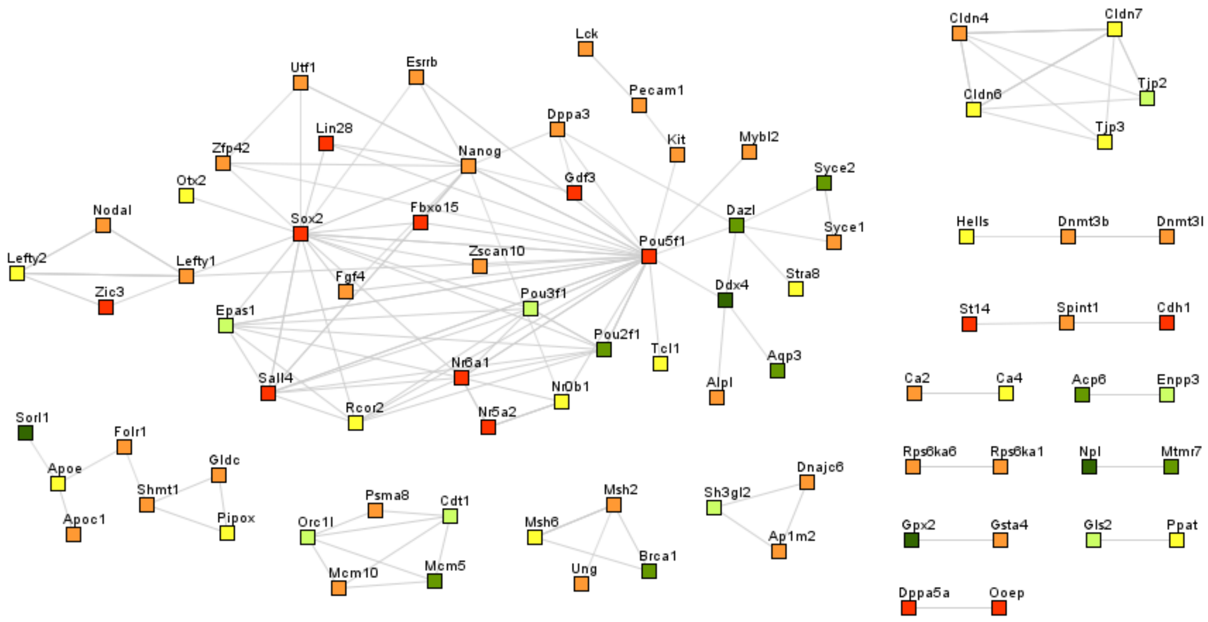

Supplement: Figure S7 — Level of reproducibility of the observation of a gene expression change between MEF and iPSCs or ESCs within the most significant changes in each studied dataset. Highlight of the level of reproducibility of the presence of genes in the mouse network of most significantly upregulated genes in ESCs and iPSCs compared to MEF in all available comparisons between (A) ESCs to MEF (B) iPSCs to MEF. (0.17 MB PDF) [file pone.0012664.s008.pdf]
