# Supplementary material for: Analysis of Human and Mouse Reprogramming of Somatic Cells to Induced Pluripotent Stem Cells. What Is in the Plate?
Source: PLoS One. 2010 Sep 17;5(9):e12664. doi: 10.1371/journal.pone.0012664 (PMC2941458; doi:10.1371/journal.pone.0012664)

Figure S8. Analysis of the differences between ESCs and iPSCs transcriptional profiles with a focus on bivalent-domain containing genes in mouse and human datasets.

Correlation coefficients for genome-wide profiles and profile of genes containing bivalent domains in ESCs for (A) Human (B) Mouse.

A. Analysis of the differences between ESCs and iPSCs transcriptional profiles with a focus on bivalent-domain containing genes in human datasets.

For each human gene expression profile ran on the Affymetrix HGU133 plus 2.0 platform the correlation coefficient of ESCs with other ESCs lines, or iPSCs or starting cell lines was calculated for both the genome-wide transcriptional profiles and the profiles of 1018 bivalent-domain containing genes.

The available iPSCs (different lines, different clones or different passages of the same cell line) are represented on the X axis. For each iPSC the correlation coefficient with the ESC for the whole array (×) and 1018 bivalent domain genes (■) is represented on the Y axes. In addition, the correlation coefficient between the ESC and starting cell for each dataset is depicted for the whole array (+) and 1018 bivalent domain genes (♦). If several ESC lines were described in the dataset, the correlation between them is represented for the whole array (*) and 1018 bivalent domain genes (●).

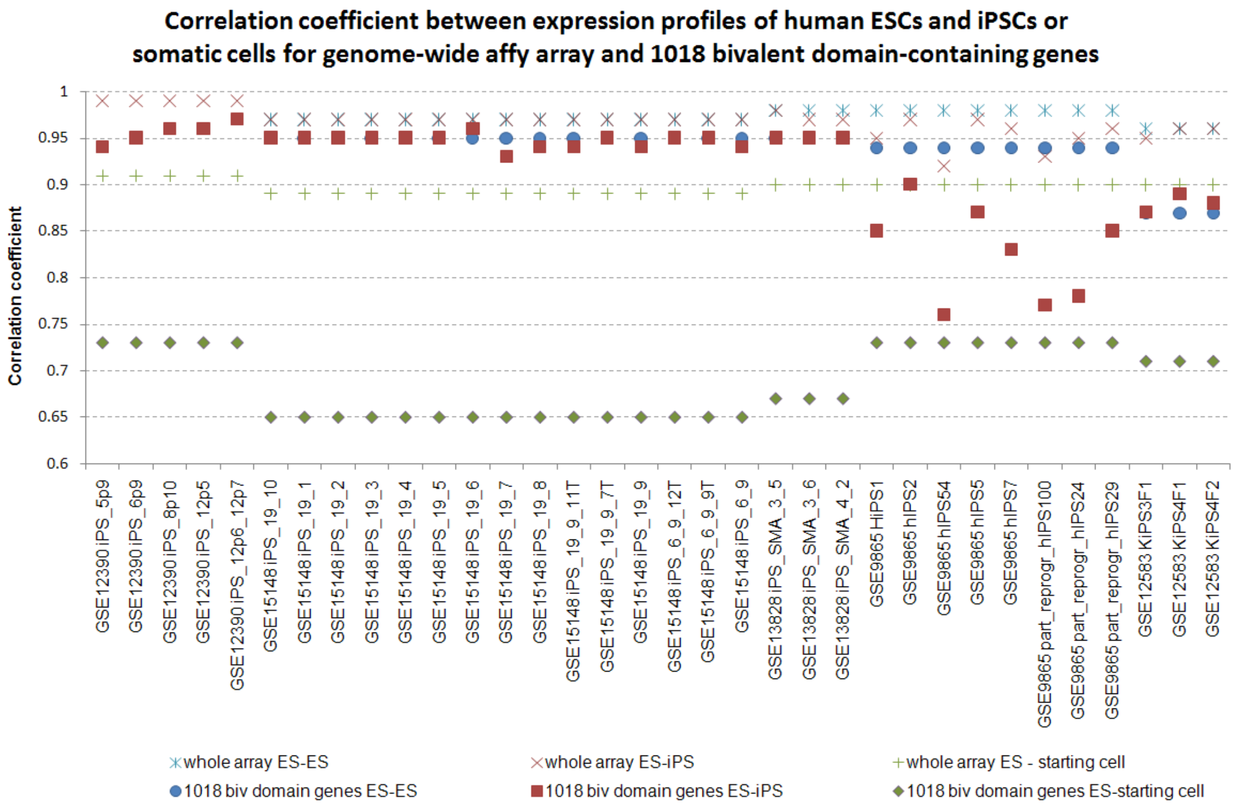

B. Analysis of the differences between ES and iPS transcriptional profiles with a focus on bivalent-domain containing genes in mouse datasets.

For each mouse dataset the correlation coefficient of ESCs with iPSC or starting cell lines are calculated for both the genome-wide transcriptional profiles and the expression profiles of 1222 bivalent-domain containing genes.

The available iPSCs (different lines, different clones or different passages of the same cell line) are represented on the X axis. For each iPSC the correlation coefficient with the ESC for the whole array (×) and 1222 bivalent domain genes (■) is represented on the Y axes. In addition, the correlation coefficient between the ESC and starting cell for each dataset is depicted for the whole array (+) and 1222 bivalent domain genes (♦).

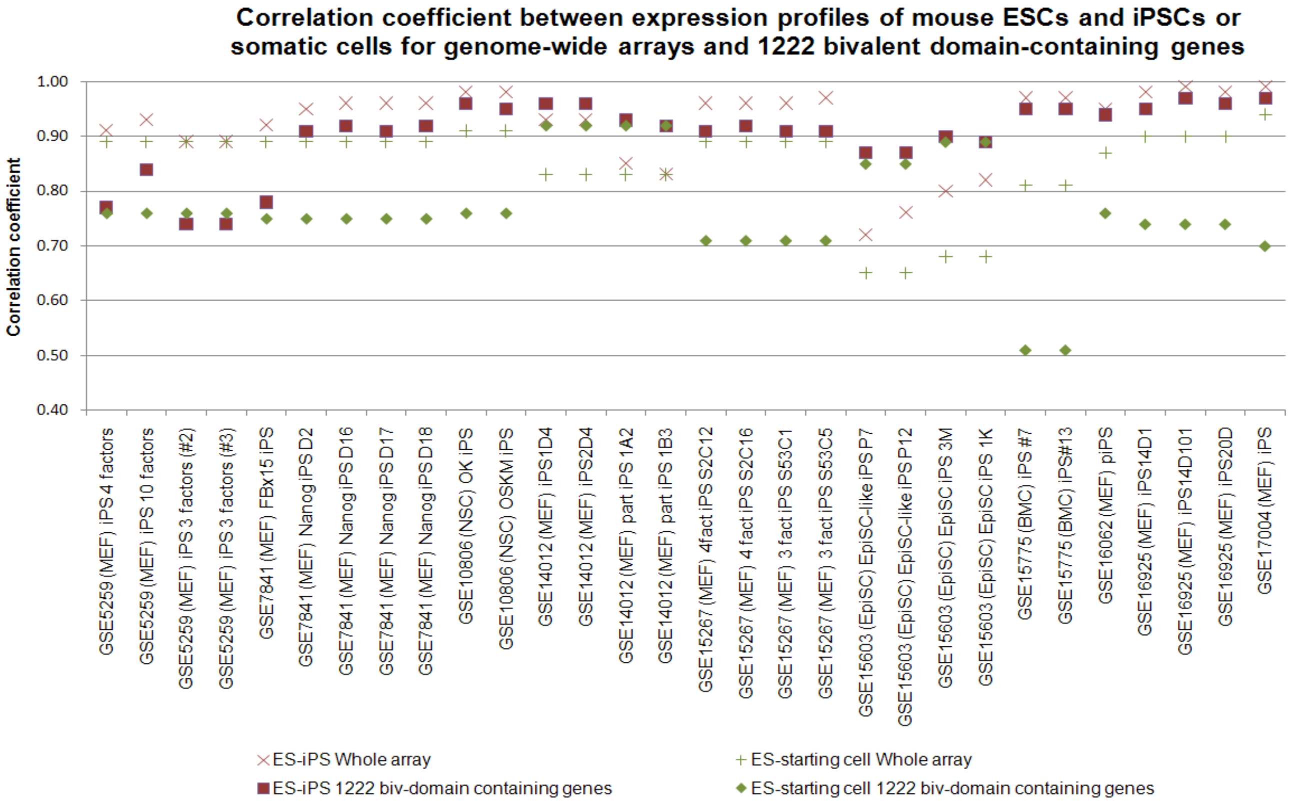

Supplement: Figure S8 — Analysis of the differences between ESCs and iPSCs transcriptional profiles with a focus on bivalent-domain containing genes in mouse and human datasets. Correlation coefficients for genome-wide profiles and profile of genes containing bivalent domains in ESCs for (A) Human (B) Mouse. (0.16 MB PDF) [file pone.0012664.s009.pdf]
