# Supplementary material for: Analysis of Human and Mouse Reprogramming of Somatic Cells to Induced Pluripotent Stem Cells. What Is in the Plate?
Source: PLoS One. 2010 Sep 17;5(9):e12664. doi: 10.1371/journal.pone.0012664 (PMC2941458; doi:10.1371/journal.pone.0012664)
Supplement: Figure S9 — Overlap of genes showing significantly different levels between iPSCs and ESCs in each dataset for Human and Mouse with the direction of the change. The expression change direction between fibroblasts and ES cells for genes which show differences between ES and iPS cells confirms that a majority of genes differently expressed between ES and iPS cells are "ES genes" which are lower expressed in iPS than in ES cells. Blue columns represent genes whose expression level is lower in ES cells than in fibroblasts, while red columns represent genes whose expression level is higher in ES cells than in fibroblasts. (0.18 MB PDF) [file pone.0012664.s010.pdf]

**Figure S9. Overlap of genes showing significantly different levels between iPSCs and ESCs in each dataset for Human and Mouse with the direction of the change.**

The expression change direction between fibroblasts and ES cells for genes which show differences between ES and iPS cells confirms that a majority of genes differently expressed between ES and iPS cells are "ES genes" which are lower expressed in iPS than in ES cells. Blue columns represent genes whose expression level is lower in ES cells than in fibroblasts, while red columns represent genes whose expression level is higher in ES cells than in fibroblasts.

**A- Human**

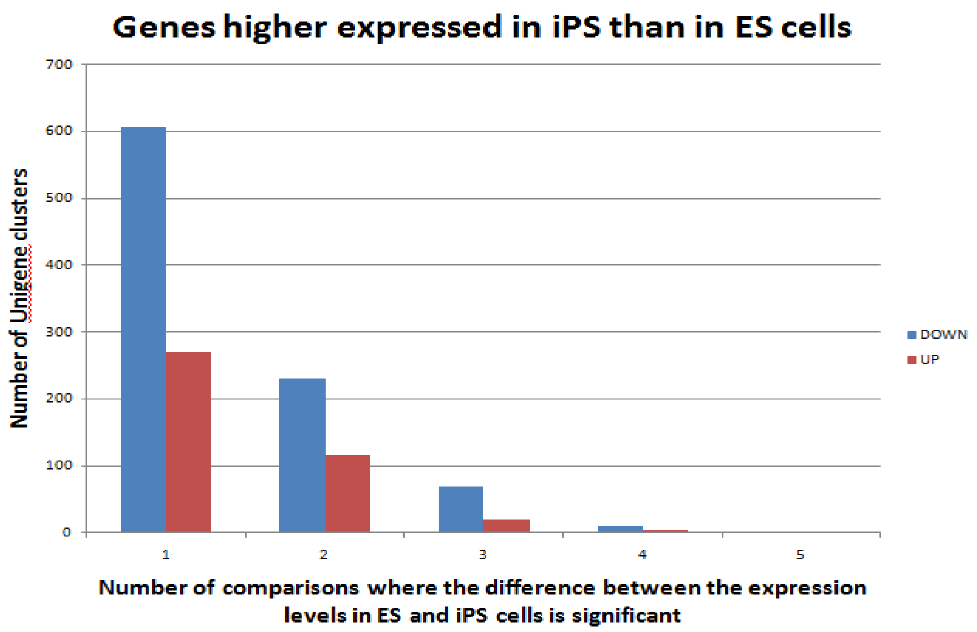


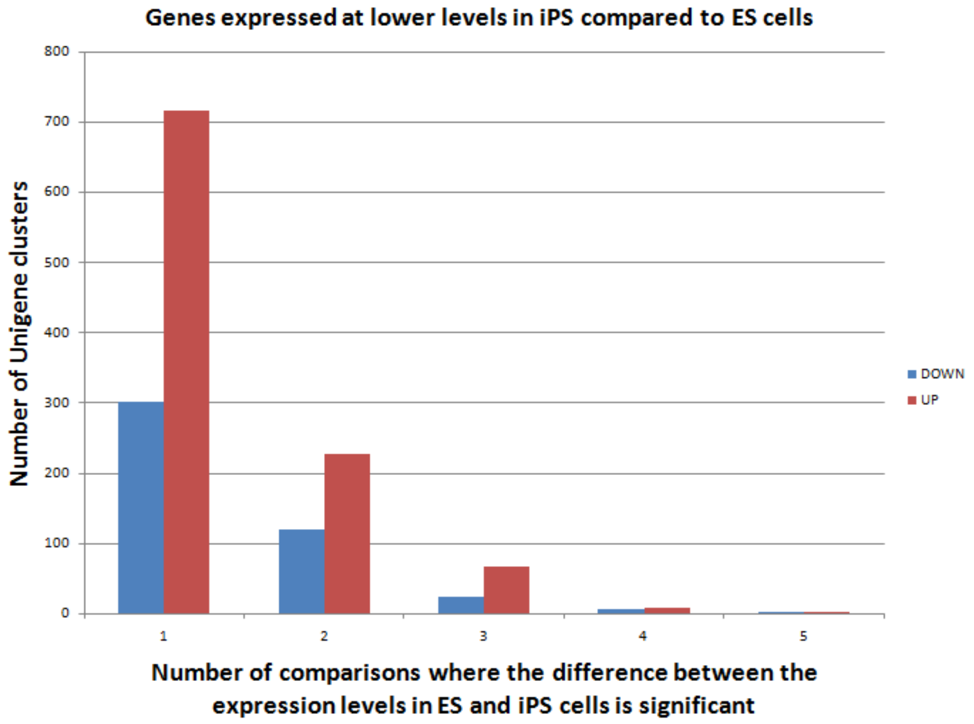

## B- Mouse

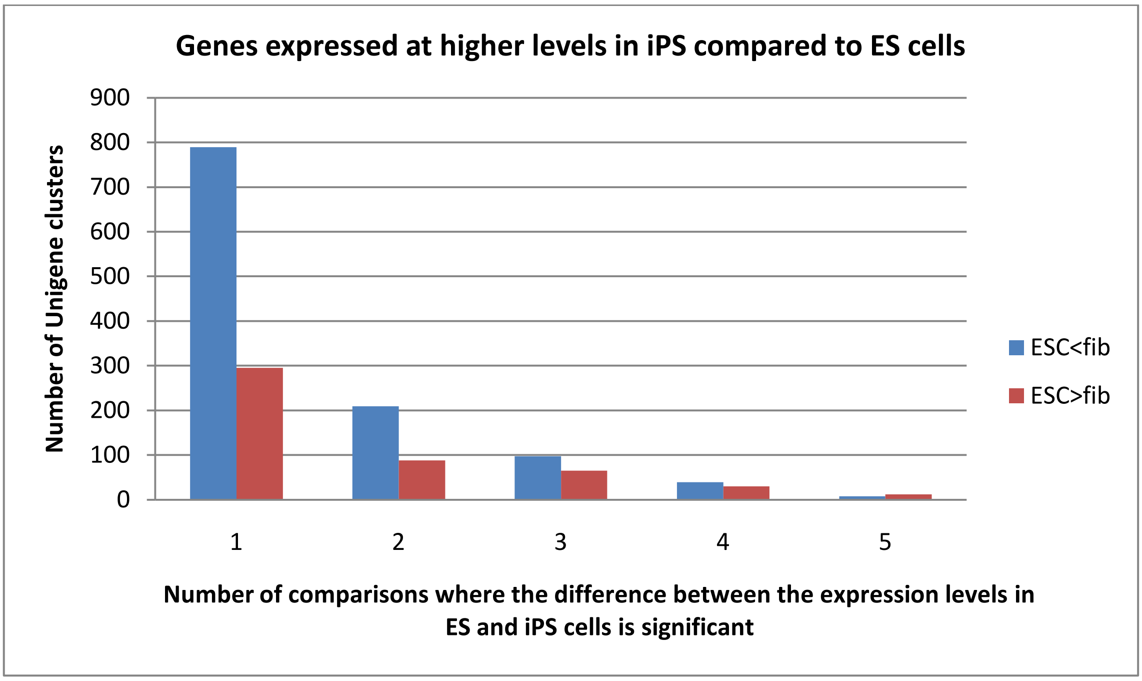

Genes expressed at higher levels in iPS compared to ES cells
Number of Unigene clusters
0
100
200
300
400
500
600
700
800
900
1
2
3
4
5
Number of comparisons where the difference between the expression levels in ES and iPS cells is significant
ESC<fib
ESC>fib


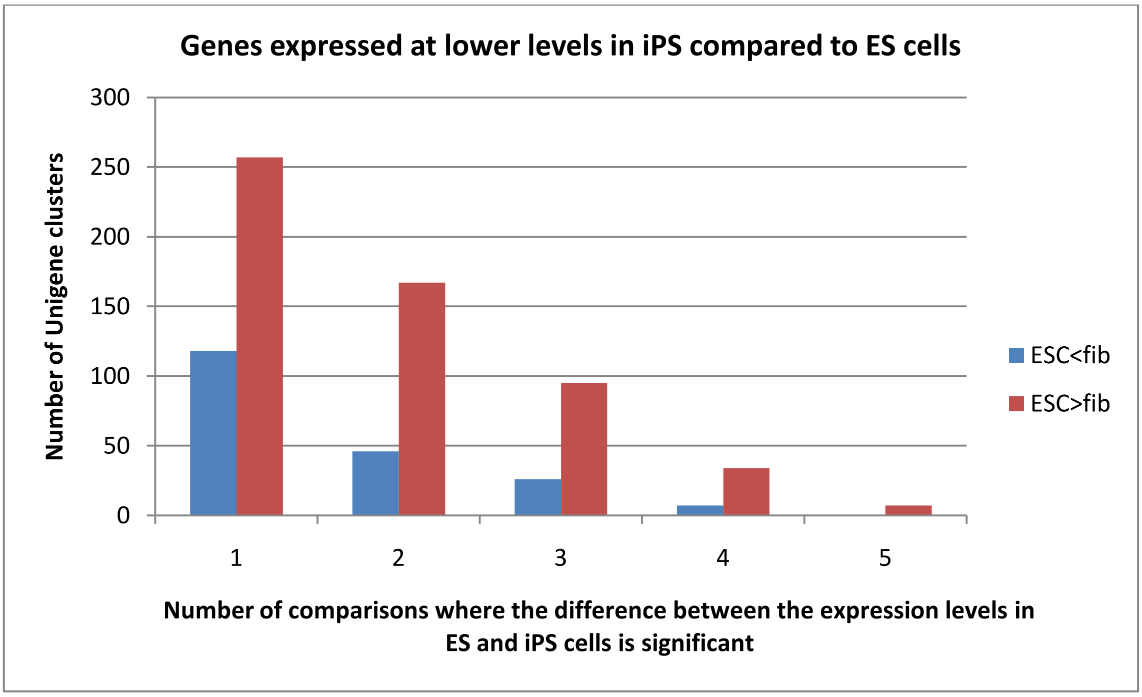

Genes expressed at lower levels in iPS compared to ES cells
Number of Unigene clusters
0
50
100
150
200
250
300
1
2
3
4
5
Number of comparisons where the difference between the expression levels in ES and iPS cells is significant
ESC<fib
ESC>fib
