# Supplementary material for: Analysis of Human and Mouse Reprogramming of Somatic Cells to Induced Pluripotent Stem Cells. What Is in the Plate?
Source: PLoS One. 2010 Sep 17;5(9):e12664. doi: 10.1371/journal.pone.0012664 (PMC2941458; doi:10.1371/journal.pone.0012664)

Figure S10. Gene expression signature of iPSCs : reanalysis of human and mouse datasets with the Chin et al. method.

The genome-wide gene expression profiles of iPSC and ESC lines were compared for human (in total 8 pairwise comparisons) and mouse (in total 15 pairwise comparisons). Genes showing a minimum fold change of 1.5 and pvalue lower than 0.05 were identified as significantly differently expressed between ESCs and iPSCs. The number of comparisons of human (or mouse) iPSCs and ESCs is represented on the X axes. The number of genes that are differently expressed between human (or mouse) iPSCs and ESCs in at least one comparison, as well as the overlap of two (or more) comparisons, is represented on the Y axis.

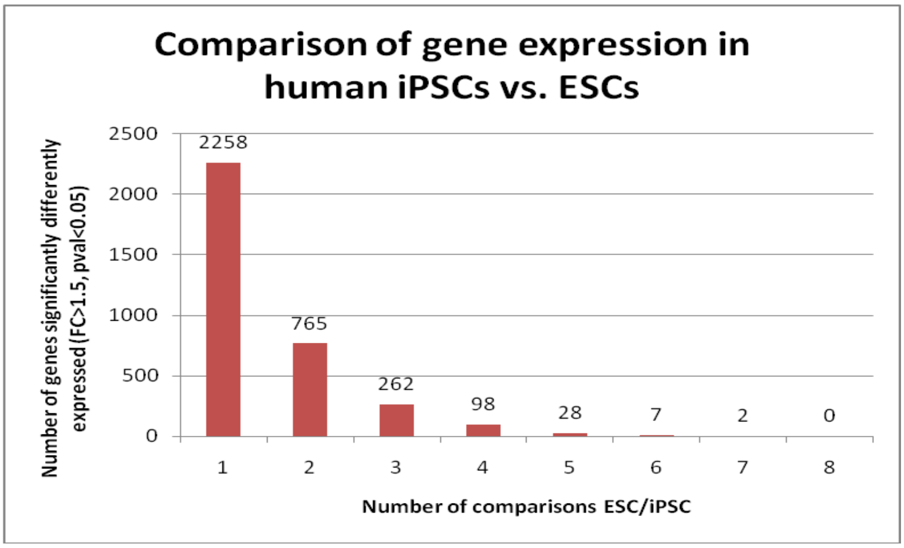

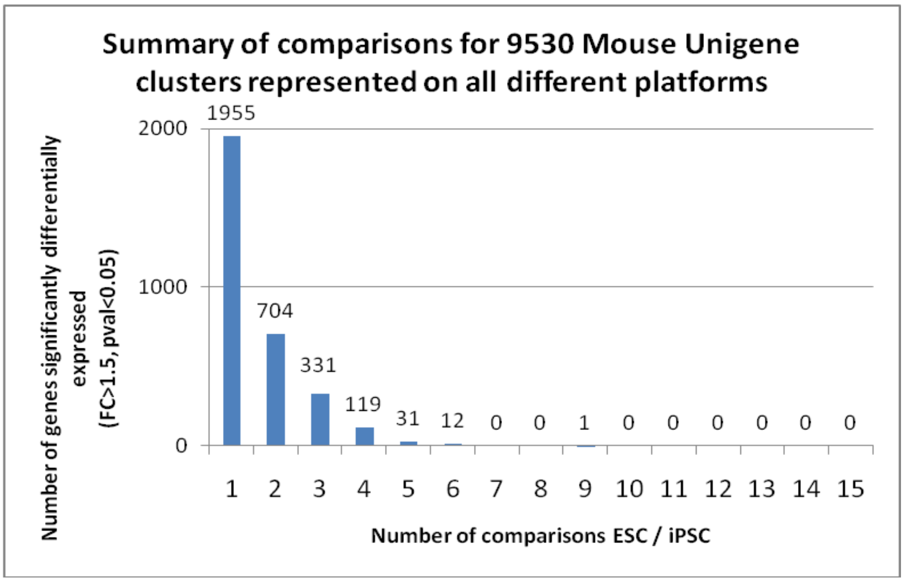

Supplement: Figure S10 — Gene expression signature of iPSCs: reanalysis of human and mouse datasets with the Chin et al. method. The genome-wide gene expression profiles of iPSC and ESC lines were compared for human (in total 8 pairwise comparisons) and mouse (in total 15 pairwise comparisons). Genes showing a minimum fold change of 1.5 and pvalue lower than 0.05 were identified as significantly differently expressed between ESCs and iPSCs. The number of comparisons of human (or mouse) iPSCs and ESCs is represented on the X axes. The number of genes that are differently expressed between human (or mouse) iPSCs and ESCs in at least one comparison, as well as the overlap of two (or more) comparisons, is represented on the Y axis. (0.09 MB PDF) [file pone.0012664.s011.pdf]
