# Supplementary material for: Analysis of Human and Mouse Reprogramming of Somatic Cells to Induced Pluripotent Stem Cells. What Is in the Plate?
Source: PLoS One. 2010 Sep 17;5(9):e12664. doi: 10.1371/journal.pone.0012664 (PMC2941458; doi:10.1371/journal.pone.0012664)

Figure S11. Number of potentially problematic bivalent domain-containing genes expressed in different human iPSC lines.

The iPSCs (different lines, different clones or different passages of the same line) from the available human datasets are represented on the X axis. For each iPSC, the number of bivalent domain-containing genes expressed in the given iPSC whereas silent in 100% (blue ■) or in at least 90% (red ■) of the human ESC lines analyzed, is represented on the Y axes.

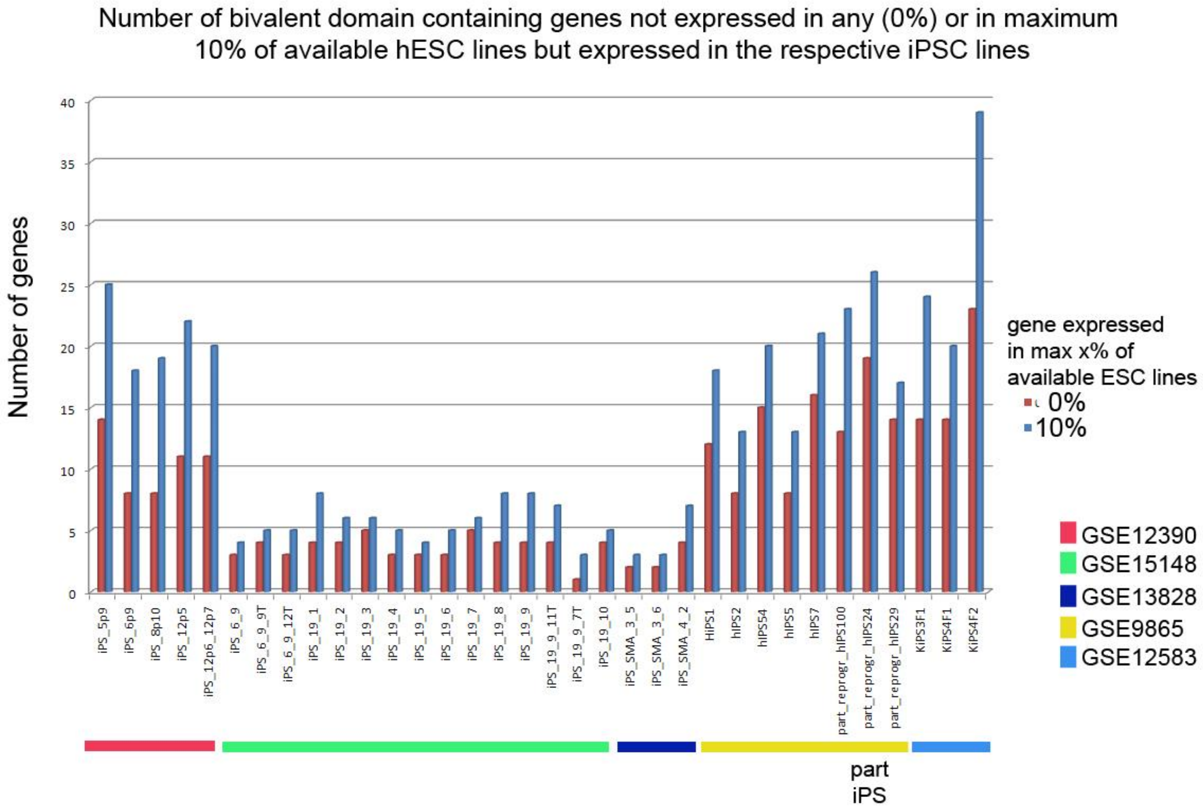

Supplement: Figure S11 — Number of potentially problematic bivalent domain-containing genes expressed in different human iPSC lines. The iPSCs (different lines, different clones or different passages of the same line) from the available human datasets are represented on the X axis. For each iPSC, the number of bivalent domain-containing genes expressed in the given iPSC whereas silent in 100% (blue) or in at least 90% (red) of the human ESC lines analyzed, is represented on the Y axes. (0.20 MB PDF) [file pone.0012664.s012.pdf]
