# Supplementary material for: Analysis of Human and Mouse Reprogramming of Somatic Cells to Induced Pluripotent Stem Cells. What Is in the Plate?
Source: PLoS One. 2010 Sep 17;5(9):e12664. doi: 10.1371/journal.pone.0012664 (PMC2941458; doi:10.1371/journal.pone.0012664)
Supplement: Figure S12 — List of tumor suppressor genes down-regulated and oncogenes upregulated in human iPSCs compared to ESCs. List of tumor suppressor genes and oncogenes whose expression levels renders iPSCs suspicious. (0.04 MB PDF) [file pone.0012664.s013.pdf]

**Figure S12: List of tumor suppressor genes down-regulated and oncogenes upregulated in human iPSCs compared to ESCs**

List of tumor suppressor genes and oncogenes whose expression levels renders iPSCs suspicious

| hgnc symbol | gene class | consensus dir fib-ips | top 1000 in fib-ips comparison | available fib-ips comparisons | average rank in comparison on Fib-iPS | FIB-iPS top 1000 in n% exp available | consensus dir change fib-es | top 1000 in fib-es comparison | available fib-es comparisons | average rank in comparison on Fib-ES | FIB-ES top 1000 in n% exp available | consensus dir change fib-preiPS | top 1000 in fib-part ips comparison | available fib-part ips comparisons | average rank in comparison on Fib-pre iPS | FIB-pre iPS top 1000 in n% exp available |
|---|---|---|---|---|---|---|---|---|---|---|---|---|---|---|---|---|
| LCK | oncogene | up in iPS | 10 | 13 | 733 | 77% | up in ES | 8 | 12 | 1273 | 67% | up in pre-iPS | 0 | 2 | 4319 | 0% |
| MYCN | oncogene | up in iPS | 11 | 13 | 734 | 85% | up in ES | 9 | 12 | 1031 | 75% | up in pre-iPS | 0 | 2 | 6145 | 0% |
| MSH2 | oncogene | up in iPS | 4 | 12 | 1103 | 33% | up in ES | 3 | 11 | 1475 | 27% | up in pre-iPS | 1 | 2 | 1058 | 50% |
| FGFR2 | oncogene | up in iPS | 9 | 12 | 1753 | 75% | up in ES | 9 | 11 | 2204 | 82% | up in pre-iPS | 1 | 1 | 173 | 100% |
| MYCL1 | oncogene | up in iPS | 4 | 13 | 3501 | 31% | up in ES | 2 | 12 | 3600 | 17% | up in pre-iPS | 0 | 2 | 5847 | 0% |
| TIAM1 | oncogene | up in iPS | 7 | 12 | 3521 | 58% | up in ES | 6 | 11 | 4230 | 55% | up in pre-iPS | 1 | 1 | 698 | 100% |
| PIM1 | oncogene | up in iPS | 2 | 12 | 4556 | 17% | up in ES | 1 | 11 | 5104 | 9% | down in pre-iPS | 0 | 1 | 6665 | 0% |
| PMS1 | oncogene | up in iPS | 0 | 13 | 5064 | 0% | up in ES | 0 | 12 | 5227 | 0% | up in pre-iPS | 0 | 2 | 3464 | 0% |
| KRAS | oncogene | up in iPS | 0 | 13 | 5575 | 0% | up in ES | 2 | 12 | 5155 | 17% | ambiguous | 0 | 2 | 3168 | 0% |
| TERT | oncogene | up in iPS | 1 | 13 | 6014 | 8% | up in ES | 1 | 12 | 5788 | 8% | up in pre-iPS | 0 | 2 | 3883 | 0% |
| KIT | oncogene | up in iPS | 2 | 13 | 6028 | 15% | up in ES | 3 | 12 | 5132 | 25% | down in pre-iPS | 0 | 2 | 3997 | 0% |
| FGF4 | oncogene | up in iPS | 1 | 13 | 6046 | 8% | up in ES | 1 | 12 | 5972 | 8% | ambiguous | 0 | 2 | 8637 | 0% |
| CDKN2A | tumor suppressor | down in iPS | 3 | 12 | 5043 | 25% | down in ES | 3 | 11 | 4950 | 27% | up in pre-iPS | 0 | 1 | 8097 | 0% |
| PTEN | tumor suppressor | down in iPS | 0 | 13 | 5143 | 0% | down in ES | 0 | 12 | 6158 | 0% | ambiguous | 0 | 2 | 6435 | 0% |
| NF2 | tumor suppressor | down in iPS | 1 | 13 | 5562 | 8% | down in ES | 0 | 12 | 5768 | 0% | down in pre-iPS | 0 | 2 | 5318 | 0% |
| RB1 | tumor suppressor | down in iPS | 0 | 13 | 5939 | 0% | down in ES | 0 | 12 | 5012 | 0% | down in pre-iPS | 0 | 2 | 7469 | 0% |

of being more tumor-prone than ESCs
